# Extinction of fish-shaped marine reptiles associated with reduced evolutionary rates and global environmental volatility

**DOI:** 10.1038/ncomms10825

**Published:** 2016-03-08

**Authors:** Valentin Fischer, Nathalie Bardet, Roger B. J. Benson, Maxim S. Arkhangelsky, Matt Friedman

**Affiliations:** 1 Department of Earth Sciences, University of Oxford, South Parks Road, OX1 3AN Oxford, UK; 2 Department of Geology, University of Liège, 14 Allée du 6 Août, 4000 Liège, Belgium; 3 Département Histoire de la Terre, Muséum National d'Histoire Naturelle, Sorbonne Universités, CR2P CNRS-MNHN-UPMC Paris 6, CP 38, 8 rue Buffon, 75005 Paris, France; 4 Faculty of Ecology, Saratov State Technical University, Politekhnicheskaya St 77, 410054 Saratov, Russia; 5 Faculty of Ecology, Saratov State University, Astrakhanskaya St 83, 410012 Saratov, Russia

## Abstract

Despite their profound adaptations to the aquatic realm and their apparent success throughout the Triassic and the Jurassic, ichthyosaurs became extinct roughly 30 million years before the end-Cretaceous mass extinction. Current hypotheses for this early demise involve relatively minor biotic events, but are at odds with recent understanding of the ichthyosaur fossil record. Here, we show that ichthyosaurs maintained high but diminishing richness and disparity throughout the Early Cretaceous. The last ichthyosaurs are characterized by reduced rates of origination and phenotypic evolution and their elevated extinction rates correlate with increased environmental volatility. In addition, we find that ichthyosaurs suffered from a profound Early Cenomanian extinction that reduced their ecological diversity, likely contributing to their final extinction at the end of the Cenomanian. Our results support a growing body of evidence revealing that global environmental change resulted in a major, temporally staggered turnover event that profoundly reorganized marine ecosystems during the Cenomanian.

## Abstract (web summary)

The extinction of the ichthyosaurs had previously been attributed to increasing competition or to the loss of their main prey. Here, Fischer *et al.* analyse phylogenetic and ecological patterns of ichthyosaur diversification and extinction, and find that the decline of the group is more likely due to climatic volatility.

## Graphical abstract

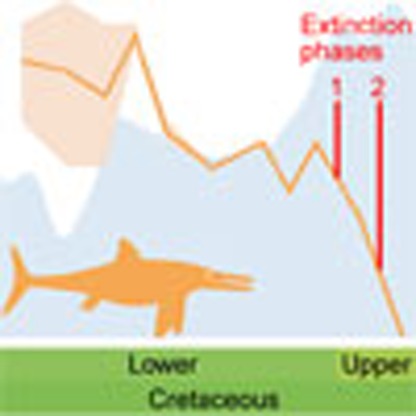

Marine predators can be regarded as an epiphenomenon related to the health of open ocean biotas; the waning and waxing of their biodiversity can thus deliver useful insights on the past fluctuations of marine ecosystems^1^. Mesozoic marine ecosystems were peculiar in hosting a diverse set of reptile clades occupying their highest trophic levels^2^; Ichthyosauria is one such emblematic clade. An increasingly well-resolved fossil record places the initial radiation of ichthyosaurs during the Olenekian stage of the Early Triassic^3^. By contrast, speculation has clouded the severity and timing of their extinction, which was first assumed to occur at the end of the Cretaceous (for example, see refs 4, 5). Subsequent analysis placed this extinction at the end of the Cenomanian^6^; ichthyosaurs thus disappeared after a 157-million-year reign, 28 million years before the end-Cretaceous extinction events that marked the demise of other numerous marine taxa of both vertebrates and invertebrates^7^. Previous analyses considered the richness of ichthyosaurs to be low in the Cretaceous and already declining since the Jurassic^8^,^9^. In parallel to low taxonomic richness, the ecological variety of Cretaceous ichthyosaurs has also been regarded as narrow^8^,^10^,^11^. As a result, the extinction of ichthyosaurs at the end of the Cenomanian was considered an isolated event associated with minor biotic changes: increased competition with other marine reptiles^12^,^13^ or teleosts^9^, or a diversity drop in their assumed principal food resource, belemnites^6^.

However, recent data challenge this view of ichthyosaur history, indicating that Early Cretaceous ichthyosaurs were taxonomically^14^,^15^,^16^,^17^, phylogenetically^18^,^19^ and—possibly—ecologically^13^,^20^ (but see ref. 11) diverse, even a few million years before their extinction^20^. These data demand re-examination of the factors associated with the waning and waxing of ichthyosaur diversity (including biases), addressing whether their extinction can be explained with existing, ichthyosaur-specific hypotheses, or was instead related to wider environmental changes in marine ecosystems of the early Late Cretaceous. We show that ichthyosaurs were diverse and disparate during the Cretaceous and faced an abrupt two-phase extinction that is associated with reduced evolutionary rates and global environmental volatility.

## Results

### Parvipelvian phylogenetic relationships

**Figure 1:**
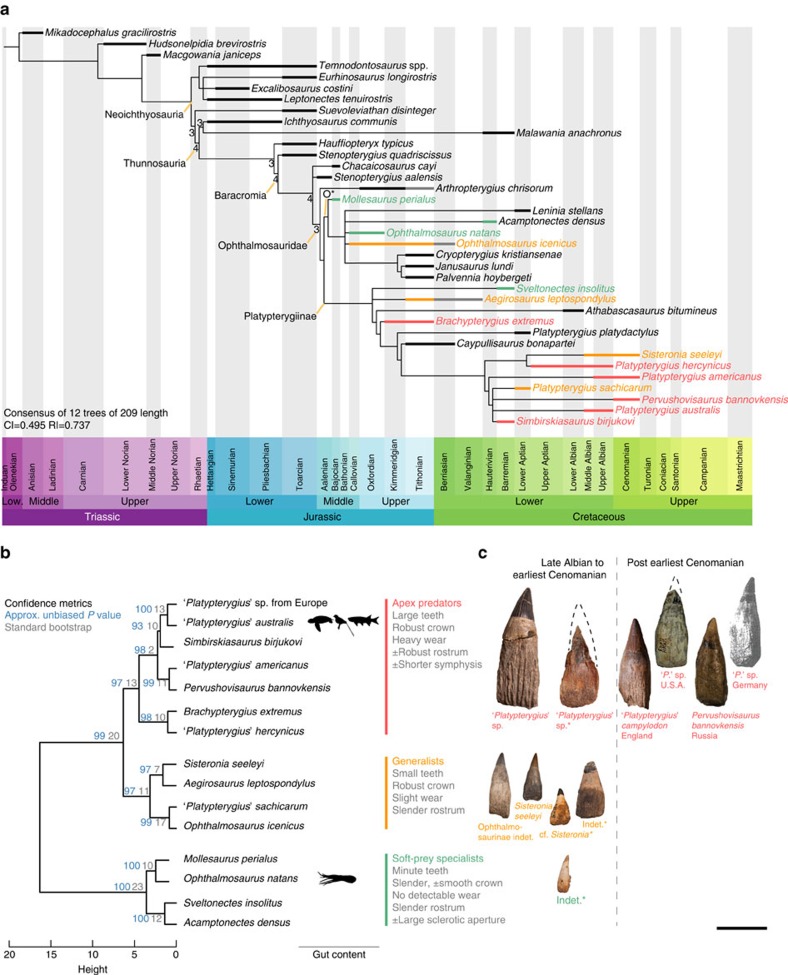
Phylogeny and ecological diversity of parvipelvian ichthyosaurs. (**a**) Time scaled strict consensus tree arising from equal weight maximum parsimony analysis. Numbers denote >1 Bremer decay indices. Grey bars denote range extensions by specimens identified at the generic level. Colour coding of taxa refers to the results of **b**. (**b**) Cluster dendrogram based on the ecological data set, with gut-content data and the general features of each guild. (**c**) Teeth representative of each guild across the Late Albian–Cenomanian interval, illustrating the ecological extinction at the beginning of the Cenomanian. ‘*Platypterygius campylodon*' and ‘*Platypterygius*' sp. from the US are early Cenomanian in age^69^, *Pervushovisaurus bannovkensis* is Middle Cenomanian in age^16^ and ‘*Platypterygius*' sp. from Germany is Late Cenomanian in age^70^. *denotes taxa from the Stoilensky/Kursk fauna. Scale bar, 50 mm.

We analysed the evolution of derived ichthyosaurs (Parvipelvia, Late Triassic to early Late Cretaceous) using novel data sets (Supplementary Methods, Supplementary Data 1–4). All analyses yielded topologies congruent with previous results from smaller data sets^19^,^21^, most notably the Jurassic origin of Cretaceous ichthyosaur lineages, the rapid divergence of Ophthalmosauridae into two distinct clades (Ophthalmosaurinae and Platypterygiinae) after the divergence of more basal lineages (*Arthropterygius chrisorum*), and the polyphyletic status of *Ophthalmosaurus* and *Platypterygius* (Fig. 1 and Supplementary Figs 1–11). For several decades, all or nearly all ichthyosaur remains from the Cretaceous have been referred to as *Platypterygius*^20^,^22^. The status of this taxon has been controversial^23^ as no phylogenetic study incorporated the type species of the genus *Platypterygius platydactylus*. Our equally weighted maximum parsimony analysis finds this species to be phylogenetically isolated from other species currently referred to as *Platypterygius* (Fig. 1 and Supplementary Figs 1 and 2). Implied weighting analysis places *P. platydactylus* as the sister taxon of a small clade of Albian–Cenomanian platypterygiines but all other species currently referred to as *Platypterygius* belong to another clade of Cretaceous platypterygiines (Supplementary Fig. 5). It is still premature to make a taxonomic decision on *Platypterygius*. However, the practise of assigning Cretaceous ichthyosaur remains to *Platypterygius* by default should be strictly avoided. The diversity dynamics of derived ichthyosaurs should be analysed at the species level rather than at genus level or above to circumvent these issues (see below).

Nodal support values within Ophthalmosauridae are smaller than those found by other analyses using smaller data sets^18^,^19^; this probably results from incorporation of numerous ophthalmosaurid taxa, many of which are based on substantially incomplete remains. However, because both phylogenetic accuracy and macroevolutionary inferences are positively impacted by increased taxon sampling^24^,^25^, and because of strong agreement on the parvipelvian tree topology between previous and present maximum parsimony analyses and Bayesian analyses, both in terms of topology and the timing of cladogenesis (see Supplementary Figs 1–12), we are confident in the adequacy of our new detailed data set and results to answer the macroevolutionary questions.

### Cretaceous ichthyosaur diversity and disparity

**Figure 2:**
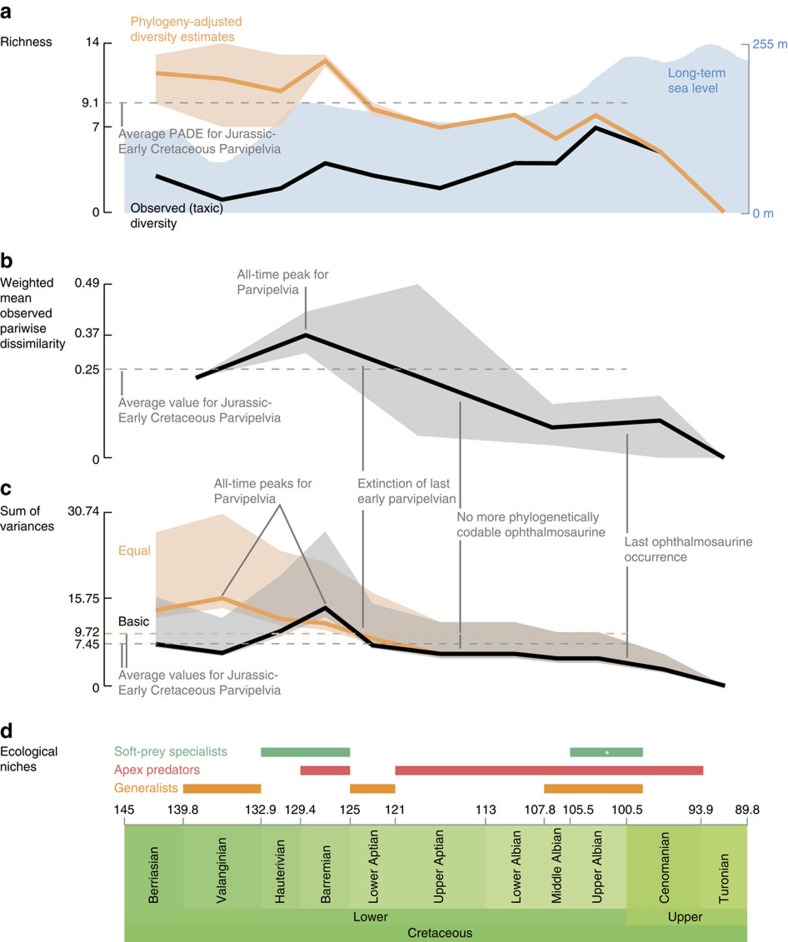
Ichthyosaur diversity through the Cretaceous. (**a**) Taxonomic/lineage richness. The orange thick line is the mean value per bin, while the light orange outline represents the range of values, encompassing all most-parsimonious trees, under both the ‘basic' and ‘equal' methods of branch length reconstruction (PADE, phylogeny-adjusted diversity estimate). The long-term sea-level is taken from Haq^62^. (**b**) Weighted mean observed pairwise dissimilarity compared with the Jurassic–Early Cretaceous average value. Light grey outline represents the 95% confidence interval. Bins are: Berriasian–Valanginian, Hauterivian–Barremian, Aptian, Albian, Cenomanian and Turonian. Important events and factors explaining the shape of the curve are indicated. Note the all-time disparity peak for Parvipelvia during the Hauterivian–Barremian. The average value for the Jurassic only is 0.24. (**c**) Sum of variances from the phylogenetically reconstructed data set, compared with the Jurassic–Early Cretaceous average values. The light orange and light grey outline represent the 95% confidence intervals. Again, an all-time disparity peak for Parvipelvia is recorded during the early Early Cretaceous. The average values for the Jurassic only are 7.53 (basic) and 9.38 (equal). (**d**) Ecological niches occupied per bin. *denotes data obtained from the Stoilensky/Kursk fauna.

A face-value count of observed species shows a general trend of increasing taxic richness throughout the Early Cretaceous, attaining a peak during the Late Albian (Fig. 2 and Supplementary Tables 1 and 2). Richness in the Late Albian is similar to that of well-sampled Jurassic stages^20^, but then declines abruptly during the Cenomanian. High diversity is apparent throughout the entire Early Cretaceous, with a marked diversity peak in between the Valanginian and Barremian interval, followed by an apparent extinction. Contrary to observed richness, the phylogenetically adjusted diversity estimates (which include counts of phylogenetic ghost lineages) suggest that ichthyosaur diversity remained high, declining only slightly through the Early Cretaceous (Fig. 2 and Supplementary Tables 3 and 4). This indicates that the apparent post-Barremian diversity loss observed in face-value species counts is an artefact of poor fossil-record sampling.

Disparity metrics calculated from phylogenetic character distributions (weighted mean pairwise dissimilarity and sum of variances including ‘ancestors') are congruent and have trajectories broadly matching that for phylogenetic diversity estimates (Fig. 2, Supplementary Tables 5 and 6 and Supplementary Data 4–6). Diversity and disparity metrics record high values during the Valanginian–Barremian interval, reflecting the co-occurrence of diverse platypterygiine lineages, ophthalmosaurines (*Acamptonectes densus* and *Leninia stellans*) and the archaic early parvipelvian *Malawania anachronus*. Although phylogenetic characters contain a strong signal related to phylogenetic distance^26^, we note that these taxa also show divergent skeletal architecture (Supplementary Figs 13–15), consistent with the observation of high disparity. Surprisingly, the Valanginian–Barremian interval records the highest disparity values for the entire history of Parvipelvia, with much higher values than the average for the entire Jurassic–Early Cretaceous interval (Fig. 2). Early Jurassic parvipelvians are not sampled at the species level, but all genera are represented in the data set (Supplementary Tables 1 and 2; Supplementary Methods); we do not anticipate that the inclusion of additional Early Jurassic species would substantially alter these results.

Disparity is decoupled from taxic/phylogenetic diversity from the Aptian onwards, declining steadily to values well below the Jurassic–Early Cretaceous average (Fig. 2). Nevertheless, it is possible that late Aptian–Albian disparity was higher than estimated here, because no ophthalmosaurine (youngest record at the Albian–Cenomanian boundary^18^) from that interval could be coded into the phylogenetic data set; disparity values for those bins thus only rely on platypterygiines. This disparity decrease may therefore have occurred later and more abruptly than suggested by our estimates (Fig. 2). After the earliest Cenomanian, ichthyosaurs were clearly reduced to a very limited range of morphologies with low disparity (Supplementary Figs 13–15).

### Evolutionary and extinction rates

**Figure 3:**
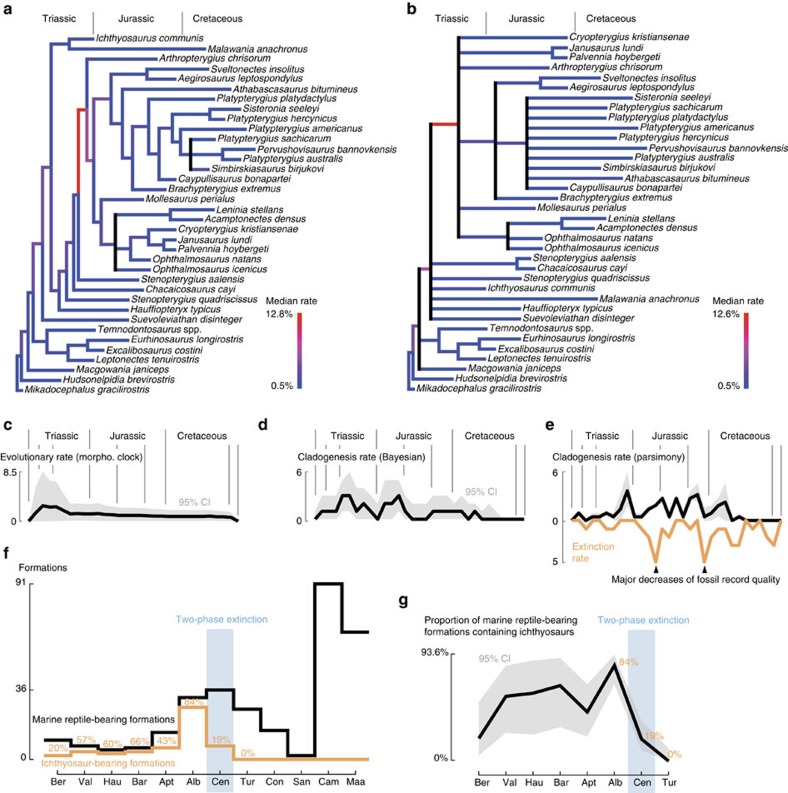
Evolution and extinction rates for parvipelvian ichthyosaurs. (**a**) Median rate of morphological evolution (morphological clock) arising from the constrained Bayesian inference. (**b**) Median rate of morphological evolution (morphological clock) arising from the unconstrained Bayesian inference. Both analyses indicate high rates in the early evolution of Parvipelvia, confined in the Triassic (**c**). (**d**) Cladogenesis rate using the time scaled trees arising from the constrained Bayesian inference. (**e**) Cladogenesis rate using the time scaled trees arising from the maximum parsimony analysis and extinction rate. The light grey outline represents the range of values, encompassing all most-parsimonious trees. (**f**) Number of marine reptile-bearing and ichthyosaur-bearing formations throughout the Cretaceous. (**g**) Proportion of marine reptile-bearing formations containing ichthyosaurs throughout the Cretaceous, with calculation of a 95% confidence interval. (**f**,**g**) Indicate ichthyosaurs disappeared in a two-phase extinction during the Cenomanian, and that this extinction is not biased by the fossil record: ichthyosaurs rarefy and disappear during a time of excellent recovery potential.

**Figure 4:**
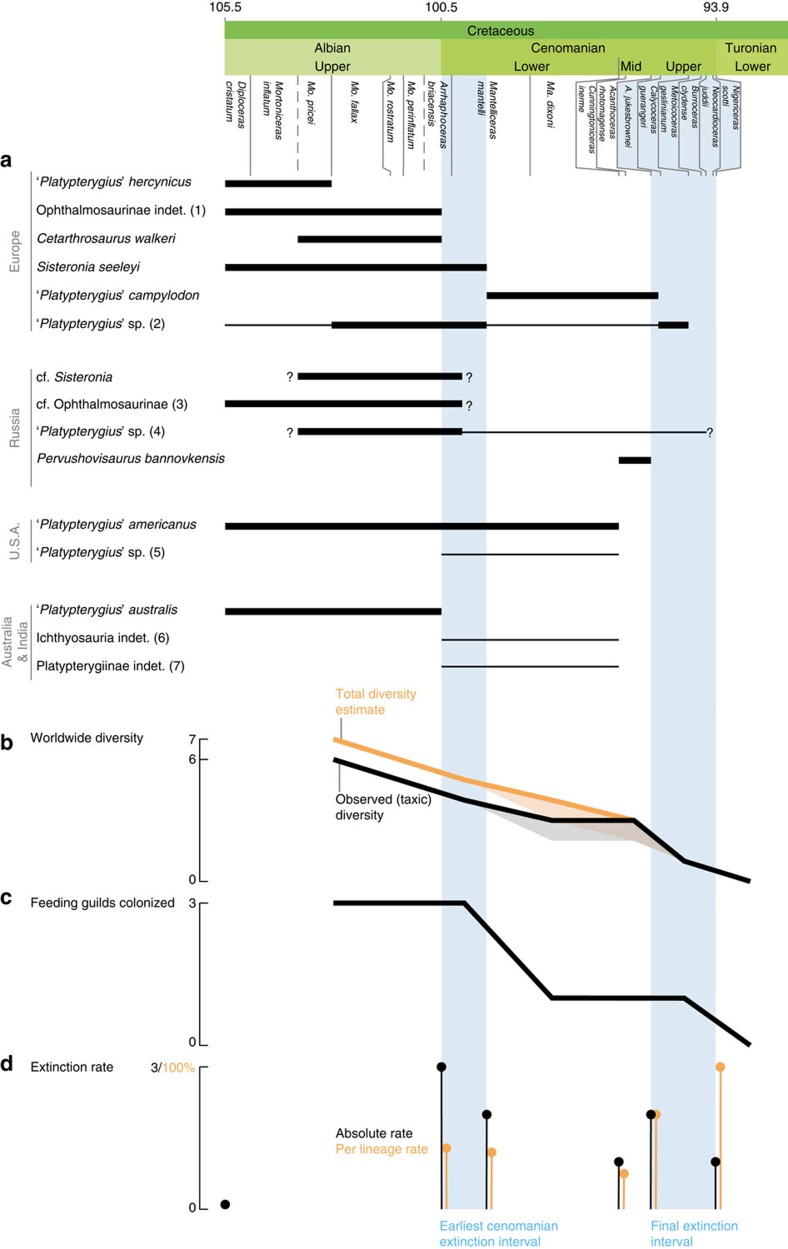
A two-phase extinction for ichthyosaurs. (**a**) Biostratigraphic ranges of the last ichthyosaur taxa. Questions marks indicate uncertainty of the stratigraphic range of the material from Stoilensky quarry (western Russia). Thin lines indicate uncertain but probable occurrence of taxa, based on the presence of compatible remains. See Supplementary Note 1 for the details and discussion on the specimens considered in the bracketed numbers. (**b**) Evolution of worldwide ichthyosaur diversity (at the species level in black and at the lineage level in orange) for each bin considered (Late Albian, earliest Cenomanian, Early Cenomanian, Mid-Cenomanian, Late Cenomanian, Turonian. The lighter colour indicates how the curve would look in *Platypterygius campylodon* is not regarded as a valid entity. (**c**) Evolution of the number of feedings guilds colonized, based on the results from the cluster analysis of ecological data. Note the sharp reduction after the earliest Cenomanian. (**d**) Extinction rate at the boundaries of each bin. Per-lineage extinction rates≥40% are recorded in the two phases of ichthyosaur extinction.

Most of the phylogenetic diversity of parvipelvians evolved during the Late Triassic–Middle Jurassic interval (Fig. 3) and not during the Cretaceous, consistent with the results of other recent studies^19^,^27^. Peaks of cladogenesis are recorded during the Late Triassic, giving rise to the ‘Neoichthyosaurian Radiation'^19^ (Figs 1 and 3 and Supplementary Tables 7–9). The ‘Ophthalmosaurid Radiation' occurs as a series of peaks spanning the Early–Middle Jurassic. We also recover a platypterygiine radiation during the Berriasian–Hauterivian stages of the Early Cretaceous. This radiation is a modest relative to those of the Triassic and Jurassic; it nevertheless, gave rise to the taxa that dominated the ichthyosaur faunas of the mid-Cretaceous and up to their final extinction in the early Late Cretaceous. Rapid rates of morphological evolution based on phenotypic characters are concentrated along the lineages connecting early ichthyosaurs to Platypterygiinae, but zero branches have rapid rates of phenotypic evolution within either Ophthalmosaurinae or Platypterygiinae (Fig. 3 and Supplementary Table 9), indicating that Cretaceous ichthyosaurs had slow rates of phenotypic evolution. Furthermore, mean rates of phenotypic evolution decelerated earlier than rates of cladogenesis, becoming low from the Early Jurassic onwards (Fig. 3). Therefore, low rates of morphological evolution coincided with low-to-null rates of cladogenesis during the Cretaceous, in a combination not seen in earlier intervals. Absolute extinction rates are elevated during the Cretaceous but the estimated per-lineage extinction rates of the Early Cretaceous are generally lower than those of the Triassic and the Jurassic. Per-lineage extinction rates are elevated at the beginning and throughout the Cenomanian (Fig. 4 and Supplementary Tables 10 and 11).

### Ecological diversity of ophthalmosaurids

Cluster analysis of ecological data (Supplementary Table 12, Supplementary Methods and Supplementary Data 7) recovers three main ecomorphological groups, further divided into a range of subgroups, and supported by significant approximated unbiased *P* values (Fig. 2). The first group is characterized by minute recurved teeth with a smooth and slender crown and no detectable wear. Two of them are ophthalmosaurines, with a large sclerotic aperture, and preserved gut content in one of them (*Ophthalmosaurus natans*) consists of only soft, unshelled coleoid remains^28^. We propose that these ichthyosaurs had a restricted diet of small, soft-prey items and were unlikely to process large prey items into smaller pieces; we term this group soft-prey specialists (which probably also incorporate the ‘specialized ram feeders' of ref. 11). The second group is the most speciose, contains only platypterygiine ichthyosaurs, and is characterized by large and robust teeth, heavy apical wear and quite often a robust (dorsoventrally deep, which better resists torsional stresses^29^) rostrum and possibly a relatively shorter symphysis. One member, ‘*Platypterygius australis*'^13^, has been found with remains of birds, turtles and fishes in its gut. We propose this group fed on a wide range of prey, including other vertebrates; we term this group apex predators. All species currently referred to as *Platypterygius* except ‘*Platypterygius sachicarum*' unite in this cluster. This grouping could indicate that these species superficially resemble each other because of ecology rather than shared ancestry. The third group contains medium-sized ichthyosaurs with a slender rostrum, bearing small teeth with a robust crown and slight wear; we propose this group preyed on a wide range of small animals. Because they share features with the two other groups, we term this group generalists. Subgroups of the cluster are supported by significant *P* values as well, but do not appear to be supported by radically distinct features. If anything, these groupings probably reflect subtle differences that could allow niche partitioning between coeval taxa. The stratigraphic distributions and counts of feeding guilds through time should be a reliable measure of ecological disparity regardless of the accuracy of our interpretations of their specific diets.

The stratigraphic distributions of our feeding guilds suffer from the same biases as observed diversity and both are broadly correlated. For example, the absence of multiple co-occurring guilds in the Berriasian–Hauterivian and Aptian–Lower Albian intervals likely reflects the poor fossil records of these intervals. Mitigating bias is difficult here, as reconstruction of ancestral ecological niches defies the principle of ecological convergence, which was widespread in marine tetrapods^10^,^30^. It is, however, possible to infer the presence of a guild by using the features that appear relevant to identify the different clusters. This approach leads us to propose that the Albian–Cenomanian boundary fauna we investigated in Stoilensky quarry, western Russia (Supplementary Figs 16–19; Supplementary Table 13 and Supplementary Methods) contains taxa occupying three distinct ecological niches. The ecological diversity of Cretaceous ichthyosaurs was high, as is especially apparently at times of better sampling. This ecological diversity declined abruptly during the early Cenomanian, despite the continued sampling of ichthyosaur specimens from all major geographic regions sampled in the late Albian and the increased preservation potential (Figs 3 and 4 and Supplementary Table 14).

### Effect of sampling and environmental changes

We used generalized least squares regression with a first-order autoregressive model and pairwise correlations to test the relationship between various biodiversity dynamics metrics, and environmental and sampling proxies (Supplementary Tables 15 and 16). All tests found poor correlations between sampling metrics and diversity variables (Supplementary Tables 17–19 and Supplementary Data 8 and 9). Akaike weights systematically place most sampling metrics among the variables with the lowest explanatory power for most diversity variables. This result suggests that the use of phylogeny-informed diversity metrics yield a signal that at least partially redresses sampling biases (but see ref. 31, as phylogenetic diversity estimates can fill ranges backwards but not forwards and are therefore prone to edge effects). The general absence of correlation between rates (cladogenesis, evolutionary and turnover), except extinction and sampling metrics is also interesting, especially in the light of recent analyses finding strong correlations between standing diversity and sampling metrics (for example, see ref. 32); this suggests that future analyses should focus on the dynamics of diversity rather than on raw values.

Broadly, bin-averaged environmental data, which represent interval-specific mean environmental conditions, do not appear to explain the diversity metrics for Cretaceous ichthyosaurs and no robust signal common to all four analyses could be recovered (Supplementary Tables 17–19). On the contrary, climate volatility variables (*∂*^18^0 and *∂*^13^C variances) are the best or among the best models for predicting the extinction rates and the per-lineage extinction rates in both data sets. A strong correlation is also found in the pairwise tests between the per-lineage extinction rates and the variances of both the *∂*^18^O and the short-term eustasy in the full data set. It is crucial to stress the importance of the extinction of ichthyosaurs in polarizing these correlations. Indeed, analyses of the full data set yielded a much larger number of significant/non-negligible correlations, especially with climate instability variables.

### Confidence in the timing and tempo of extinction

Counts of marine reptile fossil bearing formations across the Middle Cretaceous (Albian–Turonian) are among the highest of the Cretaceous, so the Cenomanian last occurrences of ichthyosaurs and their main Cretaceous ecomorphs occur during a well-sampled interval (Fig. 3). During this span, the proportion of marine reptile-bearing formations yielding ichthyosaurs decreased from 84% in the Albian to 19% in the Cenomanian and to 0% in the Turonian. Given the presence of *n*=26 marine reptile-bearing formations in the Turonian, the probability of observing zero Turonian ichthyosaur fossils given an occurrence frequency of 0.19 per formation is (1–0.19)^N^, or 0.004. Furthermore, given the observation of zero ichthyosaurs in 26 Turonian marine reptile-bearing formations, the occurrence frequency of Turonian ichthyosaurs would have to be 0.109 (that is, <10.9%) or less to give a probability of at least 0.05 of finding zero Turonian ichthyosaur fossils. To obtain a high probability (0.5) of observing no ichthyosaurs in this many sampling opportunities, the occurrence frequency would need to be no more than 0.026 (that is, <2.6%). Thus, if not actually extinct, to remain undiscovered, Turonian ichthyosaurs would need to be rare to the degree that they were ecologically insignificant. On the basis of these observations, it is likely that our estimate of the timing of ichthyosaur extinction is adequate at the timescale of our study.

## Discussion

Two deterministic hypotheses have previously been formulated to explain the latest Cenomanian extinction of ichthyosaurs: (i) a competition hypothesis, in which ichthyosaurs were outcompeted and driven to extinction by other marine reptiles^12^,^13^ or fishes^9^ and (ii) a resource hypothesis, in which ichthyosaurs vanished because of an extinction event in what was thought to constitute their main diet, soft cephalopods^6^. These scenarios invoke a single, relatively minor biotic cause for the extinction of ichthyosaurs. One major issue of the competition hypotheses are their geographical and temporal discrepancies. The earliest large-bodied mosasauroids, which are the only marine squamates that could have reasonably competed with ichthyosaurs in terms of prey type, prey size and prey location, are Middle Turonian in age^12^,^33^, thus appearing about 3 million years after the last appearance of ichthyosaurs (and likely radiating to fill at least some of their niches). Ichthyosaurs and polycotylid plesiosaurs cohabited in Australian basins and the WIS since the Early Albian at least^34^,^35^, and therefore for 19 million years before the final extinction of ichthyosaurs. In the Canadian Western Interior Seaway^14^,^17^ and in Stoilensky quarry, abundant polycotylids co-occur with a diverse assemblage of ichthyosaurs. Lingham-Soliar^9^ argued that ichthyosaurs steadily declined in diversity from the Middle Jurassic onwards, based on knowledge of the ichthyosaur fossil record that was highly incomplete compared with our present understanding. In fact, many authors have previously suggested that Cretaceous ichthyosaurs were depauperate in taxonomic and/or ecological diversity^11^,^32^,^36^. Lingham-Soliar^9^ linked this decline with the radiation of teleosts and chondrichthyans, which would have slowly outcompeted ichthyosaurs in their niche of fast thunniform swimmers. However, our data demonstrate that Cretaceous ichthyosaurs were actually about as diverse (taxonomically and ecologically) as they were during the Middle–Late Jurassic, and apparently were at their most disparate phase since the Triassic. The scenario of slow but steady replacement^9^ is therefore not substantiated by the data.

The resource hypothesis alone cannot explain the trajectories of ichthyosaur diversity and disparity through time, nor the profound, but non-terminal, extinction suffered by ichthyosaurs at the beginning of the Cenomanian. However, it remains compatible with our results, because the ecological diversity of ichthyosaurs was strongly reduced after the earliest Cenomanian. Nevertheless, the last ichthyosaurs closely resemble taxa belonging to the apex predator guild, which probably relied on diverse food resources^13^, rather than focussing almost exclusively on belemnites as previously thought^10^. In sum, both the long-term competition with selected marine predator clades and the diversity drop in belemnites cannot satisfactorily explain the breadth and tempo of the extinction of ichthyosaurs, even if these factors may have had a local importance.

Our data depict a congruent picture of Cretaceous ichthyosaurs as being highly diverse but slowly evolving. Their slow rates of origination and phenotypic evolution combined with climatic volatility-forced extinction rates to erode their high Early Cretaceous diversity, as indicated by both observed and phylogeny-adjusted taxon counts (Figs 2 and 4), and despite continued sampling of the continental regions yielding Early Cretaceous ichthyosaur fossils (Fig. 3). An apparent reduction of ichthyosaur disparity during the Aptian might be the result of poor fossil-record sampling, and could be an artefact of the absence of ophthalmosaurine specimens complete enough to be included in our data set (Fig. 2, see the ‘Results' section). By contrast, inclusion of Cenomanian taxa is more representative because all the major clades that were present can be coded in the phylogeny. A major extinction event took place during the earliest Cenomanian, when a substantial part of ichthyosaur diversity vanished, eliminating Ophthalmosaurinae and most of the ecological diversity that was present in the late Early Cretaceous. Following this event, ichthyosaurs had low diversity (Figs 2 and 4), low abundance (Fig. 3) and an extremely restricted morphospace occupation (Supplementary Fig. 15), representing only a single ecological guild (apex predators), despite the presence of several ichthyosaur specimens and, more generally, good sampling indicators (Figs 1 and 3). This previously unrecognized event presumably contributed to their extinction risk and ultimate extinction during the latest Cenomanian. Adding the Cenomanian–Turonian bins has a strong effect on the results of the correlation tests. This effect suggests that Cenomanian diversity losses cannot be explained under the same paradigm as more typical ‘background' diversity fluctuations. Interestingly, climate volatility, characterized by *∂*^18^O variance, is regarded as the best explanation for the per-lineage extinction rate of Cretaceous ichthyosaurs when the full data set is considered (Supplementary Tables 17–19). This finding highlights the potential of using the variances of environmental parameters, instead of bin-averaged mean values, in understanding diversity dynamics.

The extinction of ichthyosaurs did not happen in an ecological vacuum. It has long been recognized that the Cenomanian and the Cenomanian–Turonian boundary represents a peculiar period representing the apex of numerous climatic and oceanic perturbations, with no polar ice, extremely high sea levels, unique sedimentation, strong anoxia and very high temperature and pCO_2_ (for example, see refs 37, 38, 39, 40). There is evidence for profound global environmental volatility within the Cenomanian, the most notable being the ‘mid-Cenomanian events', involving sea level fall and perturbations of geochemical cycles (for example, see refs 41, 42). As a parallel to these profound environmental events, myriad biotic turnover events occurred at the beginning, within and at the end of the Cenomanian. Most trophic levels in marine ecosystems underwent profound changes before the Cenomanian–Turonian boundary extinction; step-like declines spread over the Cenomanian are not unique to ichthyosaurs and are actually recorded in microplankton^43^,^44^, ammonites^45^,^46^,^47^, belemnites^48^ and reef builders^49^,^50^. Simultaneously, a number of marine clades underwent explosive radiations and rose to ecological dominance during the Cenomanian, including hippuritoid bivalves^49^,^50^, euteleost fishes^51^,^52^, elasmobranch chondrichthyans^53^ and marine squamates, including early mosasauroids^33^. As such, the abrupt yet staggered extinction of ichthyosaurs thus appears as just a facet of a much broader series of biotic events that are clustered in the Cenomanian stage and ending with Cenomanian–Turonian boundary extinction. Evidence from ichthyosaurs supports a growing body of evidence^33^,^47^,^52^ revealing that a major, global change-driven turnover profoundly reorganized marine ecosystems during the Cenomanian to give rise to the highly peculiar and geologically brief Late Cretaceous marine world.

## Methods

### Material examined

Analyses are based upon a survey of literature and museum collections, including a reassessment of Cenomanian material from UK (Grey Chalk Subgroup) and description of novel remains from the Albian–Cenomanian of Russia (see Supplementary Methods and Supplementary Figs 16–18). An updated systematic framework for Cretaceous ichthyosaurs and a review of Cenomanian ichthyosaur occurrences are proposed (see Supplementary Methods). We use this updated taxonomic scheme to investigate the phylogeny and diversity of ichthyosaurs through the Late Triassic–early Late Cretaceous.

Because of the wide scope of our analysis, a large number of data, results and references of primary importance for specialists is placed in the Supplementary Methods because of space constrains. We consider these data crucial for building our conclusions and we will take all possible ways to ensure the widest possible dissemination of these data.

### Phylogenetic data and analyses

We assembled a novel phylogenetic data set for parvipelvian ichthyosaurs (see Supplementary Methods); it contains 88 characters and 36 taxa and samples Ophthalmosauridae extensively at the species level (69–76% of all valid species, depending on taxonomic opinion on Late Jurassic material from Russia; 75% of all valid Cretaceous ichthyosaur taxa are incorporated in the phylogenetic data set). Character state illustrations are given in the Supplementary Methods. We first analysed this data set using maximum parsimony, using equal and implied weighting. We also submitted this data set to Bayesian inference. Characters 33, 34 and 78 were treated as ordered, as in previous analyses. The OTU list, character list and detailed analytical settings can be found in Supplementary Methods.

### Taxic and phylogenetic diversity

Mesozoic stages greatly differ in duration, which can potentially bias our analyses, especially across the Early–Late Cretaceous boundary. We divided the largest stages (Aptian and Albian) into their widely accepted substages (lower and upper Aptian; lower, middle and upper Albian), based on ammonite biostratigraphy (see Supplementary Methods). By doing so, Cretaceous bins have a mean duration 5.02 My and a standard deviation of 1.56 My (not encompassing the error margin for stage boundaries). The observed diversity is a count of the parvipelvian-specific richness for each bin, from the Norian to the Turonian, following the results of our taxonomic revision (we have updated the Paleobiology Database record accordingly, up to the specimen level for many Cretaceous stages). This diversity count should be appraised cautiously, as it embodies a mixed signal combining underlying diversity patterns with geological preservation biases and anthropogenic sampling biases. Unfortunately, the scarcity of ichthyosaur occurrences for many stages prohibits the use of subsampling methods such as rarefaction to analyse ichthyosaur diversity. Phylogenetic analyses imply the presence of unsampled ghost lineages, and are therefore useful in predicting the diversity of a group during poorly sampled intervals^51^, providing a partial correction of diversity patterns that can be interpreted cautiously as it retains some elements of bias, and introduces edge effects^31^. These methods are still rarely used, even though ichthyosaurs and many Mesozoic vertebrate clades in general have mature and robust taxonomic and phylogenetic frameworks that permit confident phylogeny-informed inference of their diversity^32^. Because methods of branch length reconstruction can drastically impact the shape of a diversity curve, we used three methods to assess the length of branches: (i) simple timescaling of each most-parsimonious tree, which implies the minimum number of ghost lineages and, thus, the minimum phylogenetic diversity (‘basic' method of Norell^54^); (ii) equal sharing of the branch lengths between stem and ghost ranges (‘equal' method of Brusatte *et al.*^55^); (iii) morphological clock using Bayesian methods. We applied the basic and equal methods to all most-parsimonious trees and extracted the median phylogenetic diversity estimate as well as 95% confidence intervals using R (paleotree, ape and strap packages; see Supplementary Methods). Then, we added the stratigraphic ranges of each taxon in the phylogeny, as well as those of the valid taxa not included in the phylogeny to obtain a phylogenetic diversity estimate at the species level for Parvipelvia across its entire history (Late Triassic–early Late Cretaceous).

We also estimated branch lengths using Bayesian inference in MrBayes v3.2.4 (ref. 56). In addition to the analysis described above, we estimates branch lengths using a semi-fixed tree topology (hereafter named ‘constrained'), fixing all resolved nodes of the consensus tree of the maximum parsimony analysis, thus letting the program infer both branch durations and the ambiguous parts of the maximum parsimony analysis. The parameters for the latter analysis were similar to the Bayesian inference described above (see Supplementary Methods for analytical details). Morphological clock results suggest low rates of morphological evolution and thus long branches for parvipelvian ichthyosaurs. This implies, for example, the presence of multiple ophthalmosaurid lineages by the latest Triassic. While not impossible, this is currently at odds with the fossil record and the biostratigraphy of the successive outgroups of ophthalmosaurids. Bayesian estimates could thus be considered as at the ‘old' end of the spectrum of possible branch lengths. At any rate, all results are congruent in implying reduced evolutionary rates for ichthyosaurs during the Cretaceous, especially after the Hauterivian. The results of all branch length reconstruction methods can be found in the Supplementary Methods and Supplementary Fig. 1 and 2 and 7–11.

We assessed the disparity of parvipelvian ichthyosaur through time using two methods: a weighted mean and median pairwise dissimilarity using our raw phylogenetic data set and stratigraphic ranges of taxa^57^ and a sum of the variances of PCO scores from a phylogeny-informed data set, incorporating the OTUs and all hypothetical ancestors^58^. For the former method (dissimilarity), missing/scarcity of the data prevent computation of the dissimilarity and/or confidence intervals for some stages and substages. Thus, as in ref. 57, we used coarser bins here than in our other analyses, grouping stages in pairs, except the Aalenian–Bajocian–Bathonian, which are grouped together, and the Norian, Aptian and Albian, which are each considered in isolation of their long durations. We implemented a mean that is weighted relative to the number of comparable characters^59^. For the latter method (sum of variances), we followed recent attempts at mitigating the impact of missing values (for example, see ref. 58) by reconstructing this data phylogenetically and using only unambiguous ancestral character reconstructions, in Mesquite v3.01 (ref. 60). We used the most-parsimonious tree with the best stratigraphic fit (best GER and RCI indexes, see above and Supplementary Methods, Supplementary Fig. 3), thus minimizing the number of implied unsampled lineages. These methods reduced the amount of missing data from 45.3 to 5.1%. We ran principal coordinate analyses on that reconstructed data set. The sum of variances was calculated for each stage or substage and under both the ‘basic' and ‘equal' methods of branch length reconstruction. We used the first 45 axes, accounting for 95% of the variance. We then bootstrapped the data 10,000 times to get 95% confidence intervals. All calculations were performed in R.

### Ecological diversity

We built a second, independent data set using selected ophthalmosaurid taxa and a set of seven continuous characters based on nine measurements that were selected for their ecological relevance: absolute tooth size, crown shape, crown size relative to gullet diameter, relative symphysial length, snout depth, absolute sclerotic aperture (determining the size of the cornea) and tooth wear. Most studies of the palaeoecology of marine reptiles have only looked at tooth wear only qualitatively^10^,^61^. Whereas intrinsic properties of teeth (size, shape) give an idea of the optimal type/range of prey types that could be processed, wear gives indications on the actual use of teeth, although only by a single individual. We used articulated rostra to quantify the amount wear (see Supplementary Methods for the metrics used and their rationale). We submitted this data set to a cluster analysis in R using the Ward method. Data were scaled to have equal variances and transformed to a Euclidean distance matrix before clustering; see Supplementary Methods and Supplementary Data 7 for data and analytical details. We then mapped fossilized gut-content data^13^,^28^ on the cluster dendrogram to test the congruence of our results.

### Rates

To avoid the spurious correlation of time series and capture the diversity dynamics of ichthyosaurs, we estimated rates of cladogenesis, extinction and discrete-character evolution for parvipelvian ichthyosaurs through time using our data sets. Both the cladogenesis and the evolutionary (morphological clock) rates ultimately rely on morphology (via phylogenetic relationships) and first-occurrence datums. They are thus affected by incomplete information, taxonomic sampling, uncertainties in phylogenetic relationships and fossil dating, and the fluctuations of the quality of the fossil record. Extinction rates only rely on the last-occurrence datum and are thus biased by fluctuations of the quality of the fossil record. Some of these biases can only be addressed qualitatively, by cautious interpretation of resulting patterns. Nevertheless, others can be addressed quantitatively by the following measures. Uncertainties of the dating and of relationships are encompassed using all the most-parsimonious trees, and 3,000 sampled trees from the posterior distributions of our Bayesian analyses. Detailed comparisons between these rates and proxies for fossil-record biases (see below) have also been conducted; we found no significant relationships between these rates and our sampling proxies. Rates of cladogenesis were computed for both the maximum parsimony and the Bayesian inference analyses, by counting the number of cladogenesis events implied by the phylogeny in each time bin. For the maximum parsimony data set, all most-parsimonious trees and under both the ‘basic' and ‘equal' methods of branch length reconstruction were used. For the Bayesian data sets, we sampled 1,000 trees per run, resulting in 3,000 sampled trees per data set. Extinction rates were calculated as the number of taxa (with their Lazarus ranges, if any) going extinct before or at the upper boundary of each stage or substage. Per-lineage (‘relative') extinction rates are the percentage of lineages going extinct during a bin. Turnover rates are the sum of the cladogenesis and extinction rates.

### Biases and sampling metrics

A large body of literature demonstrates strong links and potentially causal relationships between the rock and fossil records, notably of marine reptiles^32^. We compare several variables of ichthyosaur diversity (observed diversity, phylogenetic diversity estimates, cladogenesis rates, evolutionary rates, extinction rates and turnover rates) with a number of a rock record proxies, for each bin: mean sea level^62^ and the number of occurrences, collections and formations of all metazoan fossils in a marine setting, all vertebrates in a marine setting, and all aquatic tetrapods in all depositional settings, downloaded from the Paleobiology Database (paleobiodb.org) before updating the Cretaceous ichthyosaur record at the specimen level in that database, in order to avoid a bias in our correlations. As these data are often not resolved at the substage level, we assigned a fraction of the Aptian and Albian data sets to each of their substages, based on their relative durations, as in ref. 58. We refrained from analysing rock area/volume because of issues of redundancy and common cause which could be difficult to identify using a data set on ichthyosaurs alone. Instead, we have also analysed the extinction of ichthyosaurs statistically, by (i) comparing a potential recovery metric (the number of marine reptile-bearing formations) with the number of ichthyosaur-bearing formations and (ii) computing confidence intervals for the extinction of ichthyosaurs as a whole. For this test, we used the simple method of Strauss and Sadler^63^, which implies constant recovery potential. The mean ichthyosaur recovery potential along their entire history is 0.76 formations per My (120 ichthyosaur-bearing formations over 157.3 My, as downloaded from the Paleobiology Database on 13 October 2015). This translates into a mean 5.34 and 3.13 formations for the Cenomanian and the Turonian, respectively, while these stages record a much higher value of 36 and 26 marine reptile-bearing formations. Integrating this higher recovery potential in the confidence interval calculation would result in smaller range extension; the Strauss and Sadler^63^ test is thus more generous towards a younger extinction for ichthyosaurs. This test gives a 95% confidence range extension of 0.99 My and of 1.52 My with a confidence of 97.5%, thus firmly placing the extinction of Ichthyosauria as a whole in the earliest Turonian at the latest.

### Environmental drivers

We investigated potential drivers of ichthyosaur diversity during the Cretaceous by running correlation tests between our diversity variables and environmental proxies. We used the mean and variance (both at short and long term, using data from Haq^62^), two measures of sea-surface temperatures and/or *∂*^18^O (refs 64, 65) per bin.

### Correlation tests

We performed pairwise correlation tests after applying generalized differencing^66^ to the relevant data series. We also fitted generalized least square linear models including a first-order serial correlation coefficient^67^ and estimated their explanatory power using the modified Akaike information criterion for finite sample sizes (AICc^68^). The performance of an intercept-only model, in which a serial correlation parameter describes a spectrum of possibilities between stationary values drawn from a normal distribution and a non-stationary random walk with step sizes drawn from a normal distribution, was also tested. We ran these analyses on the entire data set (Berriasian–Cenomanian) and on an Early Cretaceous data set excluding the Cenomanian (Berriasian–Albian) to investigate the influence of the final extinction of ichthyosaurs on factors explaining their waning and waxing of their diversity and the potential uniqueness of that event compared with their previous history.

## Additional information

**How to cite this article:** Fischer, V. *et al.* Extinction of fish-shaped marine reptiles associated with reduced evolutionary rates and global environmental volatility. *Nat. Commun.* 7:10825 doi: 10.1038/ncomms10825 (2016).

## Supplementary Material

Supplementary Information Supplementary Figures 1-19, Supplementary Tables 1-19, Supplementary Note 1, Supplementary Methods and Supplementary References

Supplementary Data 1 Character-taxon nexus

Supplementary Data 2 Nexus file for the constrained Bayesian analysis

Supplementary Data 3 Nexus file for the unconstrained Bayesian analysis

Supplementary Data 4 Phylogeny-reconstructed character-taxon nexus for morphospace analysis

Supplementary Data 5 Results of the pcoa analysis

Supplementary Data 6 Results of the pcoa analysis

Supplementary Data 7 Ecological measurements used for the cluster dendrogram analysis

Supplementary Data 8 Results of the pairwise correlation tests

Supplementary Data 9 Results of the generalised least square tests
