# Supplementary material for: Extinction of fish-shaped marine reptiles associated with reduced evolutionary rates and global environmental volatility
Source: Nat Commun. 2016 Mar 8;7:10825. doi: 10.1038/ncomms10825 (PMC4786747; doi:10.1038/ncomms10825)
Supplement: Supplementary Information — Supplementary Figures 1-19, Supplementary Tables 1-19, Supplementary Note 1, Supplementary Methods and Supplementary References [file ncomms10825-s1.pdf]

SUPPLEMENTARY FIGURES

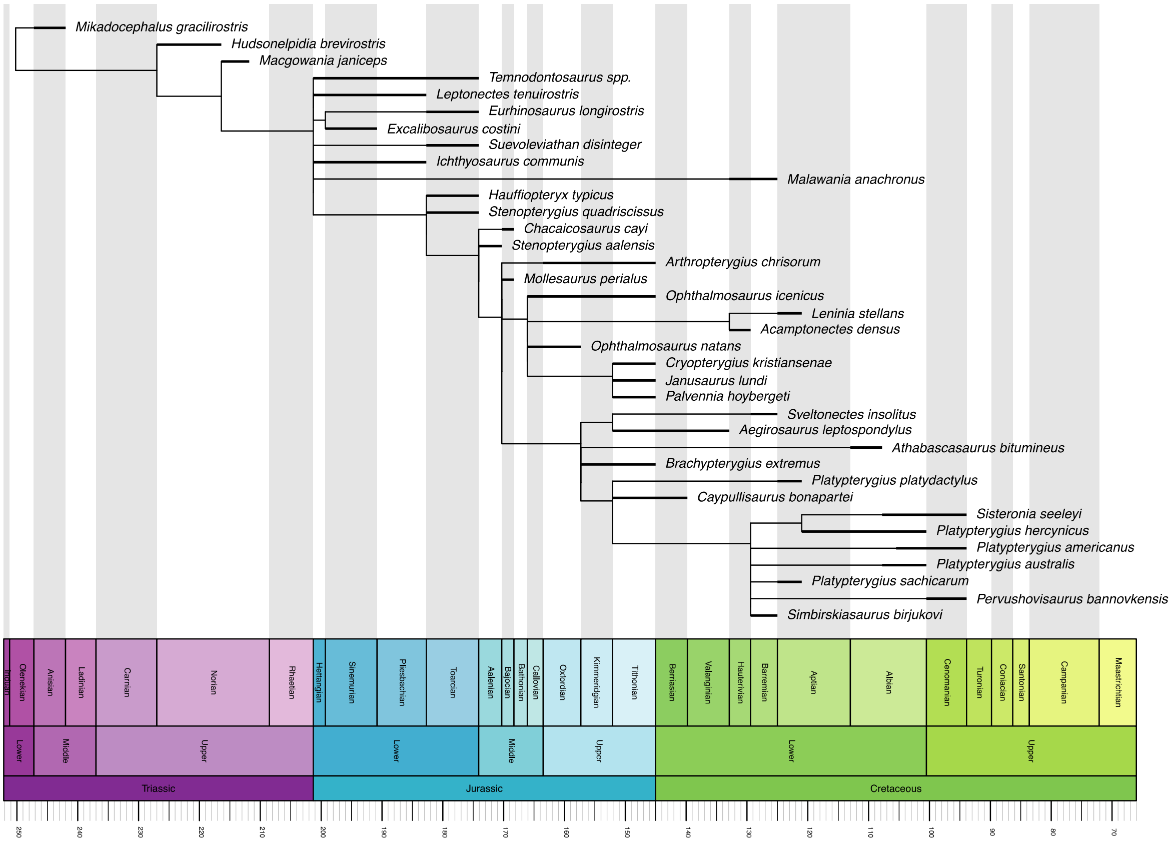

Supplementary Figure 1 | Most parsimonious tree with the best stratigraphic fit. The The tree presented is the one with the best GER (Gap Excess Ratio[1]) and SCI (Stratigraphic Congruence Index[2]) scores, in 'basic' reconstruction of branch lengths, arising from the equal weight maximum parsimony analysis**.** This analysis recovered twelve most parsimonious trees with a length of 209 steps. The strict consensus typology strongly matches those of previous attempts[3–6] and only a few differences are present. Notably, *Athabascasaurus bitumineus* is recovered as a platypterygiine slightly more derived than *Aegirosaurus leptospondylus* and *Sveltonectes insolitus*, unlike in [5]. The increase coverage of Cretaceous taxa did not destabilise the structure of the tree. These additional Cretaceous taxa are recovered as platypterygiine ophthalmosaurids, occupying various positions within this clade. The type species of *Platypterygius*, *Platypterygius platydactylus* is recovered outside the clade containing most species currently referred to as *Platypterygius*. *Sisteronia seeleyi* appears closely related to ‘*Platypterygius*’ *hercynicus*, forming a clade that is the sister clade of platypterygiines with a divided naris (‘*Platypterygius*’ *australis* + ‘*Platypterygius*’ *sachicarum* + *Simbirskiasaurus birjukovi* + *Pervushovisaurus bannovkensis*) + ‘*Platypterygius*’ *americanus*.

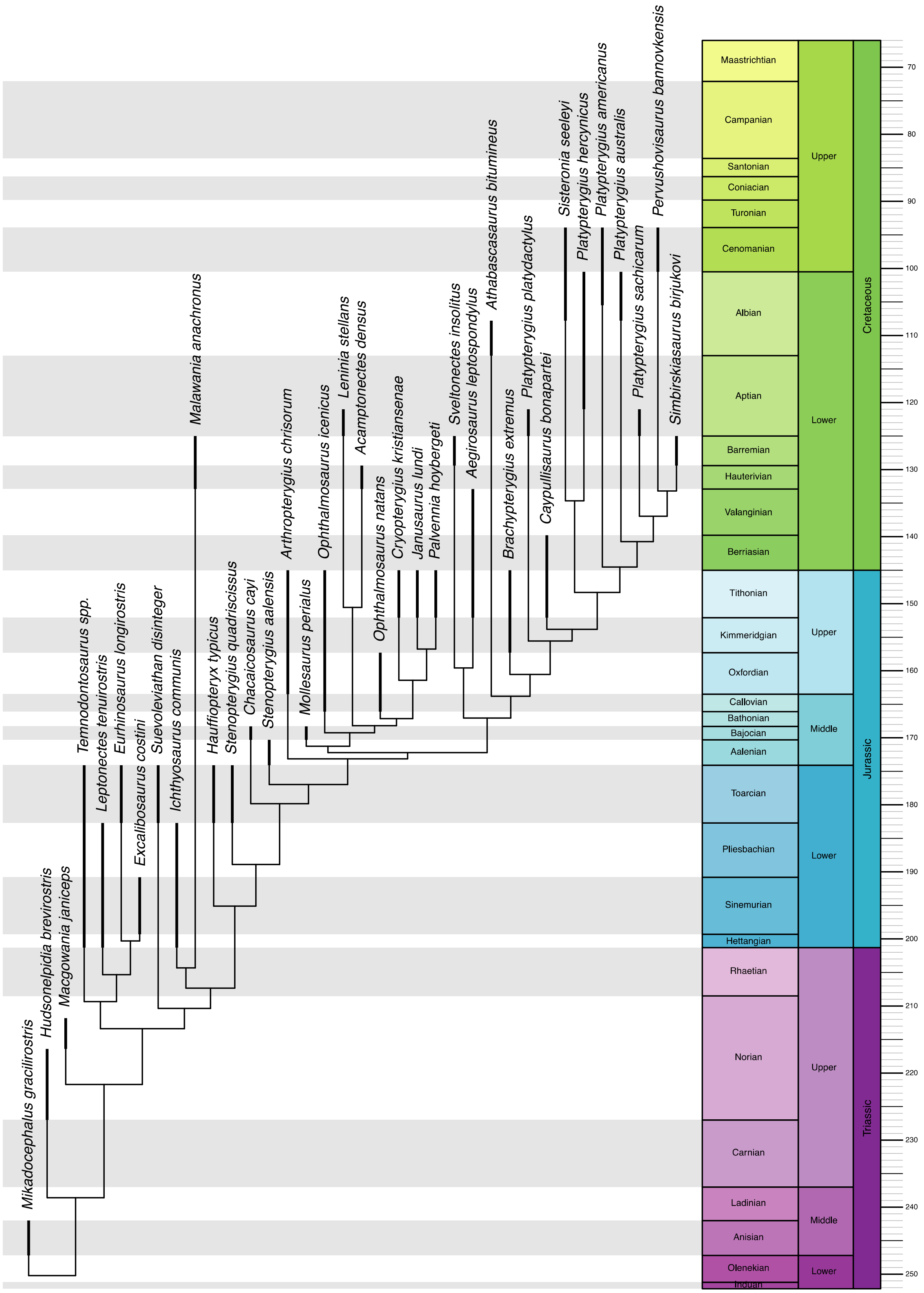

Supplementary Figure 2 | Most parsimonious tree with the best stratigraphic fit. The The tree presented is the one with the best GER and SCI scores, in 'equal' reconstruction of

branch lengths, arising from the equal weight maximum parsimony analysis. See Supplementary Figure 1 caption for details of the results.

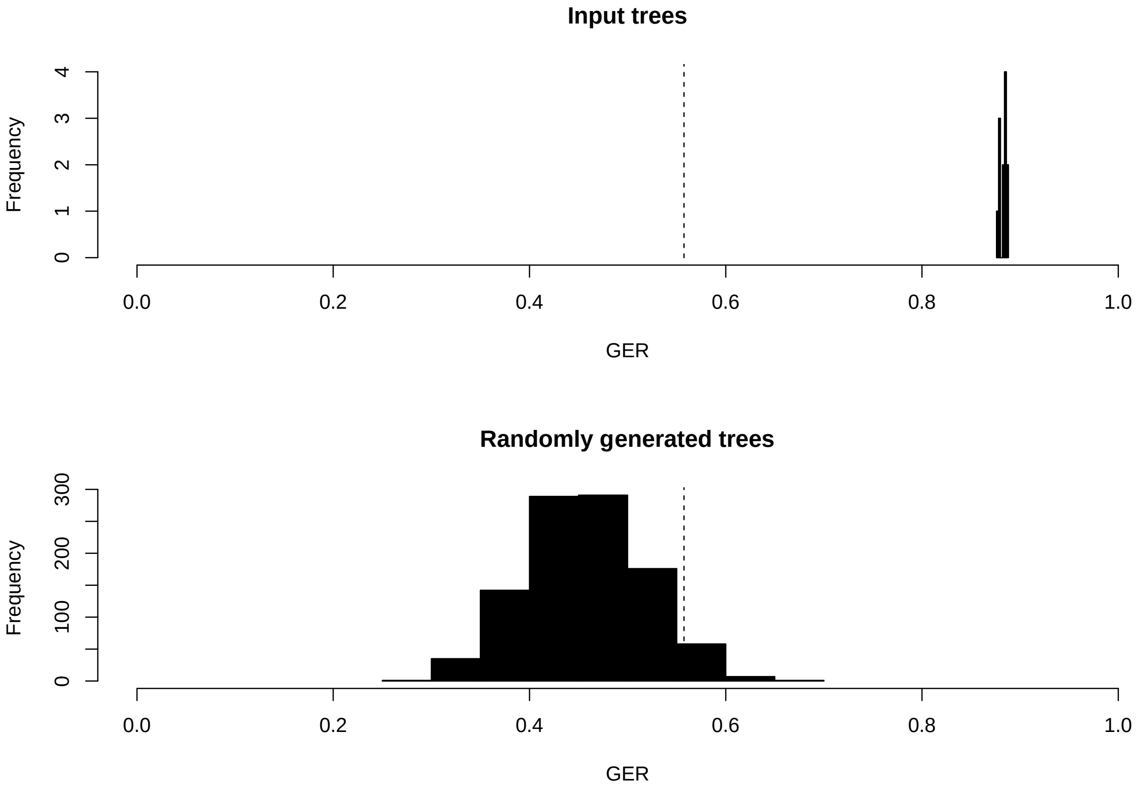

Supplementary Figure 3 | Stratigraphic congruence. Distribution of GER scores from most parsimonious trees compared to a sample of 1000 randomly generated trees using strap[7], showing the excellent stratigraphic congruence of the most parsimonious trees.

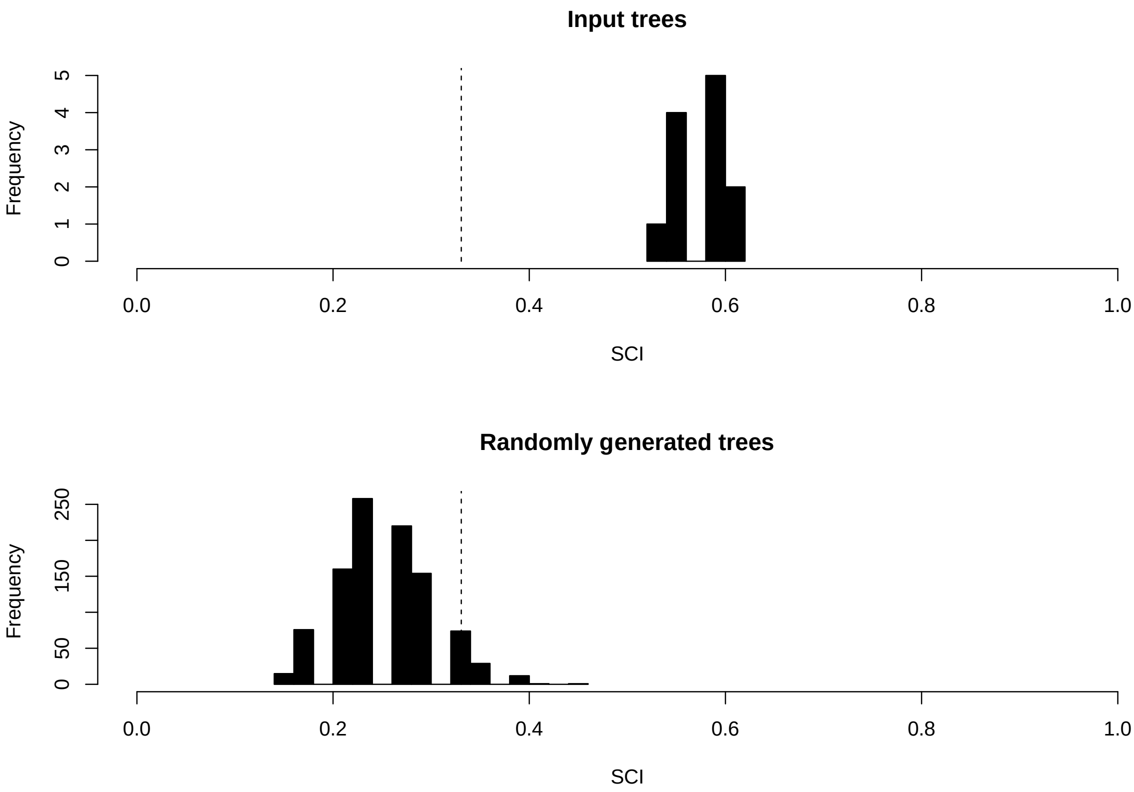

Supplementary Figure 4 | Stratigraphic congruence. Distribution of SCI scores from most parsimonious trees compared to a sample of 1000 randomly generated trees using strap[7], showing the excellent stratigraphic congruence of the most parsimonious trees.

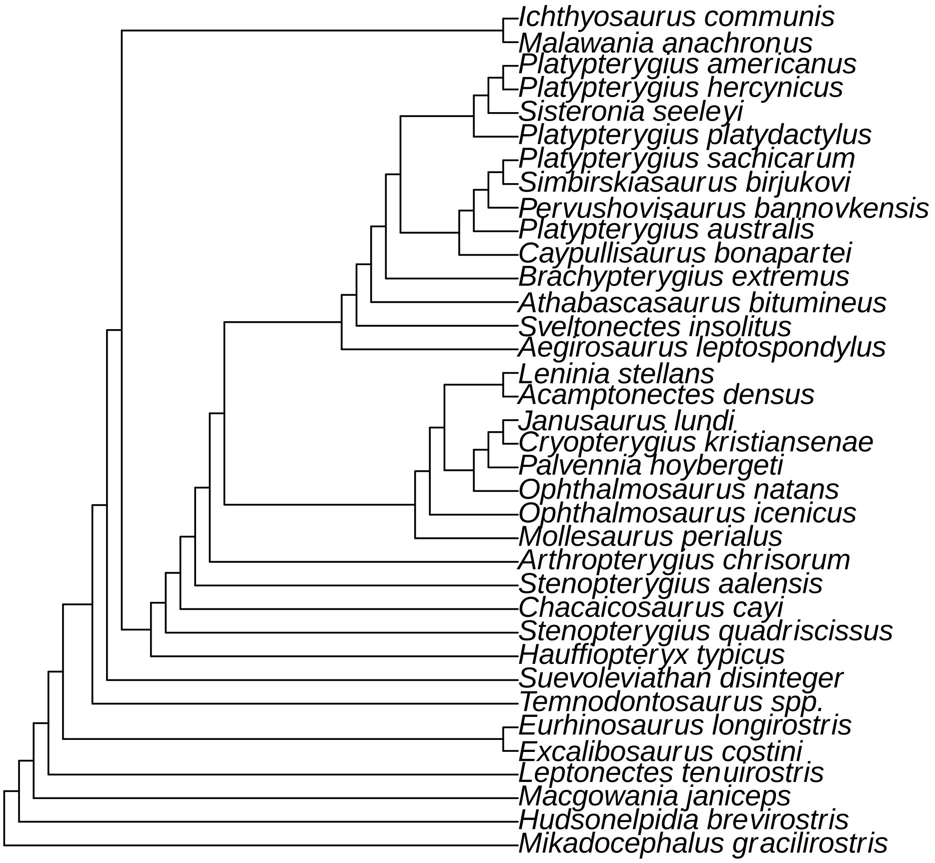

Supplementary Figure 5 | Most parsimonious tree from the implied weighting analysis. Length = 20.87381. This analysis recovered a single tree (length=20.87381). Although strongly similar, slight differences with the consensus tree from the equal weight analysis are recovered. *Temnodontosaurus* spp. is recovered as the sister taxon to *Suevoleviathan disinteger* + Thunnosauria instead of forming a clade with Leptonectidae. *Aegirosaurus leptospondylus*, *Sveltonectes insolitus*, *Athabascasaurus bitumineus* and *Brachypterygius extremus* are successive outgroups of more derived platypterygiines, which belong to two clades: (*Caypullisaurus bonapartei* + Platypterygiines with a paired narial aperture) on one side and (*Platypterygius platydactylus* + (*Sisteronia seeleyi* + '*Platypterygius*' *americanus* + '*Platypterygius*' *hercynicus*)) on the other side. This analysis supports a clade of Cretaceous ophthalmosaurines (*Acamptonectes densus* + *Leninia stellans*), as do a number of most parsimonious trees arising from the analysis with equal weights.

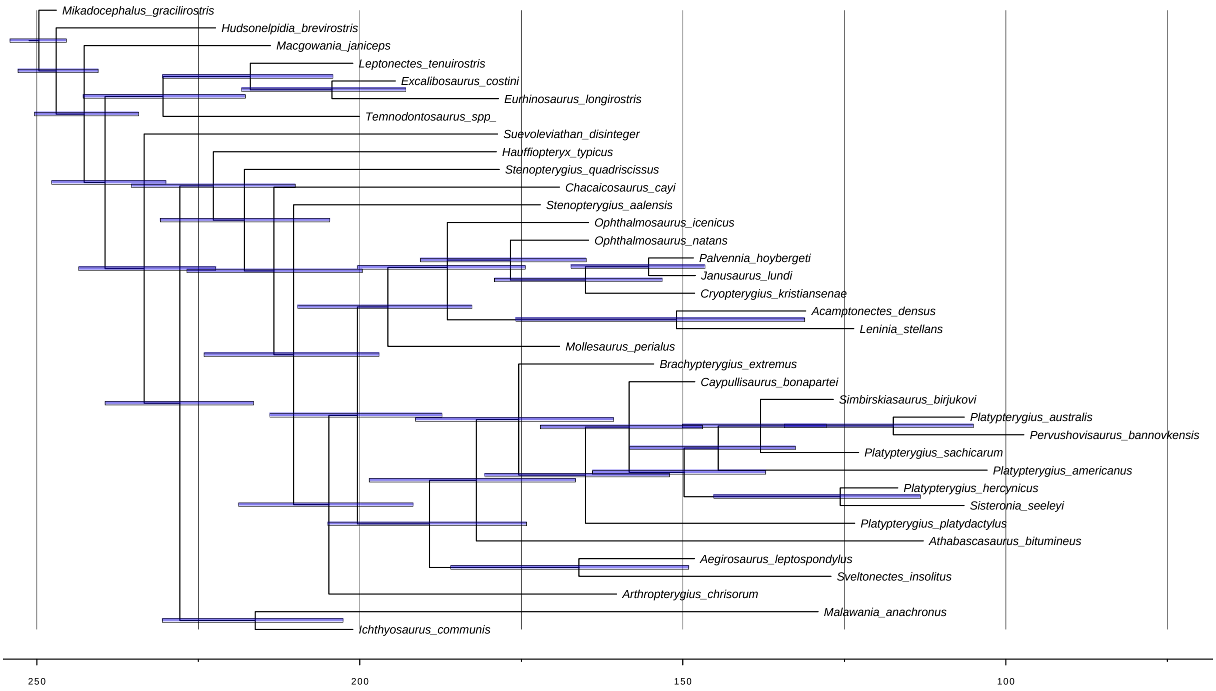

Supplementary Figure 6 | 95% confidence age intervals of clades. Computed for each node of the Bayesian inference of phylogeny, with the constrained typology. The topology of the majority rule consensus match that of the maximum parsimony tree with the best RCI and GER scores. Ages are expressed in millions years before present. It recognizes Leptonectidae with *Temnodontosaurus* as its sister group; a clade of younger leptonectids (*Excalibosaurus costini* + *Eurhinosaurus longirostris*); a clade of Cretaceous ophthalmosaurines (*Acamptonectes densus* + *Leninia stellans*); the two youngest taxa within the platypterygiine clade with a peculiar narial aperture, '*Platypterygius*' *australis* and *Pervushovisaurus bannovkensis* also form a clade.

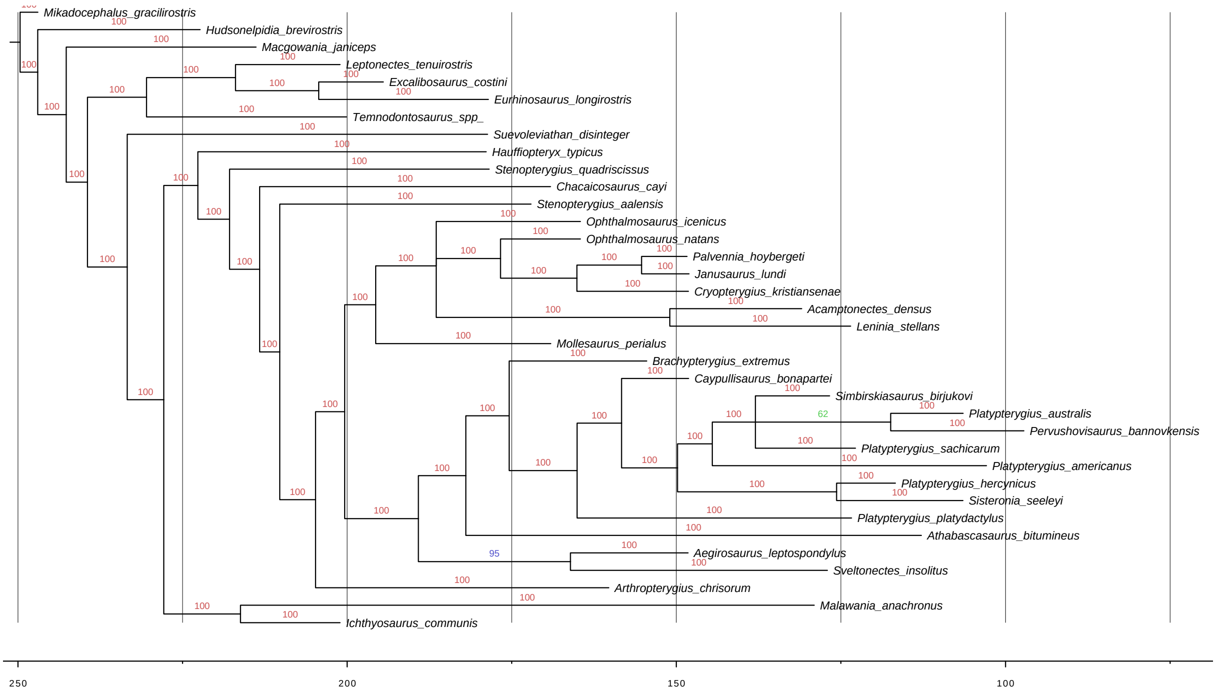

Supplementary Figure 7 | Posterior probabilities of each node. Computed on the Bayesian inference of phylogeny, with the constrained typology.

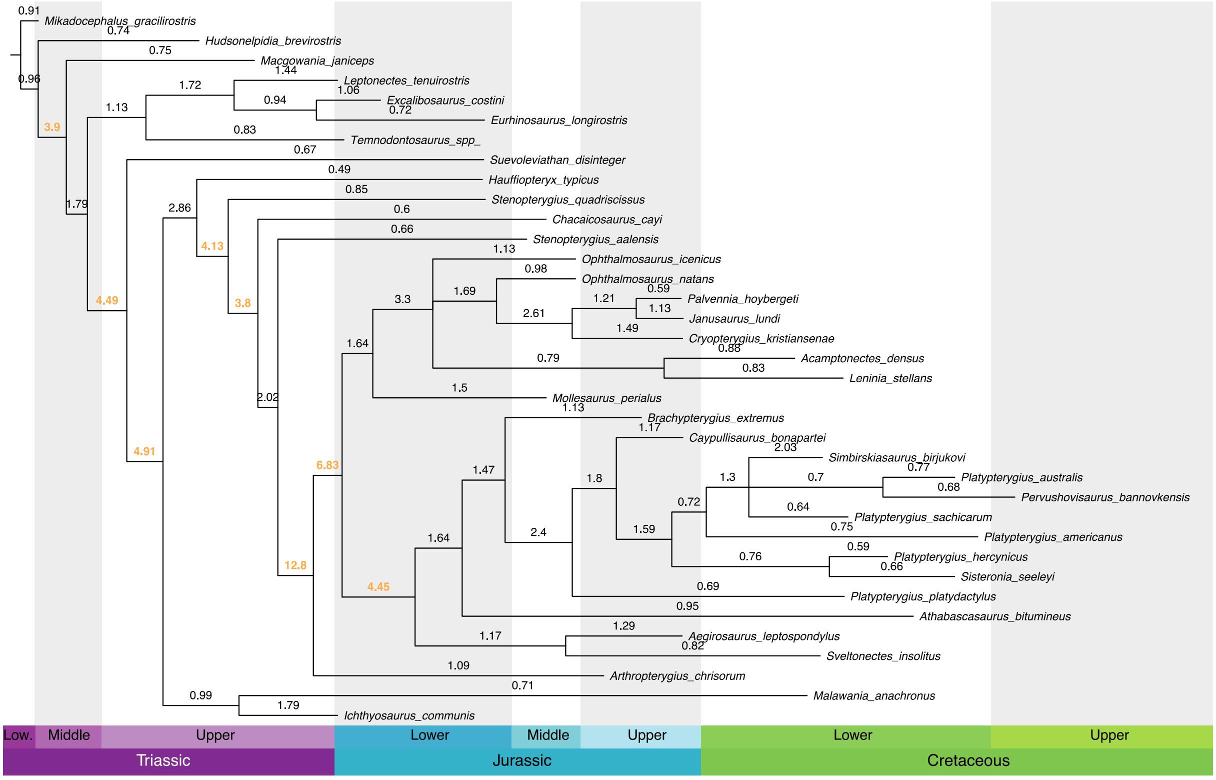

Supplementary Figure 8 | Evolutionary rates. Computed on the Bayesian inference of phylogeny, with the constrained typology. Exceptionally high rates are written in orange and are restricted to the early evolution of Parvipelvia, here entirely dragged into the Triassic.

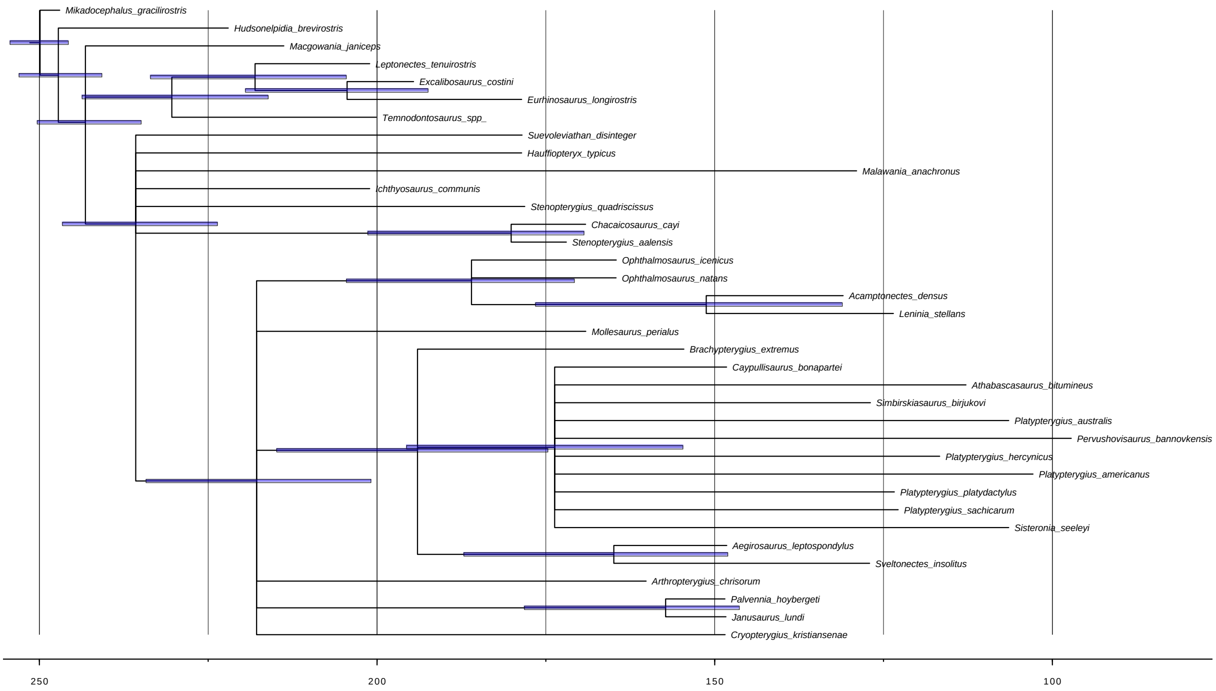

Supplementary Figure 9 | 95% confidence age intervals of clades. Computed for each node of the Bayesian inference of phylogeny, (unconstrained analysis)**.** Ages are expressed in millions years before present. The majority rule consensus is less well resolved but congruent with the results from the maximum parsimony analyses, with two exceptions: the Aalenian–Bajocian baracromians *Stenopterygius aalensis* and *Stenopterygius*/*Chacaicosaurus cayi* form a clade rather than a grade that is the sister group of Ophthalmosauridae and the Albian platypterygiine *Athabascasaurus bitumineus* is recovered as more derived than *Brachypterygius extremus*, *Aegirosaurus leptospondylus* and *Sveltonectes insolitus*, which form a polytomy at the base of Platypterygiinae. Particularly, the Bayesian inference supports the existence and further resolves the (*Temnodontosaurus* spp. + Leptonectidae) clade, the (*Ophthalmosaurus icenicus* + *Ophthalmosaurus natans* + Cretaceous ophthalmosaurines) clade and the base of the platypterygiine clade. Most importantly, despite its lower resolution, the Bayesian inference support the general shape of the parvipelvian tree that has emerged some years ago, with (i) the presence of three distinct clades of Cretaceous ichthyosaurs (early parvipelvians, ophthalmosaurines and platypterygiines), which (ii) diverged and rapidly evolved between the Late Triassic and the Middle Jurassic, (iii) relatively minor extinction events during or at the end of the Jurassic.

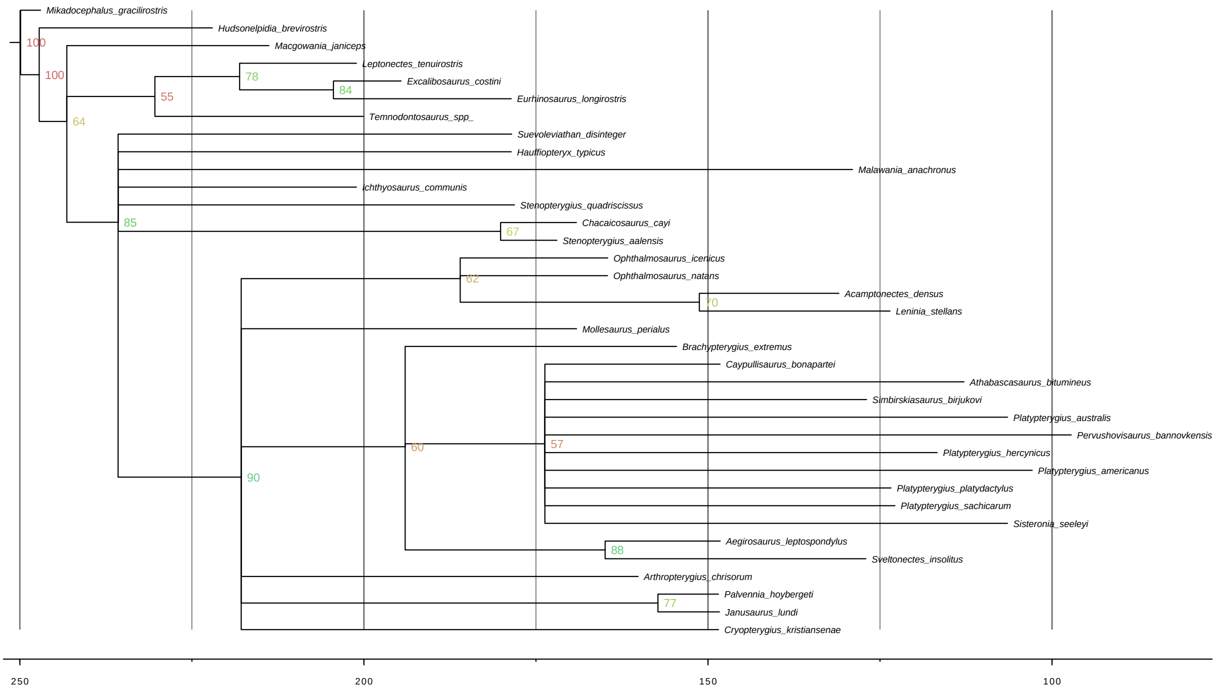

Supplementary Figure 10 | Posterior probabilities of each node. Computed on the Bayesian inference of phylogeny **(unconstrained analysis).**

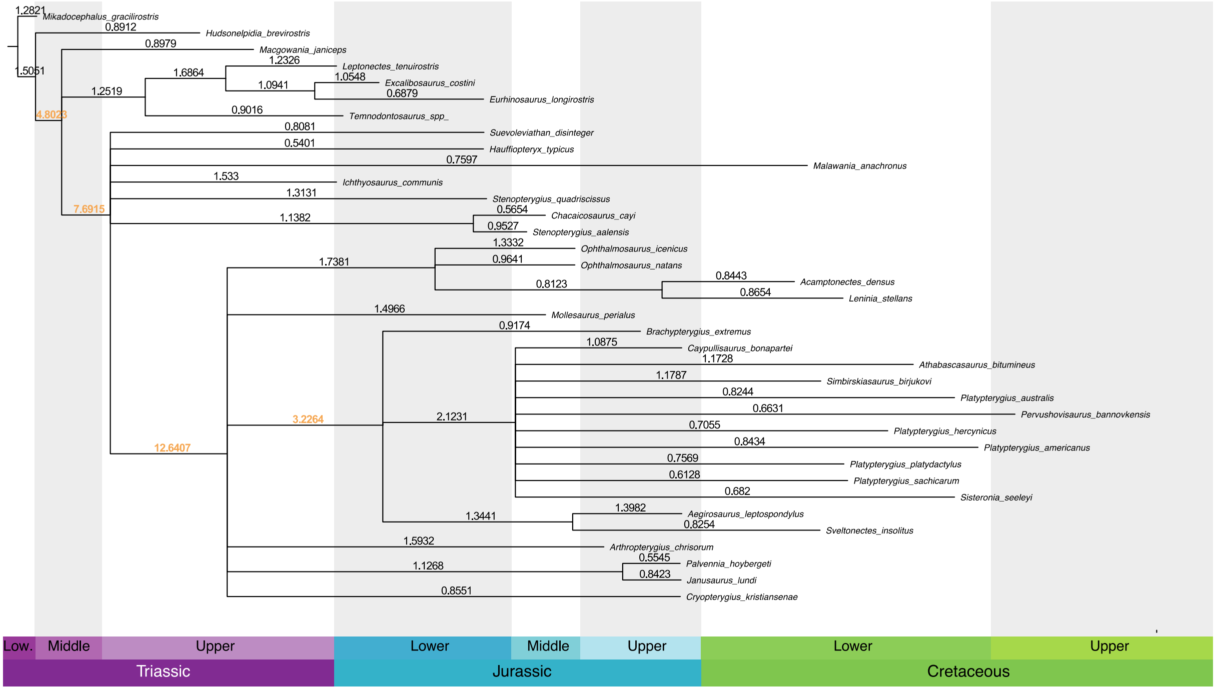

Supplementary Figure 11 | Evolutionary rates. Computed on the Bayesian inference of phylogeny (unconstrained analysis). Exceptionally high rates are written in orange and are restricted to the early evolution of Parvipelvia, here entirely dragged into the Triassic.

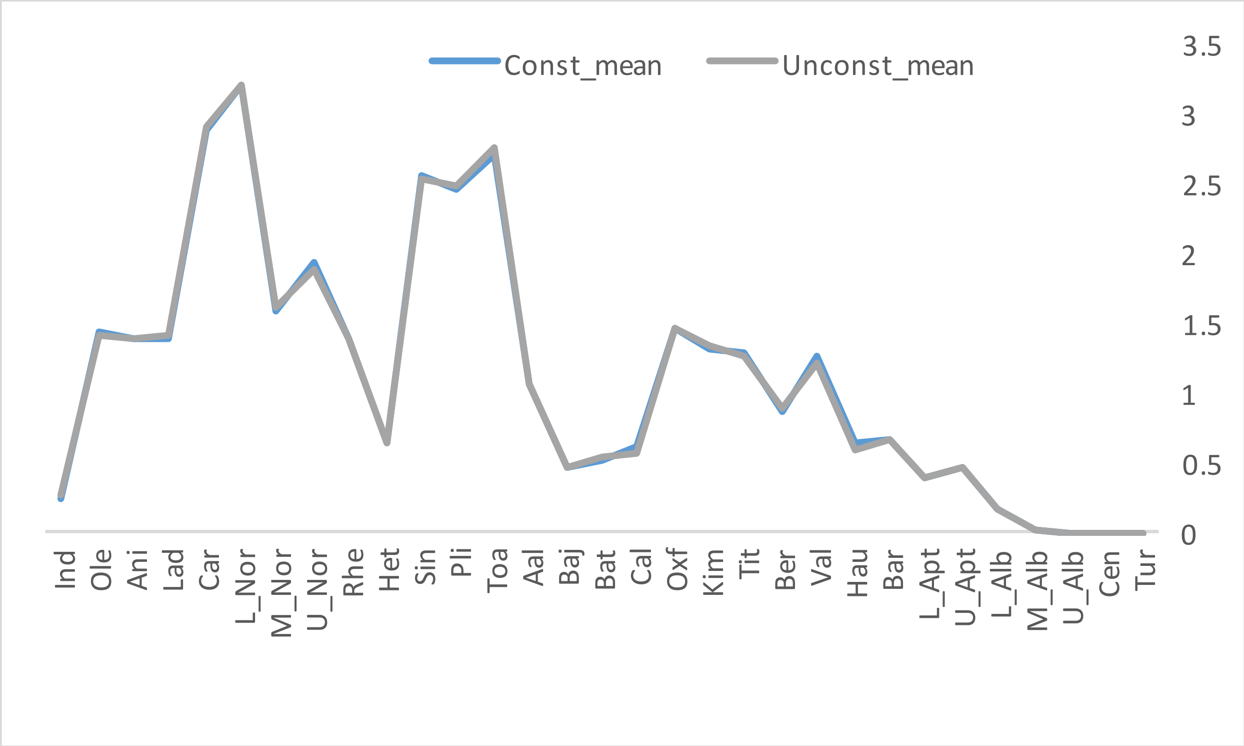

Supplementary Figure 12 | Congruence between the mean cladogenesis results. This graph shows that both the constrained and unconstrained analyses yield the same picture of parvipelvian evolutionary dynamics, even if the consensus tree arising from the unconstrained Bayesian analysis is less well-resolved than in the maximum parsimony analysis. Note the low values for the Cretaceous.

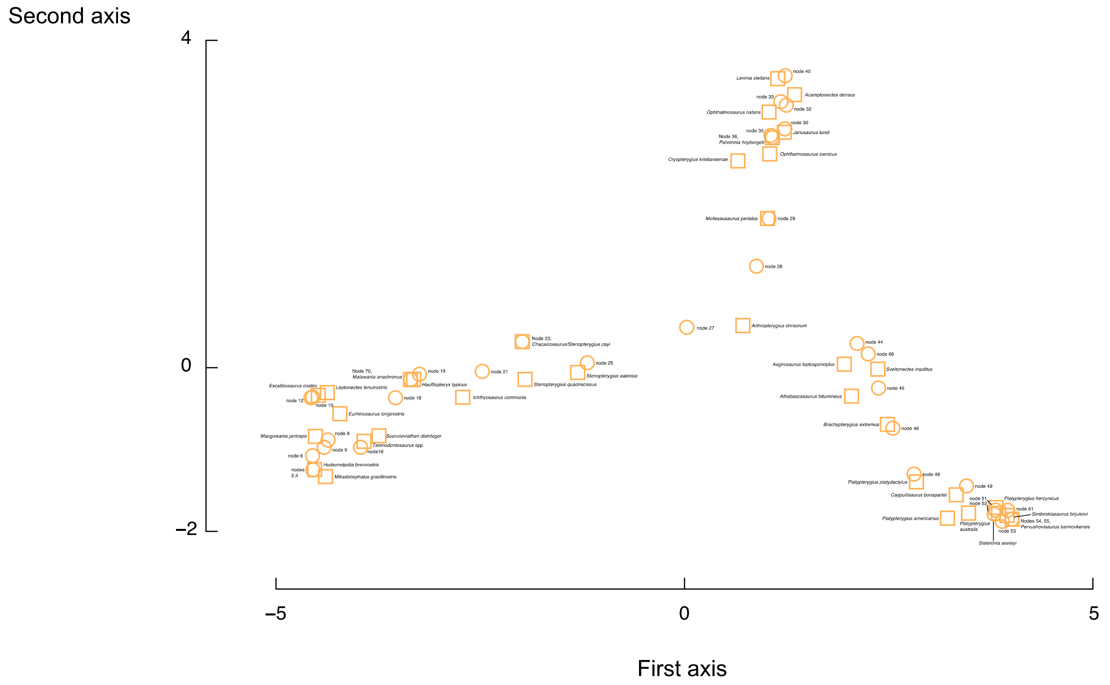

Supplementary Figure 13 | PCOA results. It shows the position of each taxon and each internal node relative to the first and second axes.

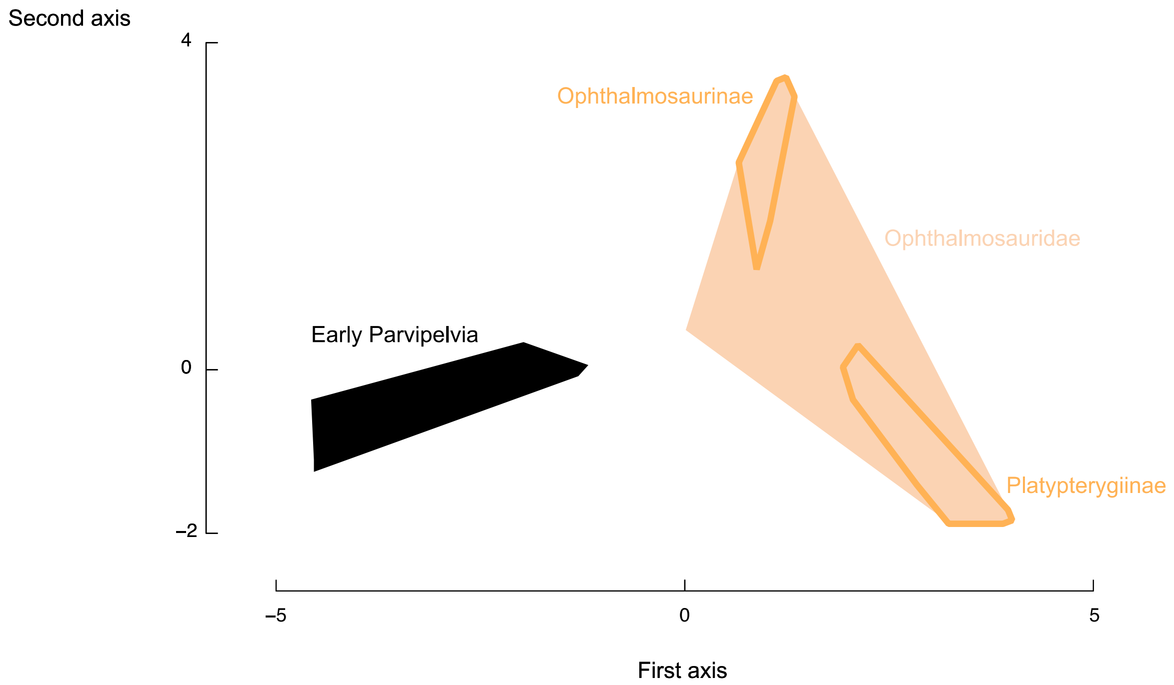

Supplementary Figure 14 | PCOA results. Note the clear morphological distinction between the three main clades of parvipelvian ichthyosaurs (Early Parvipelvians, Ophthalmosaurinae, Platypterygiinae). The left corner of the Ophthalmosauridae polygon is *Arthropterygius chrisorum*.

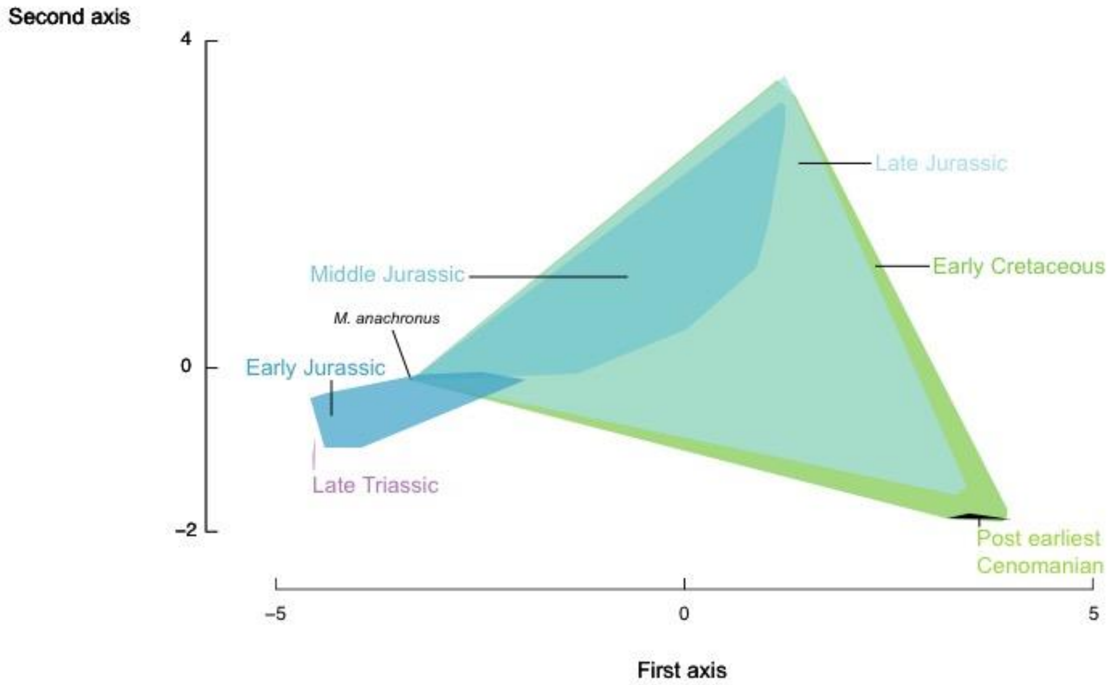

Supplementary Figure 15 | Morphospace occupation during the evolution of Parvipelvia. Note the extremely narrow areas for the Late Triassic and the post earliest Cenomanian, and the fact that the largest area is occupied during the Early Cretaceous.

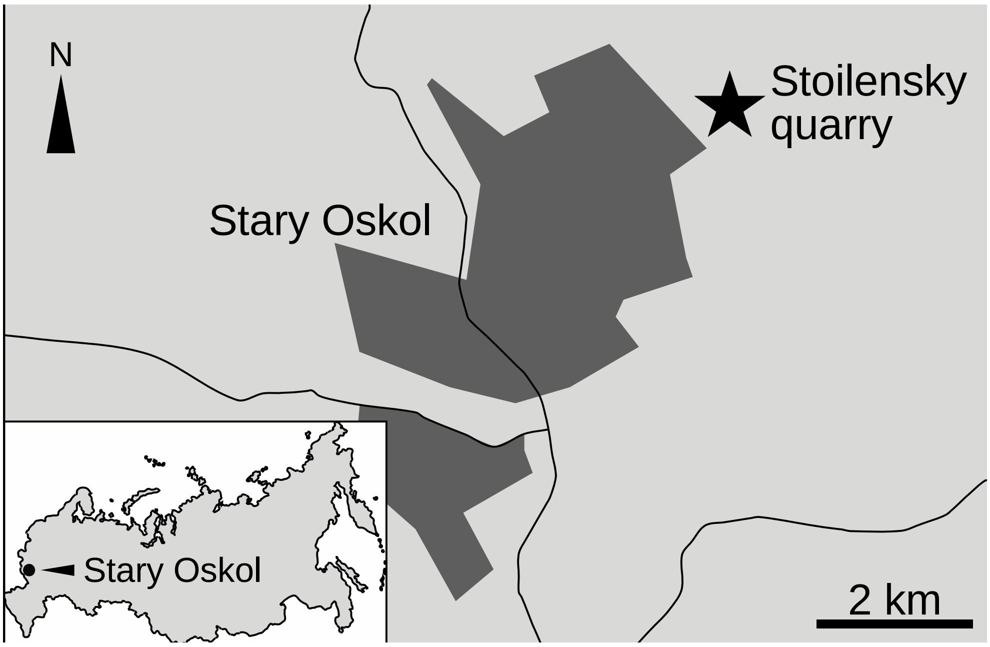

Supplementary Figure 16 | Localisation of the Stoilensky quarry. It is located northeastern to the town of Stary Oskol, in the Belgorod region, western-most Russia. The quarry was established in 1961 and exploits iron ore deposit of the 'Kursk Magnetic Anomaly'.

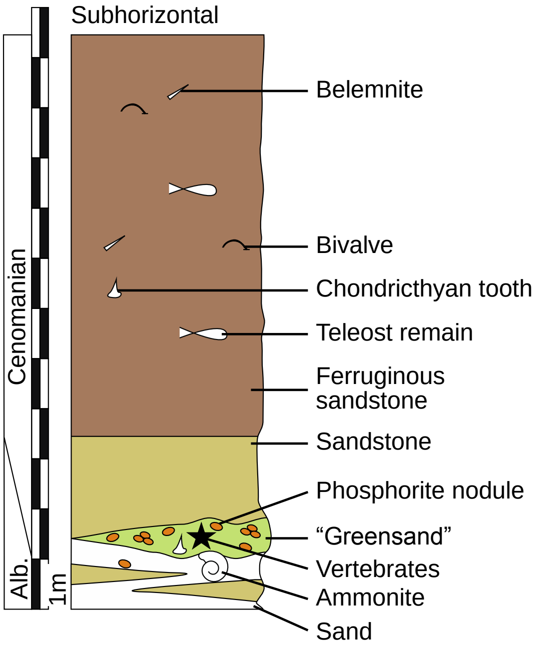

Supplementary Figure 17 | Stratigraphic log of the Stoilensky quarry. Data from Gabdullin[8]. "Greensand" refers to a 'greensand-like' phosphatic and glauconitic sandstone. This quarry section was described by Gabdullin[8]; a summary of the section is provided here. Lenticular intercalation of sands and sandstones forms the basal part of the section (1 m). The top of these sand/sandstone contains the late Albian ammonite *Mortoniceras inflatum*. Above, a lenticular, phosphatic, glauconitic, and fossiliferous sandstone (0–2.5 m) and its overlying two meters of clayey sandstone mark the Albian–Cenomanian boundary. Above, a thick layer of ferruginous sandstone (8 m) contains the following macrofauna according to Gabdullin[8]: chimaeriform (*Ischyodus* '*bifurcatus*' and shark teeth ('*Protosquales*' sp.), bivalves (*Neithea* sp.), and belemnites (*Praeactinocamax primus*, which ranges in the Russian platform from the *Mantelliceras mantelli* Zone (base of the Cenomanian) to the *Acanthoceras rhotomagense* Zone (early middle Cenomanian)[9,10]. The microfauna consists of late Cretaceous calcareous nannoplankton (*Broisonia matalosa*, Cenomanian–Turonian; *Manivitella redimiculata* and *Prediscosphaera cretacea*, Cenomanian–Maastrichtian[8]). The greensand-like rock thus deposited between the late Albian *Mortoniceras inflatum* Zone and the early–middle Cenomanian; it probably contains the Early–Late Cretaceous boundary and likely represents the onset of the early Cenomanian transgression. However, the precise position of the boundary is impossible to place. The Stoilensky fauna is thus considered here to occur at the Early–Late Cretaceous boundary, as hypothesized by Rozhdestvenskiy[11]. The 'greensand-like' layer and its fossils are therefore roughly contemporaneous with other similar deposits in France ('Gaize' formation)[12] and England (the Upper Greensand Formation and Cambridge Greensand Member)[13–15].

Taxon		Abundance in Stoilensky	
Platypterygius sp.	9		16%
cf. *Sisteronia seeleyi*	5		9%
Ophthalmosaurinae indet.	2		3.5%
4th ichthyosaur	14		25.5%
Polyptychodon	2		3.5%
Polycotylidae indet.	23		42%
	55		

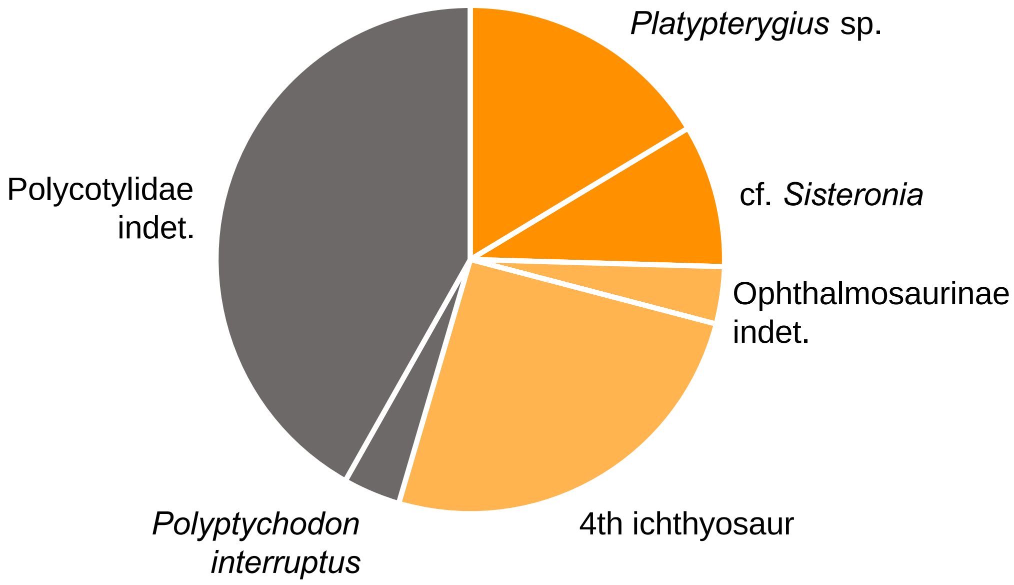

Supplementary Figure 18 | Marine reptile assemblage of the Stoilensky quarry. Based on the teeth housed at the Saratov State University (SSU). Plesiosaurs are coloured in grey, ichthyosaurs in orange (platypterygiine ichthyosaurs in dark orange; other ichthyosaurs in light orange). Ichthyosaurs dominate the assemblage, but a peculiarity of this ecosystem is the abundance of a yet indeterminate ichthyosaur and of polycotylid plesiosaurs[16]. As these abundance data rely on teeth, the relative proportions of these taxa should be taken with extreme caution because their tooth shedding frequencies is unknown, and likely pollute the signal.

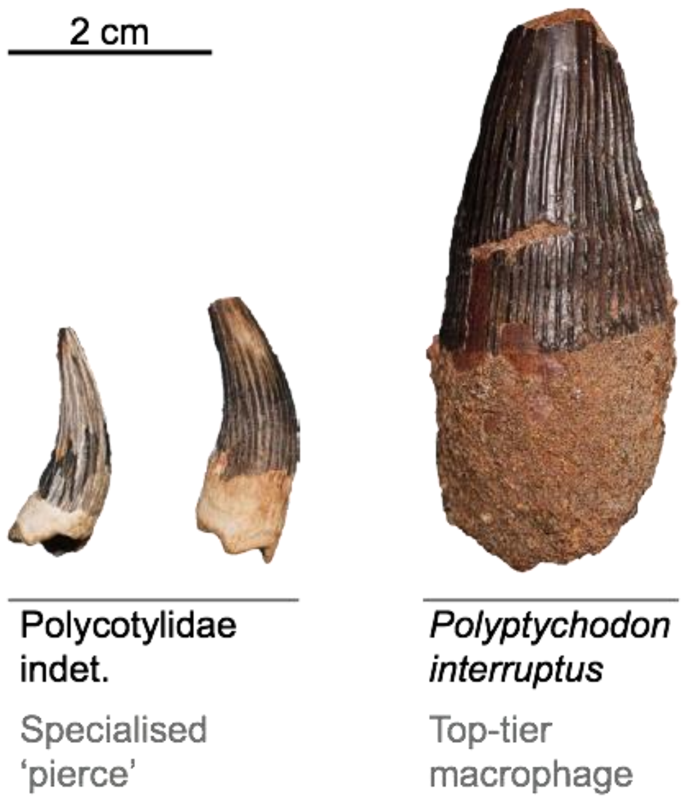

Supplementary Figure 19 | Selected plesiosaur teeth from the Stoilensky quarry. Specimens (GPV 2/ partim) illustrating the two feeding guilds colonised by plesiosaurs in this ecosystem.

SUPPLEMENTARY TABLES

Supplementary Table 1 | Names and ages of OTUs.

#	taxon_names	FAD Timescale 2014	LAD Timescale 2014	FAD Cret CSDB3	LAD Cret CSDB3	Strati/info	Range/Uncertainty
1	Mikadocephalus_gracilirostris	247.2	242	247.2	242	Topmost Anisian	U
2	Hudsonelpidia_brevirostris	227	216.4	227	216.4	Lower Norian (Norian substages ages from Husing et al.[17])	U
3	Macgowania_janiceps	216.4	211.4	216.4	211.4	Middle Norian (Norian top from Wotzlaw et al[18])	U
4	Leptonectes_tenuirostris	201.3	182.7	201.3	182.7	Lower Hettangian-Lower Pliensbachian	R
5	Excalibosaurus_costini	199.3	190.8	199.3	190.8	Sinemurian	U
6	Eurhinosaurus_longirostris	182.7	174.1	182.7	174.1	Lower Toarcian	R
7	Suevoleviathan_disinteger	182.7	174.1	182.7	174.1	Lower Toarcian	U
8	Temnodontosaurus_spp.	201.3	174.1	201.3	174.1	Upper Hettangian-Upper Toarcian	R
9	Hauffiopteryx_typicus	182.7	174.1	182.7	174.1	Lower Toarcian	R
10	Malawania_anachronus	132.9	125	132.13	124.55	upper Hauterivian-Barremian	U
11	Ichthyosaurus_communis	201.3	182.7	201.3	182.7	Hettangian-lower Pliensbachian	R
12	Stenopterygius_quadriscissus	182.7	174.1	182.7	174.1	Lower Toarcian	R
13	Chacaicosaurus_cayi	170.3	168.3	170.3	168.3	Lower Bajocian	U
14	Stenopterygius_aalensis	174.1	170.3	174.1	170.3	Lower Aalenian	U
15	Ophthalmosaurus_icenicus	166.1	139.8	166.1	141.6	Middle Callovian-Lower Tithonian + cf. *Ophthalmosaurus* from Berriasian Nettleton (*Primitivus* Zone)	R
16	Ophthalmosaurus_natans	166.1	157.3	166.1	157.3	upper Callovian–middle Oxfordian	R
17	Mollesaurus_perialus	170.3	168.3	170.3	168.3	Lower Bajocian	U
18	Acamptonectes_densus	132.9	129.4	136.44	130.2	Hauterivian	R
19	Leninia_stellans	125	121	123.75	123.61	Lower Aptian: *Deshayesites volgensis* = *D. forbesi* Zone in Europe	U
20	Brachypterygius_extremus	157.3	145	157.3	144.07	Middle Kimmeridgian-lower Tithonian	R
21	Arthropterygius_chrisorum	163.5	145	163.5	144.07	Oxfordian–Tithonian	R
22	Caypullisaurus_bonapartei	152.1	139.8	152.1	141.06	Lower Tithonian–Lower Berriasian	R
23	Aegirosaurus_leptospondylus	152.1	132.9	152.1	136.44	Lowermost Tithonian + lazarrus range from Fischer et al[19] CR: up to Upper Valanginian	R
24	Athabascasaurus_bitumineus	113	107.8	113.07	107.65	Lowermost Albian: Wabiskaw Member	U

25	Sveltonectes_insolitus	129.4	125	126.82	124.55	Upper Barremian	U
26	Simbirskiasaurus_birjukovi	129.4	125	130.2	126.82	Lower Barremian	U
27	Platypterygius_australis	107.8	100.5	107.65	97.13	Middle-Upper Albian	R
28	Pervushovisaurus_bannovkensis	100.5	93.9	96	94.8	Middle Cenomanian (see [20])	U
29	Platypterygius_hercynicus	121	100.5	121.25	98.14	Uppermost Aptian–Upper Albian (*Mortoniceras inflatum* Zone)	R
30	Platypterygius_americanus	105.5	93.9	101.83	95.05	Upper Albian- lower Cenomanian	R
31	Platypterygius_platydactylus	125	121	123.61	122.93	Lower Aptian:*Deshayesites deshayesi*	U
32	Platypterygius_sachicarum	125	121	124.55	121.25	Lower Aptian (Hampe[21])	U
33	Palvennia_hoybergeti	152.1	145	152.1	144.07	Tithonian	U
34	Cryopterygius_kristiansenae	152.1	145	152.1	144.07	Tithonian	U
35	Janusaurus_lundi	152.1	145	152.1	144.07	Tithonian	U
36	Sisteronia_seeleyi	107.8	93.9	107.65	95.05	Mid Albian (Marnes bleues Fm)–Lower Cenomanian (basal *mantelli* Zone: Glauconitic Marl Member)	R

Supplementary Table 2 | Names and ages of additional taxa.

Maiaspondylus_lindoei	113	107.8	113.07	107.65	Lower Albian	U
Cetharthrosaurus_walkeri	105.5	100.5	101.83	97.13	Uppermost Albian	U
Platypterygius_hauthali	129.4	125	130.2	124.55	Barremian	U
Platypterygius_ochevi	105.5	93.9	101.83	95.05	Upper Albian-lower Cenomanian	U
Nannopterygius_enthekiodon	157.3	145	157.3	144.07	Middle Kimmeridgian-lower Tithonian	U
Undorosaurus_gorodischensis	152.1	145	152.1	144.07	Tithonian	U
Undorosaurus_trautscholdi	152.1	145	152.1	144.07	Tithonian	U
Platypterygius_campylodon&sp	100.5	93.9	113.07	93	Cenomanian	R
Ophthalmosaurinae_indet2+ghost	121	100.5		97.13	Upper Albian (ghost is: Upper Aptian-Middle Albian)	U
Ophthalmosaurinae_indet1	170.3	168.3	170.3	168.3	Bajocian Druckenmiller & Maxwell[22]	U

Supplementary Table 3 | Phylogeny-adjusted diversity estimates.

	Supp_taxa	MPT1_basic	MPT1_eq	MPT2_basic	MPT2_eq	MPT3_basic	MPT3_eq	MPT4_basic	MPT4_eq	MPT5_basic	MPT5_eq	MPT6_basic	MPT6_eq	MPT7_basic	MPT7_eq	MPT8_basic	MPT8_eq	MPT9_basic	MPT9_eq	MPT10_basic	MPT10_eq	MPT11_basic	MPT11_eq	MPT12_basic	MPT12_eq
Tur	0	0	0	0	0	0	0	0	0	0	0	0	0	0	0	0	0	0	0	0	0	0	0	0	0
Cen	2	5	5	5	5	5	5	5	5	5	5	5	5	5	5	5	5	5	5	5	5	5	5	5	5
U_Alb	3	8	8	8	8	8	8	8	8	8	8	8	8	8	8	8	8	8	8	8	8	8	8	8	8
M_Alb	1	6	6	6	6	6	6	6	6	6	6	6	6	6	6	6	6	6	6	6	6	6	6	6	6
L_Alb	2	8	8	8	8	8	8	8	8	8	8	8	8	8	8	8	8	8	8	8	8	8	8	8	8
U_Apt	1	7	7	7	7	7	7	7	7	7	7	7	7	7	7	7	7	7	7	7	7	7	7	7	7
L_Apt	0	8	9	8	9	8	9	8	9	8	9	8	9	8	9	8	9	8	9	8	9	8	9	8	9
Bar	1	12	13	12	13	12	13	12	13	12	13	12	13	12	13	12	13	12	13	12	13	12	13	12	13
Hau	0	7	13	7	13	7	13	7	13	7	13	7	13	7	13	7	13	7	13	7	13	7	13	7	13
Val	0	7	14	7	14	7	14	7	14	7	14	7	14	7	14	8	14	7	14	8	14	8	14	8	14
Ber	0	9	13	9	13	9	13	9	13	9	13	9	13	9	13	10	13	9	13	10	13	10	13	10	13
Tit	3	17	19	17	19	17	19	17	19	17	19	17	19	17	19	18	19	17	19	18	19	18	19	18	19
Kim	1	10	15	11	15	10	15	11	15	11	15	10	15	11	15	11	16	10	15	10	16	11	16	10	16
Oxf	0	7	12	7	12	7	12	7	12	7	13	7	13	7	13	7	13	7	13	7	13	7	13	7	13
Cal	0	7	9	7	9	7	9	7	9	7	9	7	9	7	9	7	9	7	9	7	9	7	9	7	9
Bat	0	4	8	4	8	4	8	4	8	4	8	4	8	4	8	4	8	4	8	4	8	4	8	4	8
Baj	1	7	8	7	8	7	8	7	8	7	8	7	8	7	8	7	8	7	8	7	8	7	8	7	8
Aal	0	4	7	4	7	4	7	4	7	4	7	4	7	4	7	4	7	4	7	4	7	4	7	4	7
Toa	0	7	9	7	9	7	9	7	9	7	9	7	9	7	9	7	9	7	9	7	9	7	9	7	9
Pli	0	7	9	7	9	7	9	7	9	7	9	7	9	7	9	7	9	7	9	7	9	7	9	7	9
Sin	0	8	9	8	9	8	9	8	9	8	9	8	9	8	9	8	9	8	9	8	9	8	9	8	9
Het	0	7	8	7	8	8	8	8	8	7	8	7	8	8	8	7	8	8	8	7	8	8	8	8	8
Rhe	0	1	7	1	7	1	8	1	8	1	7	1	7	1	8	1	7	1	8	1	7	1	8	1	8
U_Nor	0	1	5	1	5	1	6	1	6	1	5	1	5	1	6	1	5	1	6	1	5	1	6	1	6
M_Nor	0	2	3	2	3	2	3	2	3	2	3	2	3	2	3	2	3	2	3	2	3	2	3	2	3
L_Nor	0	2	3	2	3	2	3	2	3	2	3	2	3	2	3	2	3	2	3	2	3	2	3	2	3
Car	0	1	2	1	2	1	2	1	2	1	2	1	2	1	2	1	2	1	2	1	2	1	2	1	2
Lad	0	1	2	1	2	1	2	1	2	1	2	1	2	1	2	1	2	1	2	1	2	1	2	1	2
Ani	0	2	2	2	2	2	2	2	2	2	2	2	2	2	2	2	2	2	2	2	2	2	2	2	2
Ole	0	2	2	2	2	2	2	2	2	2	2	2	2	2	2	2	2	2	2	2	2	2	2	2	2

Computed for each most parsimonious trees under both the basic and equal methods of branch length reconstruction. We applied the 'basic' and 'equal' methods to all most parsimonious trees and extracted the median phylogenetic diversity estimate as well as 95% confidence intervals using the R, using the following packages: `ape`[23], `strap`[7], and `paleotree` v2.3[24].

Supplementary Table 4 | Phylogeny-adjusted diversity estimates.

	median	low.95.quantile	high.95.quantile
Tur	0	0	0
Cen	3	3	3
U_Alb	5	5	5
M_Alb	5	5	5
L_Alb	6	6	6
U_Apt	6	6	6
L_Apt	8.5	8	9
Bar	11.5	11	12
Hau	10	7	13
Val	11	7	14
Ber	11.5	9	13
Tit	15.5	14	16
Kim	12	9	15
Oxf	9.5	7	13
Cal	8	7	9
Bat	6	4	8
Baj	6.5	6	7
Aal	5.5	4	7
Toa	8	7	9
Pli	8	7	9
Sin	8.5	8	9
Het	8	7	8
Rhe	4	1	8
U_Nor	3	1	6
M_Nor	2.5	2	3
L_Nor	2.5	2	3
Car	1.5	1	2
Lad	1.5	1	2
Ani	2	2	2
Ole	2	2	2

Median and 95% confidence interval values.

Supplementary Table 5 | Sum of variances of first 46 axes of pcoa for each bin.

	basic	basic_05	basic_95	eq	eq_05	eq_95
Tur	0	0	0	0	0	0
Cen	2.922526442	2.433795789	5.759348039	2.922526442	2.433795789	5.759348039
U_Alb	4.972452414	4.174608598	9.690037237	4.972452414	4.174608598	9.690037237
M_Alb	4.972452414	4.170352073	9.672162499	4.972452414	4.170352073	9.672162499
L_Alb	5.787084788	4.918089302	11.43161404	5.787084788	4.918089302	11.43161404
U_Apt	5.787084788	4.928377068	11.41592151	5.787084788	4.928377068	11.41592151
L_Apt	7.372678979	6.604640129	14.64344293	8.423765351	7.487005801	16.62169364
Bar	14.00410551	12.2718051	27.6365967	11.1280948	9.965607926	22.17570461
Hau	9.871812203	8.855907151	19.84021066	12.00662083	10.79103206	24.10466287
Val	5.869844517	5.408472518	12.06602566	15.75275897	14.007219	30.7439694
Ber	7.549072758	6.979618952	15.81268942	13.45295606	12.1474114	27.36095578
Tit	12.8054886	12.01315218	26.82981355	15.06688999	14.07941177	31.46060219
Kim	9.740763644	9.247244808	21.06243851	13.55150102	12.69070022	28.53591377
Oxf	6.631850487	6.439518403	14.49675107	11.60900305	11.0597531	24.64986837
Cal	6.57178483	6.596172187	14.59816062	7.575887334	7.23215267	16.37365647
Bat	4.212808214	4.183454853	9.885335355	8.112146077	7.926033741	18.07485897
Baj	4.962313983	5.020580063	11.74765423	5.519647067	5.543728138	12.77534203
Aal	3.845336481	4.029228215	9.01881203	5.963973371	6.133401575	14.14164499
Toa	7.471570395	6.916494727	14.97679593	8.367676359	7.961710804	17.13718265
Pli	7.436163378	6.753145153	14.62635706	9.021056128	8.237774735	17.92315547
Sin	7.893493319	7.128897267	15.08777559	9.443632849	8.56708154	18.39679264
Het	11.25769498	10.12574734	21.62564991	8.943907052	8.133285869	17.23591065
Rhe	5.532269856	4.89615084	10.21039276	8.499208471	7.69780416	16.32490838
U_Nor	NA	NA	NA	7.342866032	6.545221173	14.20584084
M_Nor	1.877058022	1.745064101	3.604100587	3.792381198	3.394526035	7.0286689
L_Nor	2.117533846	1.780630902	3.558150711	3.994591868	3.541769735	7.16302078
Car	NA	NA	NA	2.117533846	1.782596749	3.606101802
Lad	NA	NA	NA	3.17009254	2.628764709	5.202015955
Ani	2.186923003	1.831439887	3.654618086	2.186923003	1.831439887	3.654618086
Ole	2.186923003	1.822753028	3.651363229	2.186923003	1.822753028	3.651363229

We used both the basic and equal methods of branch length reconstruction. These axes explain 95.03+% of the variance explained. 95% confidence intervals achieved by bootstrapping the data 10000 times.

Supplementary Table 6 | Weighted mean pairwise phenetic dissimilarity.

	weighted_mean	weighted_mean_0.05	weighted_mean_0.95
Cen_Tur	0.157894737	0.066666667	0.217391304
Alb	0.142553191	0.096774194	0.196721311
Apt	0.263888889	0.121212121	0.487179487
Hau_Bar	0.365591398	0.32	0.421052632
Ber_Val	0.261904762	0.261904762	0.261904762
Kim_Tit	0.273709484	0.248979592	0.297802198
Cal_Oxf	0.217391304	0.160839161	0.310344828
Aal_Baj_Bat	0.233333333	0.076923077	0.523809524
Pli_Toa	0.237997957	0.2113127	0.264880952
Het_Sin	0.240896359	0.166153846	0.310606061
L_Tr	0.109090909	0.068965517	0.153846154

95% confidence intervals achieved by bootstrapping the data 10000 times.

Supplementary Table 7 | Mean and median cladogenesis rates for each bin.

	mean	median	med.05%	med.95%	mean-stdev	mean+stdev
Tur	0	0	0	0	0	0
Cen	0	0	0	0	0	0
U_Alb	0	0	0	0	0	0
M_Alb	0	0	0	0	0	0
L_Alb	0	0	0	0	0	0
U_Apt	0	0	0	0	0	0
L_Apt	0.5	0.5	0	1	-0.010753918	1.010753918
Bar	0	0	0	0	0	0
Hau	2.5	2.5	0	5	-0.053769592	5.053769592
Val	1.833333333	2	1	3	0.593885116	3.072781551
Ber	1	1	0	2	-0.021507837	2.021507837
Tit	0.833333333	0.5	0	1	-0.083498009	1.750164676
Kim	3.75	3.5	2.5	4.5	2.490255374	5.009744626
Oxf	3.416666667	3	3	3	2.913056511	3.920276822
Cal	0.666666667	0.5	0	1	-0.094720321	1.428053654
Bat	3	3	3	3	3	3
Baj	0.5	0.5	0	1	-0.010753918	1.010753918
Aal	3	3	3	3	3	3
Toa	2	2	2	2	2	2
Pli	1.5	1.5	1	2	0.989246082	2.010753918
Sin	0.5	0.5	0	1	-0.010753918	1.010753918
Het	0.5	0.5	0	1	-0.010753918	1.010753918
Rhe	4.25	4	2	6	1.923405175	6.576594825
U_Nor	1.75	1.5	0	3	-0.073756277	3.573756277
M_Nor	0.5	0.5	0	1	-0.010753918	1.010753918
L_Nor	1	1	1	1	1	1
Car	0.5	0.5	0	1	-0.010753918	1.010753918
Lad	0.5	0.5	0	1	-0.010753918	1.010753918
Ani	0	0	0	0	0	0
Ole	1	1	1	1	1	1
Ind	0	0	0	0	0	0

Computed using the results from the maximum parsimony analysis.

Supplementary Table 8 | Mean and median cladogenesis rates.

	Const_mean	Const_median	Const_5%	Const_95%	Unconst_mean	Unconst_median	Unconst_5%	Unconst_95%
Tur	0	0	0	0	0	0	0	0
Cen	0	0	0	0	0	0	0	0
U_Alb	0	0	0	0	0.000333333	0	0	0
M_Alb	0.017666667	0	0	0	0.022666667	0	0	0
L_Alb	0.164333333	0	0	1	0.166333333	0	0	1
U_Apt	0.464	0	0	2	0.476	0	0	2
L_Apt	0.388666667	0	0	1	0.392333333	0	0	1
Bar	0.674	1	0	2	0.654333333	1	0	2
Hau	0.632666667	0	0	2	0.588	0	0	2
Val	1.256333333	1	0	3	1.218	1	0	3
Ber	0.875333333	1	0	2	0.887	1	0	2
Tit	1.297666667	1	0	3	1.273	1	0	3
Kim	1.303333333	1	0	3	1.346666667	1	0	3
Oxf	1.456333333	1	0	3	1.463666667	1	0	3
Cal	0.615	0	0	2	0.570333333	0	0	2
Bat	0.525333333	0	0	2	0.534333333	0	0	2
Baj	0.467333333	0	0	2	0.473	0	0	2
Aal	1.065333333	1	0	3	1.073666667	1	0	3
Toa	2.700333333	3	0	5	2.753	3	1	5
Pli	2.456333333	2	0	5	2.483333333	2	0	5
Sin	2.554666667	2	0	5	2.522	2	0	5
Het	0.639666667	0	0	2	0.645333333	0	0	2
Rhe	1.385	1	0	3	1.381333333	1	0	3
U_Nor	1.921333333	2	0	4	1.891666667	2	0	4
M_Nor	1.596333333	1.5	0	4	1.606333333	1	0	4
L_Nor	3.198333333	3	1	6	3.2	3	1	6
Car	2.885666667	3	1	5	2.915333333	3	1	5
Lad	1.391666667	1	0	3	1.408333333	1	0	3
Ani	1.385333333	1	0	3	1.389	1	0	3
Ole	1.428666667	1	0	3	1.402333333	1	0	3
Ind	0.253333333	0	0	1	0.262333333	0	0	1

Using the results (1000 posterior trees randomly sampled in each run, total of 3000 trees for each analysis) from the constrained and unconstrained Bayesian inference of phylogeny.

Supplementary Table 9 | Evolutionary rates.

	Mean_const	Const_05	Const_95	Mean_unconst	Unconst_05	Unconst_95
Tur	NA	NA	NA	NA	NA	NA
Cen	0.682754	4.00E-06	1.528389	0.663055	4.00E-06	1.522824
U_Alb	0.716906	0.1045845	1.4496345	0.753216	0.053324	1.6652415
M_Alb	0.7159955	0.0522965	1.56776675	0.7532165	0.0266625	1.75057925
L_Alb	0.7633372	0.1225176	1.5704762	0.8371392	0.072099	1.8795408
U_Apt	0.729599286	0.087512857	1.621734857	0.815193167	0.060082667	1.8971625
L_Apt	0.725390375	0.0955945	1.6354485	0.791801556	0.040074111	1.876763778
Bar	0.843733917	0.099521417	1.940768583	0.824172	0.060258	1.923865833
Hau	0.886361909	0.108567364	2.001046091	0.825717769	0.055622846	1.921007308
Val	0.92093425	0.099520167	2.174381167	0.825717769	0.055622846	1.921007308
Ber	0.8403883	0.119424	1.9002227	0.825717769	0.055622846	1.921007308
Tit	0.994929333	0.076591867	2.479888667	0.859543722	0.040172389	2.085857889
Kim	1.043872538	0.093116231	2.518166308	0.860652667	0.040163222	2.086034111
Oxf	1.133720308	0.116077308	2.682957462	0.934161389	0.060416222	2.186590889
Cal	1.2733842	0.100600533	3.019271	0.955613	0.0543747	2.25805155
Bat	1.252741818	0.108664091	2.817428909	0.959618053	0.050222053	2.272890895
Baj	1.254887167	0.115080167	2.797144417	0.966413762	0.045439143	2.285317238
Aal	1.208743538	0.112778154	2.680774231	0.965792136	0.043373773	2.3035995
Toa	1.163058	0.10626355	2.76963225	1.073937889	0.0560715	2.499881444
Pli	1.342159313	0.103623063	3.236045563	1.150050471	0.059369765	2.662828294
Sin	1.364899077	0.127535615	3.351071538	1.295151875	0.063080313	3.089749625
Het	1.588552733	0.1136834	4.2091656	1.250321938	0.072162875	2.940938188
Rhe	2.098113467	0.113683533	5.7586372	1.241133412	0.067918059	2.932373412
U_Nor	1.98252875	0.1099825	5.470389833	1.290443133	0.072925	3.028871933
M_Nor	1.240538583	0.111171417	3.315729333	1.265909688	0.068367313	2.969748875
L_Nor	1.554817077	0.084344769	4.341444692	1.866992474	0.057573632	4.764909789
Car	1.9090909	0.0601348	5.3759116	2.465652385	0.056103	6.759598692
Lad	1.779416	0.0369234	5.3052644	2.3741406	1.60E-06	6.8868954
Ani	1.508123667	0.030769833	5.029193667	2.617428571	1.43E-06	8.506329143
Ole	0.934651	1.00E-06	3.9287715	1.393607	1.00E-06	5.903716
Ind	NA	NA	NA	NA	NA	NA

Mean values and 95% confidence interval. These are the morphological clock rates, for each bin, arising from the constrained and unconstrained Bayesian inference of phylogeny.

Supplementary Table 10 | Extinction and turnover rates per bin.

	Extinction	Per_lineage_extinction	Turnover_est
Tur	0	NA	0
Cen	5	100.00%	5
U_Alb	4	50.00%	4
M_Alb	0	0.00%	0
L_Alb	2	25.00%	2
U_Apt	0	0.00%	0
L_Apt	3	35.29%	3.5
Bar	4	32.00%	4
Hau	1	10.00%	3.5
Val	1	9.09%	2.833333333
Ber	2	17.39%	3
Tit	8	45.71%	8.833333333
Kim	0	0.00%	3.75
Oxf	1	10.53%	4.416666667
Cal	0	0.00%	0.666666667
Bat	0	0.00%	3
Baj	3	40.00%	3.5
Aal	1	18.18%	4
Toa	5	62.50%	7
Pli	2	25.00%	3.5
Sin	1	11.76%	1.5
Het	0	0.00%	0.5
Rhe	0	0.00%	4.25
U_Nor	0	0.00%	1.75
M_Nor	1	40.00%	1.5
L_Nor	1	40.00%	2
Car	0	0.00%	0.5
Lad	0	0.00%	0.5
Ani	1	50.00%	1
Ole	0	0.00%	1

Values claculated at the top boundary of each bin. The relative extinction (per lineage extinction) rate is the percentage of the total diversity estimate going extinct during that bin. The estimated turnover rate (turnover_est) is the sum of the mean cladogenesis rate and the extinction rate.

Supplementary Table 11 | Diversity dynamics for the Albian–Cenomanian interval.

	Late Albian	Basal Cenomanian	Early Cenomanian	Mid Cenomanian	Late Cenomanian
Lineages	7	5	3-4	2-3	1
Extinction	3	2	1	2	1
Per lineage extinction	0.42	0.4	0.25	0.6	1

Supplementary Table 12 | Ecological data for selected Ophthalmosauridae.

	Data sources	Tooth size	Crown shape	Crown relative size	Symphysis	Snout depth	Sclerotic aperture	Wear
**Ophthalmosaurus_icenicus**	HM V1129 [25]	37.3	1.66	0.16	53.05	0.54	71.1	NA
**Ophthalmosaurus_natans**	[26,27]; CM 603	29	2.35	0.14	NA	0.54	100	NA
**Mollesaurus_perialus**	[28]	20	NA	0.08	NA	NA	70.6	NA
**Acamptonectes_densus**	GLAHM 132855 (*=SNHM1284-R)	NA	2.66	0.17	NA	0.44*	NA	NA
**Brachypterygius_extremus**	[25], CAMSMJ68516	53.4	1.54	0.26	NA	0.8	NA	NA
**Aegirosaurus_leptospondylus**	[29], (*=RGHP LA 1)	26*	1.4*	NA	NA	0.62	32.76	1.5*
**Sveltonectes_insolitus**	IRSNB R129	19	2.86	0.12	50.6	0.47	34.4	1.2
**Simbirskiasaurus_birjukovi**	YKM 65119	NA	1.91	0.26	NA	NA	NA	2
**Platypterygius_australis**	[30–32]	55	1.65	0.31	40	0.48	31.5	NA
**Pervushovisaurus_bannovkensis**	SSU 104a/24	60	1.49	NA	NA	NA	NA	NA
**Platypterygius_hercynicus**	[33], MNHN2010	50	1.51	0.22	0.51	NA	NA	NA
**Platypterygius_americanus**	UW 2421 ([34] and photographs)	NA	1.63	0.23	50.8	0.43	51	NA
**Platypterygius_sachicarum**	DON-19671 ([35] and photographs)	40	1.53	NA	NA	0.49	NA	2.3
**Sisteronia_seeleyi**	CAMSM TN1779	33.8	1.75	0.2	NA	NA	NA	1.7
**Platypterygius_sp._Europe**	RGHP PR1	55	1.91	NA	NA	NA	NA	2.4

The values are rounded to the nearest % for visual purposes; the precise values can be found in "Supplementary data 7 ecodata.txt".

Supplementary Table 13 | Cretaceous ichthyosaur from Russia studied here.

**Specimen**	**Material**	**Assignation**	**Locality**
NHMUK 33245	4 teeth (Kiprijanoff collection)	‘*Platypterygius*’ sp.	Kursk
NHMUK 33245	Tooth (Kiprijanoff collection)	cf. *Sisteronia*	Kursk
SSU 14/8 137/176	Interclavicle	Ichthyosauria indet.	Stoilensky quarry
SSU 14/8 137/177	Interclavicle	Ichthyosauria indet.	Stoilensky quarry
SSU 14/5 137/174	Centrum	Ichthyosauria indet.	Stoilensky quarry
SSU 14/6 137/152,54	Centra	Ichthyosauria indet.	Stoilensky quarry
SSU GPV 2/xx partim	9 teeth	‘*Platypterygius*’ sp.	Stoilensky quarry
SSU GPV 2/ partim	5 teeth	Cf. *Sisteronia*	Stoilensky quarry
SSU GPV 2/ partim	2 teeth	Cf. Ophthalmosaurinae	Stoilensky quarry
SSU GPV 2/ partim	14 teeth	Ichthyosauria indet.	Stoilensky quarry
SSU 14/37	Left humerus	Cf. Ophthalmosaurinae	Stoilensky quarry
SSU 14/37 837/46	Left humerus	Cf. Ophthalmosaurinae	Late Albian of the Krasny Tekstilshik locality (Saratov region)
SSU 14/44 137/122	Left femur	‘*Platypterygius*’ sp.	Cenomanian of the Pudovkino locality (Saratov region), reworked in a Turonian deposit

All specimens are from the Early-Late Cretaceous boundary.

Supplementary Table 14 | Important ichthyosaurs from the British Cenomanian.

Specimen	Material	Assignation	Locality
CAMSM B20643	Tooth	Platypterygiinae indet. (holotype of *I. angustidens*)	Hunstanton
CAMSM B20644	Tooth	*P. campylodon* (syntype, Carter's series)	Cambridge area
CAMSM B20645	Tooth	*P. campylodon* (syntype, Carter's series)	Cambridge area
CAMSM B20646	Tooth	*P. campylodon* (syntype, Carter's series)	Cambridge area
CAMSM B20647	Tooth	*P. campylodon* (syntype, Carter's series)	Cambridge area
CAMSM B20648	Tooth	*P. campylodon* (syntype, Carter's series)	Cambridge area
CAMSM B20649	Tooth	*P. campylodon* (syntype, Carter's series)	Cambridge area
CAMSM B20650	Tooth	*P. campylodon* (syntype, Carter's series)	Cambridge area
CAMSM B20651	Tooth	*P. campylodon* (syntype, Carter's series)	Cambridge area
CAMSM B20652	Tooth	*P. campylodon* (syntype, Carter's series)	Cambridge area
CAMSM B20653	Tooth	*P. campylodon* (syntype, Carter's series)	Cambridge area
CAMSM B20654	Tooth	*P. campylodon* (syntype, Carter's series)	Cambridge area
CAMSM B20655	Tooth	*P. campylodon* (syntype, Carter's series)	Cambridge area
CAMSM B20656	Tooth	*P. campylodon* (syntype, Carter's series)	Cambridge area
CAMSM B20657	Tooth	*P. campylodon* (syntype, Carter's series)	Cambridge area
CAMSM	Tooth	*P. campylodon* (syntype,	Cambridge area

B20658		Carter's series)	
CAMSM B20659	Partial rostrum	*P. campylodon* (syntype, Carter's series)	Cambridge area
CAMSM B20671	Rostrum	'*Platypterygius*' sp.	Barrington
CAMSM B75736	Atlas-axis	Ichthyosauria indet.	Cambridge area
CAMSM B42257	Centrum	Ichthyosauria indet.	Hunstanton
CAMSM unnumbered	Humerus (HM1 morphotype of Fischer et al. [36]	'*Platypterygius*' sp.	Cambridge area
NHMUK 5648	Teeth	'*Platypterygius*' sp.	?
NHMUK 33294 partim	Teeth	'*Platypterygius*' sp.	Isleham, Cambridgeshire
NHMUK 41367	Anterior tip of rostrum	'*Platypterygius*' sp.	?
NHMUK 41895	Anterior tip of rostrum	'*Platypterygius*' sp.	?
NHMUK R13	Teeth	'*Platypterygius*' sp.	?
NHMUK R49	Teeth	'*Platypterygius*' sp.	Lyden Spout, Folkestone
NHMUK R2335	Rostrum	'*Platypterygius*' sp.	?
NHMUK R2385	Fragmentary rostrum	'*Platypterygius*' sp.	?

We surveyed the entire Cenomanian collections of both the CAMSM and the NHMUK, but only listed important specimens; unlisted remains include centra, undeterminable skeletal fragments and isolated teeth. The specimens studied here belong to the 'Lower Chalk', which corresponds to the Grey Chalk Subgroup (Chalk Group), above the Cambridge Greensand Member. We found no compelling evidence for the presence of radically distinct species in this deposit, notably in terms of tooth shape and inferred ecological niche.

Supplementary Table 15 | Sampling metrics used in this paper.

	meta.Coll	meta.Occ	meta.Fm	vert.Coll	vert.Occ	vert.Fm	aqua.Coll	aqua.Occ	aqua.Fm
Tur	296	1283	56	20	24	10	8	22	5
Cen	1366	7294	175	140	471	58	129	406	51
U_Alb	616.8	3201.2	82	45.6	167.6	23.6	26.4	106.8	12.4
M_Alb	283.728	1472.552	37.72	20.976	77.096	10.856	12.144	49.128	5.704
L_Alb	641.472	3329.248	85.28	47.424	174.304	24.544	27.456	111.072	12.896
U_Apt	626	2878	93.33333333	22.66666667	45.33333333	11.33333333	25.33333333	66	19.33333333
L_Apt	313	1439	46.66666667	11.33333333	22.66666667	5.666666667	12.66666667	33	9.666666667
Bar	508	2155	63	10	36	5	23	60	19
Hau	651	2607	57	24	38	15	20	41	14
Val	736	2859	73	10	33	4	34	50	20
Ber	441	1906	60	67	241	31	52	150	29

Number of collections, number of occurrences and number of formations for (i) all metazoans in marine setting, (ii) all vertebrates in marine settings, (iii) main aquatic vertebrates (Ichthyosauria, Plesiosauria, Actinopterygii, Actinistia, Dipnoi, Chondrichthyes, Chelonioidea, Mosasauroidea, Dolichosauridae, Pholidosauridae, Hesperornithes) in all settings. These were downloaded from the Paleobiology Database on the 24-25/03/15.

Supplementary Table 16 | Environmental metrics used in this paper.

	Mean_long	Var_long	Mean_short	Var_short	Prok_d180	Prok_d180_var	Mart_SST	Mart_SST_var	Prok_d13C	Prok_d13C_var
Tur	245.54565	88.61105577	201.9056387	478.9346841	-3.210457516	2.170616749	32.2375	19.895625	3.308767123	1.53856455
Cen	237.4940901	54.00933937	192.4588067	1205.833766	-2.474067797	4.382089394	26.95	25.272	3.273559322	0.957522944
U_Alb	212.2396171	335.3116353	172.9053489	1014.133463	-0.910961538	0.092110263	24.041875	11.00381384	1.714038462	0.515110526
M_Alb	170.8115132	29.92690102	136.5678126	233.0992689	-0.165625	0.571160606	24.041875	11.00381384	1.371875	0.657754545
L_Alb	148.6047923	29.12972103	105.5608938	927.7505508	-0.442666667	0.556706667	24.041875	11.00381384	2.352222222	0.714669524
U_Apt	140.59348	4.082983664	104.0955343	645.6224591	-2	0.149	20.75227273	7.081056818	4.958333333	0.242416667
L_Apt	151.1276091	9.931280133	112.502712	362.2929813	-2.325	0.66125	20.75227273	7.081056818	3.35	2.645
Bar	162.2974658	12.91240972	112.1584434	495.9591162	-0.621569231	0.370178313	21.55	0.81	1.010169231	0.651317843
Hau	152.6605727	200.3668724	119.5014559	615.2607866	0.358373494	0.174030345	19.6375	8.285625	1.093803681	0.380823399
Val	92.95908968	207.5907201	68.73486229	394.2535596	-0.042735849	0.446400112	20.8975	9.350125962	0.50402965	0.750753171
Ber	121.0413793	27.03901155	91.95169655	603.6226294	-1.224032258	1.026605057	21.4825	31.7206125	0.450403226	1.281491954

From left to right: (i) mean value of the long term sea level curve (all sea level data from a digitized version of Haq[37]); (ii) variance of the long term sea level curve; (iv) mean value of the short term sea level curve; (ii) variance of the short term sea level curve; (v) weighted mean $d^{18}O$ value (all isotopic values from Prokoph et al.[38]), (vi) variance of $d^{18}O$ values; (vii) mean sea surface temperatures from Martin et al.[39]; (viii) variance of the sea surface temperatures from Martin et al. [39]; (ix) weighted mean d13C value; (x) variance of the d13C value.

Supplementary Table 17 | Results of pairwise correlations tests with a ≥0.05 p value.

Full dataset			Early Cretaceous dataset		
**Correlation**	**Pearson coefficient**	**p value**	**Correlation**	**Pearson coefficient**	**p value**
Sum of Variances (equal) ~ Long term eustatic variance	0.634	0.036	Observed diversity ~ Mean long-term eustasy	0.743	0.022
Sum of Variances (equal) ~ Prokoph d13C	-0.622	0.041	Observed diversity ~ Mean short-term eustasy	0.698	0.037
Evolutionary rate (constrained) ~ Martin Sea surface temperature	-0.739	0.009	Sum of Variances (equal)~ Long term eustatic variance	0.679	0.044
Evolutionary rate (unconstrained) ~ Martin Sea surface temperature	-0.831	0.002	Sum of Variances (equal)~ Prokoph d13C	-0.685	0.042
Extinction rate ~ Short term eustatic variance	0.612	0.045			
Per capita extinction rate ~ Short term eustatic variance	0.742	0.014			
Per capita extinction rate ~ Prokoph d180 variance	0.815	0.004			
Per capita extinction rate	0.644	0.045			

~ Metazoan Collections					
Per capita extinction rate ~ Metazoan Occurrences	0.706	0.022			
Per capita extinction rate ~ Metazoan Formations	0.652	0.041			
Per capita extinction rate ~ Vertebrate Collections	0.796	0.006			
Per capita extinction rate ~ Vertebrate Occurrences	0.821	0.004			
Per capita extinction rate ~ Vertebrate Formations	0.787	0.007			
Per capita extinction rate ~ Aquatic vertebrate Collections	0.755	0.012			
Per capita extinction rate ~ Aquatic vertebrate Occurrences	0.805	0.005			
Per capita extinction rate ~ Aquatic vertebrate Formations	0.739	0.015			
Origination rate ~ Martin Sea surface	-0.604	0.049			

temperature					

Supplementary Table 18. Best models (AICc weight > 0.1 * weight of the best model).

Model	AICc weight	AICc score	R^2	Phi	Slope	Slope p value	Intercept
Observed diversity ~ 1	0.323	49.5707	0	0.358	NA	NA	3.027
Observed diversity ~ Prokoph d13C variance	0.159	50.9884	0.138	0.269	-0.4	0.647	3.464
Observed diversity ~ Prokoph d180	0.144	51.1855	0.123	0.43	0.483	0.452	3.612
Observed diversity ~ Prokoph d13C	0.127	51.4393	0.102	0.534	-0.483	0.361	3.897
Observed diversity ~ Prokoph d180 variance	0.087	52.1855	0.039	0.355	0.048	0.926	2.979
Observed diversity ~ Martin Sea surface temperatures	0.069	52.66	-0.003	0.654	-0.296	0.248	9.911
Phylogenetically adjusted diversity ~ Martin Sea surface temperatures	0.261	53.3441	0.293	-0.048	-0.84	0	27.529
Phylogenetically adjusted diversity ~ Prokoph d13C	0.203	53.8523	0.26	1	-0.93	0.13	7.498
Phylogenetically adjusted diversity ~ 1	0.179	54.1021	0	1	NA	NA	5.75
Phylogenetically adjusted diversity ~ Prokoph d180	0.156	54.3698	0.224	1	0.931	0.247	7.814
Phylogenetically adjusted diversity ~ Prokoph d13C variance	0.106	55.1395	0.168	1	-0.634	0.451	6.645
Phylogenetically adjusted diversity ~ Prokoph d180 variance	0.053	56.5222	0.056	1	-0.071	0.902	5.863
Sum of variances (basic) ~ Prokoph d13C	0.209	51.4935	0.271	0.655	-0.995	0.176	8.408
Sum of variances (basic) ~ 1	0.198	51.6007	0	0.624	NA	NA	6.494
Sum of variances (basic) ~ Prokoph d180	0.175	51.8515	0.245	0.577	0.991	0.329	7.711
Sum of variances (basic) ~ Prokoph d13C variance	0.155	52.0917	0.226	0.648	-0.843	0.444	7.255
Sum of variances (basic) ~ Prokoph d180 variance	0.122	52.5758	0.188	0.504	-0.723	0.319	7.465
Sum of variances (basic) ~	0.081	53.3802	0.12	0.236	-	0.159	22.274

Martin Sea surface temperatures					0.687		
Sum of variances (equal) ~ Prokoph d13C	0.265	44.9348	0.285	1	-0.8	0.097	9.678
Sum of variances (equal) ~ 1	0.228	45.2343	0	1	NA	NA	8.188
Sum of variances (equal) ~ Prokoph d180	0.177	45.7424	0.225	1	0.799	0.223	9.664
Sum of variances (equal) ~ Prokoph d180 variance	0.091	47.0624	0.116	1	-0.445	0.376	9.392
Sum of variances (equal) ~ Prokoph d13C variance	0.081	47.2906	0.095	1	0.001	0.999	8.187
Sum of variances (equal) ~ Martin Sea surface temperatures	0.054	48.1253	0.016	1	-0.083	0.855	10.191
Sum of variances (equal) ~ Martin Sea surface temperatures variance	0.028	49.4614	-0.124	1	-0.107	0.154	11.23
Cladogenesis rate (Max Parsim) ~ 1	0.55	31.1244	0	0.583	NA	NA	0.524
Cladogenesis rate (Max Parsim) ~ Prokoph d180	0.145	33.7918	0.035	0.479	0.297	0.243	0.922
Cladogenesis rate (Max Parsim) ~ Prokoph d13C variance	0.09	34.746	-0.053	0.554	0.044	0.889	0.479
Cladogenesis rate (Max Parsim) ~ Prokoph d13C	0.065	35.4009	-0.117	0.474	-0.109	0.605	0.754
Cladogenesis rate (Max Parsim) ~ Prokoph d180 variance	0.061	35.523	-0.13	0.54	-0.07	0.729	0.601
Cladogenesis rate (Max Parsim) ~ Martin Sea surface temperatures	0.057	35.6535	-0.143	0.539	-0.11	0.233	3.157
Cladogenesis rate (Bayesian, constrained) ~ 1	0.758	8.7123	0	1	NA	NA	0.438
Cladogenesis rate (Bayesian, unconstrained) ~ 1	0.761	8.281	0	1	NA	NA	0.443
Evolutionary rate (constrained) ~ 1	0.741	-19.9549	0	0.971	NA	NA	0.764
Evolutionary rate (constrained) ~ Prokoph d180	0.195	-17.2796	0.037	0.984	0.038	0.012	0.831
Evolutionary rate (unconstrained) ~ 1	0.875	-24.3286	0	1	NA	NA	0.744
Extinction rate~ 1	0.324	48.8026	0	-	NA	NA	2.013

				0.076			
Extinction rate~ Prokoph d13C variance	0.171	50.0817	0.149	-0.106	0.432	0.633	1.615
Extinction rate~ Prokoph d180 variance	0.154	50.2922	0.133	-0.09	0.51	0.256	1.528
Extinction rate~ Prokoph d180	0.093	51.3061	0.049	-0.061	-0.201	0.693	1.774
Extinction rate~ Prokoph d13C	0.072	51.8168	0.004	0.021	-0.117	0.786	2.244
Extinction rate~ Aquatic vertebrates Formations	0.045	52.7498	-0.084	-0.071	0.079	0.07	0.593
Extinction rate~ Vertebrates Formations	0.03	53.5743	-0.169	0.05	0.062	0.082	0.871
Per capita extinction rate ~ Prokoph d180 variance	0.4	9.8152	0.294	-0.291	0.22	0.002	0.098
Per capita extinction rate ~ 1	0.322	10.2471	0	0.928	NA	NA	0.501
Per capita extinction rate ~ Martin Sea surface temperatures	0.107	12.458	0.081	0.54	0.111	0.026	-2.19
Per capita extinction rate ~ Prokoph d180	0.048	14.0426	-0.077	0.551	-0.151	0.132	0.165
Origination rate ~ 1	0.466	36.263	0	0.817	NA	NA	0.953
Origination rate ~ Martin Sea surface temperatures	0.17	38.276	0.09	1	-0.264	0.076	7.08
Origination rate ~ Prokoph d180	0.096	39.4181	-0.009	0.786	0.179	0.606	1.323
Origination rate ~ Prokoph d13C variance	0.09	39.5586	-0.022	0.772	-0.007	0.986	1.077
Origination rate ~ Prokoph d13C	0.069	40.0822	-0.072	0.828	-0.078	0.775	1.077
Origination rate ~ Prokoph d180 variance	0.06	40.3645	-0.1	0.775	-0.027	0.914	1.094
Turnover rate ~ 1	0.326	48.884	0	0.004	NA	NA	2.53
Turnover rate ~ Prokoph d13C variance	0.16	50.3099	0.138	-0.006	0.279	0.757	2.269
Turnover rate ~ Prokoph d13C	0.126	50.786	0.1	0.006	-0.427	0.302	3.438
Turnover rate ~ Prokoph d180 variance	0.107	51.1149	0.072	0.09	0.355	0.466	2.166
Turnover rate ~ Prokoph d180	0.1	51.2441	0.061	0.078	0.237	0.667	2.799

Turnover rate ~ Aquatic vertebrates Formations	0.046	52.7789	-0.079	-0.056	0.08	0.067	1.104
Turnover rate ~ Martin Sea surface temperatures	0.044	52.8788	-0.089	0.145	-0.163	0.366	6.307

Results from generalised least squares regressions incorporating a first-order autoregressive model, using the full dataset. Other variables were tested and resulted in models with negligible AICc-weights (see Supplementary Data 9 GLS_results).

Supplementary Table 19. Best models (AICc weight > 0.1 * weight of the best model)

Model	AICc weight	AICc score	R^2	Phi	Slope	Slope p value	Intercept
Observed diversity ~ 1	0.603	42.1942	0	0.839	NA	NA	4.227
Observed diversity ~ Martin Sea surface temperatures	0.121	45.4141	0.357	0.255	0.796	0.029	-14.03
Phylogenetically adjusted diversity ~ 1	0.65	44.7324	0	0.69	NA	NA	9.36
Phylogenetically adjusted diversity ~ Prokoph d180 variance	0.093	48.6306	0.307	0.629	0.657	0.759	9.011
Phylogenetically adjusted diversity ~ Prokoph d13C	0.087	48.7631	0.297	0.609	-0.827	0.119	10.695
Sum of variances (basic) ~ 1	0.631	50.9365	0	0.508	NA	NA	7.15
Sum of variances (basic) ~ Prokoph d180 variance	0.13	54.0948	0.362	0.486	-0.025	0.994	7.176
Sum of variances (equal) ~ 1	0.693	45.3157	0	1	NA	NA	9.213
Sum of variances (equal) ~ Prokoph d180 variance	0.094	49.3111	0.3	1	0.055	0.979	9.182
Cladogenesis rate (Max Parsim) ~ 1	0.802	32.2745	0	0.552	NA	NA	0.616
Cladogenesis rate (Bayesian, constrained) ~ 1	0.931	14.3994	0	1	NA	NA	0.438
Cladogenesis rate (Bayesian, unconstrained) ~ 1	0.934	14.0544	0	1	NA	NA	0.444
Evolutionary rate (constrained) ~ 1	0.96	-12.1583	0	0.893	NA	NA	0.784
Evolutionary rate (unconstrained) ~ 1	0.993	-21.1458	0	0.625	NA	NA	0.801
Extinction rate~ 1	0.747	42.2323	0	-0.234	NA	NA	1.84
Extinction rate~ Prokoph d180 variance	0.096	46.3391	0.291	-0.236	-0.217	0.913	1.936
Per capita extinction rate ~ 1	0.965	6.1419	0	-0.481	NA	NA	0.188
Per capita extinction rate ~ Prokoph d180 variance	0.014	14.6668	-0.159	-0.463	-0.074	0.716	0.221
Origination rate ~ 1	0.826	32.4348	0	1	NA	NA	1.5
Turnover rate ~ 1	0.73	42.7738	0	0.118	NA	NA	2.565

Turnover rate ~ Prokoph d180 variance	0.095	46.8532	0.293	0.102	0	1	2.561

Results from generalised least squares regressions incorporating a first-order autoregressive model, using the Early Cretaceous dataset. Other variables were tested and resulted in models with negligible AICc-weights (see Supplementary Data 9 GLS_results).

SUPPLEMENTARY NOTES

Supplementary note 1. Specimens considered in Figure 4 of the main paper.

(1) incorporates indeterminate ophthalmosaurines from the Late Albian of the Cambridge Greensand Member[36]; (2) incorporates the large Late Albian platypterygiines of the Vocontian Basin (RGHP PR 1), from the Gault and Upper Greensand formations, from the Late Albian to earliest Cenomanian of the Cambridge Greensand Member[36], and from the Late Cenomanian of the Boulonnais[40]. (3) incorporates indeterminate ophthalmosaurines from the Late Albian of Saratov region (SSU 14/37 837/46) and from the Albian–Cenomanian boundary of western Russia (see Supplementary Methods). (4) incorporates large platypterygiines from Stoilensky quarry and the Cenomanian of western Russia (see Supplementary Methods). (5) incorporates Early Cenomanian material from Texas (DMNH 11843[41]). (6) incorporates the Early Cenomanian specimen(s) mentioned by[42,43]. (7) incorporates platypterygiine material from India (see Supplementary Methods below).

SUPPLEMENTARY METHODS

INSTITUTIONAL ABBREVIATIONS

The following institutional abbreviations are used: BRSMG, City of Bristol Museum and Art Gallery, Bristol, UK; CAMSM, Sedgwick Museum of Earth Sciences, Cambridge University, Cambridge, CM, Carnegie Museum of Natural History, Pittsburgh, PA, USA; UK; DON, Museo Geológico José Royo y Gómez del Instituto de Investigaciones en Geociencias, Minería y Química, Ingeominas, Colombia; GLAHM, The Hunterian Museum, University of Glasgow, Glasgow, UK; IRSNB, Royal Belgian Institute of Natural Sciences, Brussels, Belgium; LMR, Lyme Regis Museum, Lyme Regis, Dorset, UK; MGRI, Moscow Geological Prospecting Institute, Vernadskii State Geological Museum, Moscow, Russia; MHNH, Muséum d'Histoire Naturelle du Havre, Le Havre, France; MNHN, Muséum national d'Histoire naturelle, Paris, France; MJML, Museum of Jurassic marine life, Ashfield, Kimmeridge, Dorset, UK; MOZ, Museo Professor J. Olsacher, Dirección Provincial de Minería, Zapala, Neuquén, Argentina; NHMUK, Natural History Museum, London, UK; RGHP, Réserve naturelle géologique de Haute-Provence, Digne-les-Bains, France; SMNS , Staatliches Museum für Naturkunde Stuttgart, Stuttgart, Germany; SMSS, Städtisches Museum Schloss Salder, Salzgitter, Germany; SNHM, Staatliches Naturhistorisches Museum, Braunschweig, Germany; SSU, Geological Museum, Saratov State University, Saratov, Russia; U.W., University of Wyoming, Laramie, Wyoming; YKM, Ульяновский областной краеведческий музей им И.А. Гончарова [Ulyanovsk Regional Museum of Local Lore named after I.A. Goncharov], Ulyanovsk, Ulyanovsk Region, Russian Federation.

REVISED TAXONOMY OF CRETACEOUS ICHTHYOSAURS FROM EURASIA

Species taxonomically revaluated here are marked with asterisks and taxa incorporated in our phylogenetic analysis are written in bold.

Valid taxa

Ichthyosauria Blainville, 1835[44]

Thunnosauria Motani, 1999[45]

Malawania anachronus Fischer et al., 2013[4]

Baracromia Fischer et al., 2013[4]

Ophthalmosauridae Baur, 1887[46]

Ophthalmosaurinae Baur, 1887[46] sensu Fischer et al.[3]

Acamptonectes densus Fischer et al., 2012[3]

Leninia stellans Fischer et al., 2014[47]

Platypterygiinae Arkhangelsky, 2001[48] sensu Fischer et al. [3]

Caypullisaurus bonapartei Fernández, 1997[49]

Simbirskiasaurus birjukovi Ochev & Efimov, 1985[50]

Sveltonectes insolitus Fischer et al., 2011[51]

'*Platypterygius*' *hauthali* Huene 1927[52]

Platypterygius platydactylus (Broili 1907)[53]

'*Platypterygius*' *sachicarum* Páramo, 1997[35]

'*Platypterygius*' *hercynicus* Kuhn, 1946[33]

Athabascasaurus bitumineus Druckenmiller & Maxwell, 2010[54]

Maiaspondylus lindoei Maxwell & Caldwell, 2006[55]

'*Platypterygius*' *australis* (M'Coy, 1867)[56]

'***Platypterygius*' americanus** (Nace, 1939)[57]

Sisteronia seeleyi Fischer et al., 2014[36]

Cetarthrosaurus walkeri (Seeley, 1869)[58]

'*Platypterygius*' *campylodon* (Carter, 1846)[59]

Pervushovisaurus bannovkensis Arkhangelsky, 1998[60]

Invalid taxa

Cf. *Acamptonectes*: *Ichthyosaurus brunsvicensis* Broili, 1908[61]. See treatment in [3].

Ophthalmosauridae indet.: *Ichthyosaurus doughtyi* Seeley, 1869[58]. See treatment in [36].

Ophthalmosauridae indet.: *Delphinosaurus kiprijanoffi/kiprianoffii* Eichwald, 1853. Eichwald[62] erected *Delphinosaurus kiprijanoffii* on remains (eight mandible fragments, twelve teeth, one rib, two centra, one humerus and one ulna) from the iron-rich sands of the Kursk area (Albian–Cenomanian boundary). He interpreted these remains as those of amphibians, because of the presence of dolphin and reptile features, suggesting an intermediate form in between these groups, hence the name. Nevertheless, he already recognized close affinities with "*Ichthyosaurus*" (see Eichwald, 1853) and he clearly listed *Delphinosaurus* as belonging to the "Ichthyosaures" family in his monograph (Eichwald, 1865).

There are numerous issues with the name *Delphinosaurus kiprijanoffii*. In Eichwald[62], the specific name is written "*kiprijanoffii*", whereas it is written "*kiprianoffii*" in the 1865 monograph. This taxon became rapidly forgotten and later authors erected similar generic and specific names, sometimes on totally different material: Merriam [64] erected *Delphinosaurus* as a new generic name for reception of the Carnian (Late Triassic) species *Ichthyosaurus perrini*[65]. Kuhn[66] noted this generic name was preoccupied and proposed a new replacement name, *Californosaurus*, for the species *I. perrini*. The same year, Kuhn[67](p116) listed *Delphinosaurus kiprijanoffi* (with a single "i" at the end) as problematic taxon included within polycotylid plesiosaurs.

In parallel, Kiprijanoff described numerous remains of ichthyosaurs ("*Ichthyosaurus campylodon*") and plesiosaurs from the Lower Cenomanian phosphorite horizon[68–71] (incorrectly considered as "Neocomian" in the literature[72]). However, the horizon containing these specimens is a bone-bed similar and contemporaneous to the 'greensands' of western Europe; any supposedly articulated remains should therefore be considered with extreme caution. Romer[34] considered a skull reconstruction of *Ichthyosaurus campylodon* figured by Kiprijanoff[68] to be distinct from the British remains and erected the specific name "*kiprijanoffi*" (with a single "i" at the end) , without first-hand examination of the material.

Both specific names *kiprijanoffi* and *kiprianoffii* have the same origin and etymology: they honour the Colonel W. Kiprijanoff for his research on the marine reptiles from the Albian–Cenomanian boundary phosphatic sand of the Kursk region, which started much before his 1880's publications. However, both these species have been erected independently and on different 'specimens' of Kiprijanoff's collection: isolated rostral fragments and postcranial skeleton for *Delphinosaurus kiprijanoffii* Eichwald, 1853 and a supposedly articulated skull for *Myopterygius kiprijanoffi* Romer, 1968.

The remains from Kursk Albian–Cenomanian sand are isolated in a bonebed-like deposit. There is a strong possibility that the remains of *D. kiprijanoffii* figured by Eichwald[63] (in Pl XXXVIII; XL) are actually a composite of the several taxa found in this deposit: some teeth are referable to cf. *Sisteronia*, because of their markedly rectangular cross-section of the root[36]. The partial humerus shows the large trochanters unlike in *Sisteronia*[36] (and V.F. pers. obs. on new material from France) and the large radial and ulnar facets parallel to the sagittal plane. The "ulna" is an ophthalmosaurid epipodial element. Similarly, the articulated skull in Kiprijanoff[68] is most probably a composite, given the nature of their hosting sediments. Whereas the upper part of the Cambridge Greensand Member contains non-reworked early Cenomanian fossils[36], this has never been proved yet for the Kursk bone-bed. Accordingly,

Delphinosaurus kiprijanoffi and *Platypterygius kiprijanoffi* are considered here as a nomina dubia. The specimen referred to as *Platypterygius* cf. *kiprijanoffi* by Bardet[40] possesses large teeth whose roots have a squared cross-section. This material thus differs from the material figured by Eichwald[62] and should not be assigned to *D./P. kiprijanoffii*.

Ichthyosauria indet.: *Ichthyosaurus hildesiensis* Koken, 1883. *Ichthyosaurus hildesiensis* is based on three isolated centra from the "Neocom" of two different localities (Hildesheim and Braunschweig), and a fragmentary snout with a few teeth from Braunschweig[73]. The material is indeterminate, and considered here as Ichthyosauria indet.

Ichthyosauria indet.: *Ichthyosaurus kurskensis* Gutzeit, ? Both Eichwald[62] and Meyer[74] cited "H. Gutzeit" as the authority for the name *I. kurskensis*, but were unable to provide a reference of a paper by Gutzeit to support this claim. Accordingly, the first mention of that name is found in Eichwald[62] and Storrs et al.[75] cited indeed Eichwald, 1853 as the authority of this species. The species is established on large teeth and a large centrum, apparently found together in the "Iron sand" from the Kursk area (western Russia). As will be discussed above, this deposit is reworked; the claim of articulated element is thus doubtful. Moreover, the elements described by Eichwald 1853 lack distinctive features and are to be considered as a nomen dubium and the material transferred to as Ichthyosauria indet.

Ichthyosauria indet.: *Ichthyosaurus polyptychodon* Koken, 1883. This taxon is based on a single partial skull and a few centra from the 'Speeton Clays' of the Hannover area (Germany), so it is likely to come from the same beds as one of the paratypes of *Acamptonectes densus*, SNHM 1284-R[3]. The external exposure of the maxilla is low and appears separated from the margin of the naris by the lacrimal and the premaxilla, unlike in the platypterygiine ophthalmosaurids '*Platypterygius*' *australis* and *Athabascasaurus bitumineus*[31,54]. The prefrontal does not contact the margin of the naris either, unlike in *Aegirosaurus* or *Sveltonectes*[29,51]. The naris is incompletely preserved and the shape of its dorsal surface cannot be used from a taxonomic point of view. Only maxillary teeth are preserved. More than 10 maxillary teeth are present. The crown appears relatively small and blunt, which may be due to the slight heterodonty in ophthalmosaurids tooth rows. Koken[73] indicates that the teeth possess a square shaped cross-section, which may suggest platypterygiine affinities, if genuine. In the absence of other evidence, this taxon is considered here as Ichthyosauria indet.

Ichthyosauria indet.: *Ichthyosaurus steleodon* Bogolubow, 1909. The Barremian strata of the Ulyanovsk region had already yielded ichthyosaur remains prior to *Sveltonectes insolitus* and *Simbirskiasaurus birjukovi*: these remains in questions where described by Bogolubow[76] as *Ichthyosaurus steleodon*. The type and only specimen comprises a fragmentary snout with poorly preserved teeth and a few centra. This material lacks diagnostic features but appears to be twice the size as the small platypterygiines *Sveltonectes insolitus* and *Simbirskiasaurus birjukovi*. Nevertheless, this material is considered here as Ichthyosauria indet. According to Storrs et al.[75], the holotype is housed at the Moscow Geological Prospecting Institute (Vernadskii State Geological Museum, Moscow, Russia). Rozhdestvenskiy[11] considers this material as Late Jurassic in age.

Ichthyosauria indet.: *Ichthyosaurus strombecki* Meyer, 1862. *Ichthyosaurus strombecki* is based on an incomplete teeth-bearing rostrum from Lower Cretaceous of the Braunschweig area (Germany, same locality as *Acamptonectes densus*). The specimen lacks diagnostic features, but Meyer[74] describes the teeth as having a rounded to oval cross-section, presumably throughout, suggesting affinities with Ophthalmosaurinae or *Aegirosaurus*[3,19,77]. However, only the cross-section of the root may have a taxonomic value and Meyer does not mention where he observed that rounded cross-section. The specimen otherwise lacks other diagnostic features. Accordingly, it is considered here as Ichthyosauria indet.

Ichthyosauria indet.: *Gavialis vassiacensis* Cornuel, 1851. Cornuel[78] described a fossil from the Hauterivian of Haute-Marne (France) that he identified as a gavial and proposed the name "*vassiacensis*" for this specimen if it turned to be a new species. This specimen is actually a fragmentary ichthyosaur snout and Cornuel then recognized his mistake[79]. The snout is thin and tubular. The rostrum and the mandible are semi-circular in cross-section and the bones are thick. There is no trace of the lateral fossae, but the dental grooves form pseudo-alveoli[78]. The teeth are conical, elongated and appear to be less than 20 mm high. Only the crown is ridged[78]. This material is too scant and lacks diagnostic features to be identify more precisely than Ichthyosauria indet. It is unclear whether this material or some other was used as part of a composite specimen considered as the holotype of the iguanodontid dinosaur *Heterosaurus neocomensis* by Cornuel[80]. Lapparent & Stchepinsky[81] found evidence for remains belonging to plesiosaurs, *Iguanodon*, and ichthyosaur in the holotype series.

Vertebrata Indet.: *Plesiosaurus nordmanni* Eichwald, 1865. This taxon is based on fragmentary propodial from the 'Neocomian' of Crimea, Russia, originally considered as plesiosaurian by Eichwald[63]. However, both Ryabinin (1946 see Storrs et al.[75]) and Storrs et al.[75] regarded it as indeterminate ichthyosaur. The material was figured by Eichwald[63] and cannot be determined more precisely than Vertebrata indet.

Species inquirenda: *Plutoniosaurus bedengensis* Efimov, 1997. Efimov[82] reported a new stenopterygiine ichthyosaur from the *Speetoniceras versicolor* Zone (upper Hauterivian) of the Ulyanovsk area, for which he proposed a new genus and species, *Plutoniosaurus bedengensis*. Maisch & Matzke[83] assigned *Plutoniosaurus bedengensis* to *Platypterygius* on the basis of several shared features, including the high number of digits (including anterior and posterior accessory digits), the tight mosaic formed by the phalanges, the presence of a preaxial accessory epipodial element, the large trochanter dorsalis of the humerus and the rectangular cross-section of the roots. All these features are now known to be widespread in a clade of ophthalmosaurids, Platypterygiinae. Additional features support this assignation, such as the seemingly strongly reduced extracondylar area of the basioccipital, the unnotched coracoids, and the reduced naris[84]. The material seems well preserved, but Efimov[82] only provides 'idealized' and highly simplified drawings of the specimen. These drawings suggest highly unusual features for *P. bedengensis*, including wide frontals with large temporalis process that are excluded from the temporal fenestra; a lacrimal forming the entire margin of the naris, even anteriorly; teeth with extremely reduced and rounded roots but are described by Efimov[82] as having a subrectangular cross-section. Moreover, the description is succinct and emphasizes features common in post-Triassic ichthyosaurs. Accordingly, the features of this taxon are to be taken with caution until a better redescription.

Plutoniosaurus bedengensis lacks trustworthy diagnostic features and is possibly a representative of the platypterygiine ophthalmosaurid *Simbirskiasaurus birjukovi*, from the same area and nearly coeval strata. Efimov[82] indicated the nares of *Plutoniosaurus bedengensis* were different from *Simbirskiasaurus birjukovi*, but the holotype of *Simbirskiasaurus birjukovi* was described by Ochev & Efimov (1985) before preparation of the naris[84]. Accordingly, *Plutoniosaurus bedengensis* is considered here as species inquirenda, and will not be counted as an additional platypterygiine taxon in diversity analyses. Examination of old photographs of the holotype of *Plutoniosaurus bedengensis* (I. Stenshin, pers. com. July 2015) indicates this taxon possesses a large frontal forming the

anteromedial margin of the supratemporal fenestra, as in Platypterygiinae; we found no notable morphological differences with the coeval taxon *Simbirskiasaurus birjukovi*.

Species inquirenda: *Ichthyosaurus ceramensis* Martin, 1888. Martin[85] described a moderately large ichthyosaur rostrum from the purported Cretaceous of the Seram Island (also known as Ceram) near Timor and New Guinea. The age of the Cretaceous shales of this area are said to be coeval with the Upper Greensand Formation of England and Utatúr Group in India in Martin's paper. The morphology of the teeth, however, appear similar to that of *Temnodontosaurus platyodon*, *T. trigonodon* or even large specimens of *Ichthyosaurus communis*[86–88], with the presence of numerous continuous apicobasal ridges extending from the top of the crown to the root and a reduced to absent distinct layer of acellular cementum. These features markedly contrast with known ophthalmosaurids[89,90]. Accordingly, while *I. ceramensis* cannot be considered as a nomen dubium, it is regarded here as a nomen inquirendum, and will not be counted as a valid species in this work, because the morphological and stratigraphic evidence considering this taxon as a distinct Cretaceous species is too scant.

Species inquirenda: *Platypterygius ochevi* Arkhanglesky et al., 2008. Arkhangelsky et al.[91] reported a new species, *Platypterygius ochevi*, from Albian–Cenomanian boundary glauconitic sands of the Voronezh area, in between Saratov and Kursk. This taxon is however based on fragmentary remains from a juvenile individual, as evidenced by the presence of unfinished bone on humerus and quadrate and the small size of the centra: the largest anterior caudal centrum is c. 6cm wide, most centra are between 3 and 4cm wide). Because this taxon exhibits some particular features, such as the architecture of the forefin, we consider this taxon as valid, but did not assess its phylogenetic position until more complete unambiguously adult material is found. Indeed, ophthalmosaurids develop numerous features of their forefin during ontogeny, as evidenced by a foetal specimen of '*Platypterygius*' *australis* specimen possessing a humerus more similar to those of early ophthalmosaurids and ophthalmosaurines than to those of platypterygiines[92].

Species inquirenda: *Platypterygius campylodon* (Carter, 1846). Carter[59] established the name *Ichthyosaurus campylodon* in a conference abstract. His initial description is based on an articulated rostrum with numerous teeth that he described in a paper the same year[93]. In that paper, he figured two teeth and made clear that his collection contained several

specimens, coming from both the Cambridge Greensand Member and the overlying chalk (Grey Chalk Subgroup). Since Carter's publications, nearly every Cretaceous ichthyosaur remain from Eurasia has been referred to *Platypterygius campylodon* by default[68,70,94–97]. Other remains were referred to the species *kiprijanoffi*[34,40], but these were subsequently assigned to as *Platypterygius campylodon* by McGowan & Motani[98]. At the current state of knowledge, '*Platypterygius*' *campylodon* is a vague entity with no clear-cut morphology nor any valid diagnostic feature. A probable type series has been located in the CAMSM while examining ichthyosaurs for the present paper; a re-description of these specimens is currently being undertaken.

ICHTHYOSAURS FROM THE RUSSIAN EARLY-LATE CRETACEOUS BOUNDARY

A diversified assemblage of vertebrates is preserved within this greensand-like bed. The first marine reptile remains from the Kursk region were described by Eichwald[62,63] and Kiprijanoff[68–71]. In recent years, remains of terrestrial biota have been described from the Stoilensky quarry as well[99]. The composition of the fauna may be summarized as containing numerous ichthyosaurs, plesiosaurs (*Polyptychodon interruptus*, Polycotylidae indet., Elasmosauridae indet.) and hadrosaurs.

Description of selected remains. Interclavicles (SSU 14/8 137/176, SSU 14/8 137/177). The interclavicle is markedly T-shaped, although there is a thin bony sheet laterally to the junction of the anterior transverse bar with the posterior median stem, forming a gently concave edge as in *Caypullisaurus*[49], and unlike the abrupt angle seen in *Sveltonectes insolitus*[51]. The posterior median stem is slender and flat: in SSU 14/8 137/176, the dorsal (internal) surface of the median stem is slightly concave, although not as much as in *Sveltonectes insolitus* (V.F., pers. obs. on holotype). This surface is slightly convex on SSU 14/8 137/177.

Humeri (SSU 14/37 837/46; SSU 14/37). The capitulum is missing in both specimens. The humerus is short and no constricted, which may suggest a juvenile condition. Both the ventral and dorsal trochanters are well developed. There are numerous minute foramina on the shaft of specimen SSU 14/37. There are three distal facets, presumably for anterior accessory element, radius and ulna. The semi-oval anterior facet is the smallest, the square radial facet is the largest and the ulnar facet is semicircular. The ulnar facet is markedly deflected posteromedially while the radial facet faces laterally, a feature of *Arthropterygius* and

ophthalmosaurine ophthalmosaurids[3,100]. These humeri correspond to the 'HM4 morphotype' of the English greensands deposits and are referred to as Ophthalmosaurinae indet. by [36].

Femur (SSU 14/44 137/122). The femur has well developed trochanters, a marked triangular cross-section of the capitulum and an elongated shaft. The fibular facet is slightly deflected posteromedially. The tibial facet is the largest and is deflected anteromedially. The facet for the anterior accessory element is small and nearly in the same plane as the tibial facet. This morphology correspond to the 'FM1 morphotype' in the English greensands deposits and is referred to as *Platypterygius* sp. by [36].

Teeth (SSU GPV 2/). Four distinct morphotypes can be recognized in the assemblage. Three correspond to the morphotypes TM1, TM2 and TM3 defined by Fischer et al. [36] the English greensands deposits. TM1 teeth are the largest, have a squared cross-section and lack prominent angles, unlike in *Pervushovisaurus bannovkensis* and '*Platypterygius*' *campylodon*[60,84]; we refer these teeth to '*Platypterygius*' sp. TM2 teeth have a markedly rectangular root, a smooth acellular cementum ring, and well-marked ridges on the enamel, as in *Sisteronia seeleyi*. In the absence of cranial remains, we refer these teeth to cf. *Sisteronia*. Possible small TM3 are also present; these are referred to this morphotype because of the rounded-cross-section of the root and recurved crown. This morphotype was assigned to Ophthalmosaurinae indet. by Fischer et al.[36]. Diagnostic feature can be hardly discernable on smaller teeth (either from juvenile individuals or from the back of the mandible); yet, because other isolated elements referable to Ophthalmosaurinae are present in Stoilensky, we refer these teeth to as cf. Ophthalmosaurinae. A fourth morphotype is abundant (Supplementary Figure 3) and appears distinct from the three others; the crown and acellular cementum ring are elongated, pointed and slightly recurved, the enamel is only weakly ridged, the root is apicobasally shortened with a slightly quadratic cross-section. These features recall leptonectid ichthyosaurs of the Early Jurassic and more generally soft-prey specialised marine reptiles.

THE CENOMANIAN RECORD OF ICHTHYOSAURS

Europe

The most abundant material from the Cenomanian comes from the lower part of the Grey Chalk Subgroup in England, but rarely contains articulated material. Nearly all ichthyosaur specimens from that deposit have been referred to as '*Platypterygius*' *campylodon*, by default[59,72,93,96,101,102]. While the status of this species is unclear and currently under investigation, all the available material is compatible with large macrophageous

platypterygiine ophthalmosaurids and indicate low taxonomic diversity, probably a single species (V.F. & N.B., pers. obs.). On the contrary, the basal part of the Grey Chalk Subgroup, formed by the Cambridge Greensand Member, contains a higher diversity[36]. Numerous remains have been described from the western part of Paris Basin as well. Morière[103] reported a fragmentary skeleton with teeth, rostrum and centra from the chalk of near Villers-sur-Mer. Blain et al.[104] reported two skull roof elements referred to as cf. *Platypterygius* from the lower Cenomanian (*Hypoturrilites carcitanensis* or *Mantelliceras saxbii* Zones) of the Falaises des Vaches Noires locality (Villers-sur-mer, Calvados). Bardet[40] described a fragmentary but associated skull (referred to as '*Platypterygius*' cf. *kiprijanoffi*) from the upper part of the early Cenomanian *Mantelliceras dixoni* Zone of the Petit Blanc-Nez Formation. Cenomanian teeth from Le Havre, possibly belonging to '*Platypterygius*' *campylodon*, are present in the Muséum national d'Histoire naturelle (Paris, France) collections (MNHN 135). Germany yielded a large number of isolated finds, mainly teeth, of ichthyosaurs ranging from the basal to middle Cenomanian[105–108]. Bardet et al[109] reported the youngest ichthyosaur known so far, from the upper Cenomanian of Bavaria. Finally, Bardet[110] regards the specimens of Capellini[111,112] from Emilia, Italia, as being Cenomanian in age.

Russia

The fossil-rich strata of the neighbouring Kursk and Belgorod regions[62,63,68–71] have yielded a diversified assemblage, which can be compared to those of the Cambridge Greensand Member (UK) and the Annopol anticline (Poland)[16,113]. Our reassessment indicates that the Stoilensky assemblage (Appendix) and other late Albian–Cenomanian localities of the Saratov area contain cranial and postcranial remains referable to as '*Platypterygius*' sp., cf. *Sisteronia* and cf. Ophthalmosaurinae. Teeth from the Stoilensky quarry suggest the presence of a fourth, currently indeterminate taxon. This taxon is not counted as an additional valid species in our analyses because of the scarcity of the remains (isolated teeth). Our preliminary assessment indicates that relative abundances greatly vary but ichthyosaurs dominate the ecosystem (Fig. S 5). Of course, the specie abundance signal is biased by three factors: the total number of teeth for each taxon, the shedding frequency, and sedimentological sorting. Thus, additional material is crucial to gain a less biased insight of the top predator assemblages within the Kursk area at the Early–Late Cretaceous transition. As in the upper Gault/Cambridge Greensand Member ecosystem[36], the Stoilensky ichthyosaur assemblage display three distinct tooth morphotypes, suggesting as much feeding guilds colonised by ichthyosaurs. However, the absence of articulated specimen prevents a complete assessment

of the ecology of these taxa. A notable feature of the Stoilensky fauna is the strong presence of polycotylid teeth, likely belonging to a 'pierce' guild, in conjunction with the ubiquitous but rare apex predator *Polyptychodon* (Fig. S 4-5). Few remains are known from the Cenomanian of Russia besides those of the Kursk and Belgorod regions discussed above. Many isolated and undetermined finds are reported in Pervushov et al.[114] (fourteen specimen in total from the Volga region). The best material comes from the Saratov region, with an articulated rostrum of one of the youngest ichthyosaur species known, the middle Cenomanian platypterygiine *Pervushovisaurus bannovkensis*[60,84].

North America

Both Gilmore and Merriam reported the presence of isolated centra from the 'Benton Cretaceous'[115,116]. However, Slaughter & Hoover[117] consider this material as probably Albian in age. Since then, more complete material has been recovered from early–middle Cenomanian deposits of the Western Interior seaway, belonging to '*Platypterygius*' *americanus* and '*Platypterygius*' sp.[41,57,118,119].

Australia and India

The rare Cenomanian remains from Australia and India complete the current picture of distribution of Cenomanian ichthyosaurs. Kear[43] mentions the presence of ichthyosaur remains in the early Cenomanian of Australia; the material is a specimen consisting of a single phalanx and worn centra[42], that we regard as Ichthyosauria indet. Lydekker[120] reported centra from the Utatúr Group of Trichinopolí, India, which he considered as coeval to the Upper Greensand Formation of England. He referred these centra to a new species, although he was not "*absolutely certain of the specific distinctness of the India form*"[120: 28]. Moreover, Lydekker formally erected the species name, *Ichthyosaurus indicus*, nine years later[121]. The description and figuration of these centra indicate that they lack diagnostic features and the material should be regarded as Ichthyosauria indet. Recently, additional material from the early Cenomanian of India has been attributed to this taxon (although under a novel combination, *Platypterygius indicus*) by [122], uniquely on the basis of biogeography. Part of this material (DUGF/41) is referable to as Platypterygiinae indet. because of the squared cross-section of the root. Tooth size and shape appear variable, but all other teeth should be referred to as Ichthyosauria indet. Verma[123] indicates the presence of Cenomanian to early Turonian ichthyosaurs in the Cauvery Basin, southeast India. However, the material supporting this claim appears to be that of [122], which is restricted to the early Cenomanian.

PHYLOGENETIC DATASET AND METHODS

Review of recent phylogenetic data on ophthalmosaurids

The dataset of [3] has been used in many analyses and has undergone a number of modifications since its publication. A wealth of taxa, characters and character states have been added or modified, but some characters and characters states have been misinterpreted or miscoded. Here, we review the two most important recent modifications.

Roberts et al. 2014 dataset:

- New character: Anterior margin of the jugal: terminates prior to anterior end of lacrimal (0), reaches or surpasses anterior end of lacrimal (1). *Incorporated*. Derived state in *Stenopterygius* is erroneous[124].
- New character: Posterior margin of the jugal: articulates with the postorbital and quadratojugal (0), excluded from the quadratojugal by the postorbital (1). *Not incorporated*. Needs to be redefined as *Macgowania* and many other Triassic ichthyosaur exhibit both states 0 and 1[125]. Coding for *Sveltonectes* should be “?” [51] and coding in *Athabascasaurus* should be “0” or more conservatively “?”[54]. As a result, on *Janusaurus* unambiguously possesses the derived state and the character is therefore not informative.
- New character: Broad postfrontal-postorbital contact: absent (0), present (1). *Incorporated*. Coding for *Ichthyosaurus* should be “0”[83].
- New character: Stapedial shaft in adults: thick (0), slender and gracile (1). *Incorporated with modification*. We added “in posterior view” to the character definition as the derived state is not visible in dorsal or ventral view.
- New character: Ventral process on femur: smaller than dorsal process (0), more prominent (1). *Not incorporated*. We feel the states for this character are ambiguously defined and, as a result, we were unable to code it for many ophthalmosaurids.
- Character state modification of *Stenopterygius quadriscissus*: we feel the state 0&1 better captures the evidence here.
- Character state modifications for *Arthropterygius*. Character 24 stapes head size: facets on the basioccipital indicates state 1[100]. We also found the coding for characters 36, 44, and 51[5] to be erroneous.
- Character state modification of the pelvic girdle of *Caypullisaurus bonapartei*. We do not agree with this interpretation, following [6].

- Character state modification of the quadratojugal and squamosal of *Platypterygius hercynicus*. We consider the extreme depth and changing angle of the facets on the lateral surface of the quadratojugal[126] as strong evidence for inferring the presence of a squamosal.

Arkhangelsky & Zverkov 2014 dataset

- New character: Medial facet for the scapula on coracoid: absent (0), present and well prominent (1). *Not incorporated*. Not parsimony informative in the present dataset (derived state only found in *Stenopterygius aalensis*, and it is 0&1 in *Stenopterygius quadriscissus*). We also found this character to be not independent with the next one.
- New character: Coracoid shape in adults: rounded (length to width ratio less than 1.3 and often close to 1) (0), elongated (length to width ratio greater or equal to 1.5) (1). *Incorporated with modification*. We added « anteroposteriorly » elongated in the character definition to make the difference with the mediolaterally elongated coracoids seen in some shastasaurids for example.
- New character: Intermedium/distal carpal2 contact: absent (0), present (1). *Not incorporated*. We note this character is very likely to vary with ontogeny, so it cannot be unambiguously coded for poorly represented taxa. Moreover, the derived state is directly dependent of the presence or absence of polygonal proximal elements in adults (char74 of the novel dataset) and is also dependent of the forefin architecture (latipinnate/longipinnate, char 71 of the novel dataset).

Additional modifications. In addition of merging recent datasets[3–6,84], we incorporated new morphological data from recent sources[28,127,128] and first hand examination of several OTUs. We also added five Cretaceous taxa (*Platypterygius platydactylus*, ‘*Platypterygius*’ *sachicarum*, ‘*Platypterygius*’ *americanus*, *Sisteronia seeleyi*) and corrected a number of misinterpreted and miscoded character states. We modified five characters and adding seventeen new ones (see below). Furthermore, we think Broili[53] and subsequent authors wrongly oriented the forefin of *Platypterygius platydactylus*. The humerus in his figure 16a in table XIII shows a long, axial trochanter on the left and a fine trailing blade on the right: this strongly suggest it depicts a right humerus in ventral view. Indeed, the humeral trochanter that is axially oriented and closer to the edge is the deltopectoral crest in ophthalmosaurids; additionally, ophthalmosaurid humeri also frequently have a posterior trailing edge, but never

anteriorly[36,129,130]. Thus, the preserved humerus and forefin of *Platypterygius platydactylus* belong to the right side of the animal, not the left one. This substantially alters a number of character states (number of anterior and posterior accessory digits, zeugopodial elements, etc.).

We modified the following characters:

- Character 4 (novel dataset) deep apicobasal grooves on roots, not the very common fine striations. The primitive state is thus restricted to *Macgowania janiceps, Eurhinosaurus longirostris, Suevoleviathan disinteger, Temnodontosaurus* spp. and *Ichthyosaurus communis*.
- Character 9 (novel dataset) anterior process of the maxilla. We feel this character (character 7 in [51]) was hard to code and could result in distinct character states because of slight modifications of the premaxilla-nasal suture. Thus, we redefined this character as follows: external part of the anterior process of the maxilla, in lateral view: extends anteriorly to the anterior border of the naris (including reduced anterior narial opening, if present) (0), don't (1).
- We also split the previously multistate ordered character related to naris shape in two: Character 13: naso-maxillary pillar dividing the naris in two (regardless of the reduction of the anterior portion): absent (0), present (1). Character 14: narialis process of the nasal: absent (0), present (1).
- We split the character related to the anterior part of the coracoid in two (characters 53 and 54 in the novel dataset), because it encompassed two different, independent structures: the shape anteromedial process and the anterior notch.

We added the following characters:

- Character 7: Subnarialis process of the premaxilla: ends anteriorly to posterior end of naris (0), reaches posterior end of naris (1).
- Character 12: Naris size: large, ≥ ½ orbit diameter (0), small, << ½ orbit diameter (1).
- Character 16: lacrimal-prefrontal suture in external view: straight (0), strongly crenulated (1).
- Character 19: External prefrontal–parietal contact: absent (0), present (1). The derived state is a feature unique to *Leptonectes tenuirostris*, *Ichthyosaurus* and *Stenopterygius* [128,131,132].

- Character 21: Anterior part of the postfrontal: simple, unpaired (0), bifurcated in a medial and anterolateral processes (1).
- Character 24: Anterolateral parietal process that connects to parietal: absent (0), present (1).
- Character 26: Supratemporal–stapes contact: absent, the posteroventral process of the supratemporal does not extend up to the shaft of the stapes (0), present (1). The derived state was previously found uniquely in *Ophthalmosaurus* spp. [25,26], but is also found in *Leninia stellans* [47].
- Character 31: Occipital lamella of the quadrate: present, giving the quadrate a U-shape in posterior view (0), reduced, the dorsal part of the quadrate is a simple transversely-compressed lamella (1).
- Character 34: Basioccipital condyle peripheral groove: absent (0), present laterally (1); present laterally and ventrally (2).
- Character 37: Raised opisthotic facet of the basioccipital: absent (0), present (1).
- Character 41: Supraoccipital shape: semioval with reduced ventral notch (0), squared and markedly U-shaped with a deep ventral notch (1).
- Character 64: Posterior accessory epipodial element posterior to ulna: absent (0), present (1); present with associated facet on humerus (2). We interpret the condition in *Caypullisaurus bonapartei* as derived (state 1), possessing a posterior accessory epipodial element and a pisiform, rather than a pisiform and a neomorph [133].
- Character 73: Compact and tightly packed epi- and mesopodial rows: absent, elements are loosely connected (0), present (1).
- Character 83: Wide distal femoral blade: present (0), absent, the distal extremity of the femur being smaller than the proximal one in dorsal view (1).

OTU list. *Mikadocephalus gracilirostris*, the best known euichthyosaurian close to Parvipelvia[83], is used as the outgroup for this analysis. Our coding for *Temnodontosaurus spp.* is based on the two best-known species included in that genus: *T. platyodon* (mostly) and *T. trigonodon*. Of the thirty-six OTUs, twenty-two taxa have been personally examined and four additional ones have been examined using high-resolution photographs provided by colleagues.

We did not assess the phylogenetic position of the following Cretaceous taxa, because of the scarcity of their remains: ‘*Platypterygius*’ *hauthali* (partial forefin), *Cetarthrosaurus*

walkeri (two highly peculiar propodials), ‘*Platypterygius*’ ochevi (partial forefin and fragmentary skeleton of a probably juvenile individual), ‘*Platypterygius*’ campylodon (teeth and partial rostrum), *Maiaspondylus lindoei* (diagnostic material is a partial forefin and a partial skeleton from unborn individual, thereby lacking full expression of its characters and carrying a potentially misleading signal[134]). Some Late Jurassic genera were also omitted for the same reasons: *Nannopterygius enthekiodon* (one strongly weathered skeleton and referred isolated fins[25]) and the controversial ophthalmosaurines *Paraophthalmosaurus* (whose distinctness from *Ophthalmosaurus* is still debated[6,83]) and *Undorosaurus* (*U. trautscholdi* is based on an incomplete forelimb and *U. gorodischensis* has been diagnosed and described on doubtful grounds[83]. We direct the reader to Arkhangelsky & Zverkov[6] for an assessment of the phylogenetic relationships of these ophthalmosaurine taxa. The exclusion of these Late Jurassic taxa from our analyses also slightly mitigate the strong impact of laggerstätten in diversity analyses[135,136]. Indeed, with several highly productive formations all over the world[6,25,29,127,137,138], the Tithonian can be considered as a laggerstätte for pelagic marine reptiles, biasing the results towards high diversity and disparity. Also, the exclusion of these Tithonian taxa should not result in significant alteration of the disparity analyses, as both *Paraophthalmosaurus* and *Undorosaurus* have been regarded as junior or senior synonyms of other ophthalmosaurine genera[6,83], suggesting these taxa do not exhibit extreme morphologies that would be ignored by our disparity analyses.

Outgroup

1. *Mikadocephalus gracilirostris* Maisch & Matzke, 1997

Stratigraphic range: Tschermakfjellet Formation, Ladinian; Grenzbitumenzone of the Besano Formation, Anisian–Ladinian boundary, Middle Triassic.

Geographic range: Middelhook, Isfjord, Spitsbergen; Monte San Giorgio, Tessin, Switzerland.

Data sources: [83,139,140].

Specimen personally examined: None.

Terminal taxa

2. *Hudsonelpidia brevirostris* McGowan, 1995

Stratigraphic range: *Epigondolella quadrata* conodont zone of the Pardonet Formation, lower Norian, Upper Triassic.

Geographic range: Williston Lake, British Columbia, Canada.

Data sources: [98,141].

Specimen personally examined: None.

3. *Macgowania janiceps* (McGowan, 1996b)

Stratigraphic range: *Epigondolella multidentata* and *Epigondolella elongata* conodont Zones (≈ *Drepanites rutherfordi* and lower *Mesohimavatiyes columbianus* ammonite Zones of the Pardonnet Formation, middle Norian, Upper Triassic.

Geographic range: Williston Lake, British Columbia, Canada.

Data sources: [83,98,125,142].

Specimen personally examined: None.

4. *Leptonectes tenuirostris* (Conybeare, 1822)

Stratigraphic range: 'Pre-Planorbis' beds, lowermost Hettangian; upper Pliensbachian, Lower Jurassic.

Geographic range: Street, Somerset and Lyme Regis, Dorset, UK; Baden-Württemberg, Germany; Hauenstein area, Switzerland.

Data sources: [83,88,131,143–147].

Specimen personally examined: MNHN AC.9937; NHMUK R498; NHMUK R3612.

5. *Excalibosaurus costini* McGowan, 1986

Stratigraphic range: Bucklandi Zone of an unnamed formation, lower Sinemurian, Lower Jurassic.

Geographic range: Lilstock, Somerset, UK.

Data sources: [148–150].

Specimen personally examined: BRSMG Cc881.

6. *Eurhinosaurus longirostris* von Jäger, 1856

Stratigraphic range: Lower–middle Toarcian.

Geographic range: Banz, Bavaria and numerous localities in Baden-Württemberg, Germany; Whitby, Yorkshire, UK; Dudelange, Luxembourg; Staffelegg (Canton Aargau), Switzerland; Pic-Saint-Loup (Montagne Noire), Noirefontaine (Franche-Comté), and Marcoux (Vocontian Basin), France.

Data sources: [83,98,151–160].

Specimen personally examined: MNHN 1946-20; NHMUK R3938; NHMUK 5465; RGHP MA 1.

7. ***Suevoleviathan disinteger*** Maisch, 1998

Stratigraphic range: Lower Toarcian, Lower Jurassic.

Geographic range: Holzmaden, Baden-Württemberg, Germany; la Robine-sur-Galabre, Vocontian Basin, France.

Data sources: [83,156,161,162].

Specimen personally examined: RGHP PR 1.

8. ***Temnodontosaurus* spp**. Lydekker, 1889

Stratigraphic range: Hettangian; upper Toarcian.

Geographic range: Lyme Regis, Dorset and Whitby, Yorkshire, England; Banz, Bavaria and numerous localities in Baden-Württemberg, Germany; Arlon, Belgium; Yonne, Millau, and Belmont areas, France.

Data sources: [83,86,156,159,163–169]

Specimen personally examined: CAMSM J 46989; IRSNB R 122: IRSNB R 123; LMR material; NHMUK 2003*; NHMUK R1158.

9. ***Hauffiopteryx typicus*** Maisch, 2008

Stratigraphic range: Early Toarcian, Early Jurassic.

Geographic range: Holzmaden, Bande-Württemberg, Germany; Dudelange, Luxemburg; Ilminster, Somerset, UK.

Data sources: [128,152,170,171].

Specimen personally examined: None.

10. ***Malawania anachronus*** Fischer et al., 2013

Stratigraphic range: late Hauterivian–Barremian (range uncertainty of one specimen).

Geographic range: Chia Gara, Kurdistan, Iraq.

Data sources: [4].

Specimen personally examined: NHMUK R6682.

11. ***Ichthyosaurus communis*** de la Bèche & Conybeare, 1821

Stratigraphic range: ‘Pre-Planorbis’ beds, lowermost Hettangian–late Sinemurian, Early Jurassic. Congeneric specimens have been found in the Pliensbachian.

Geographic range: Street, Somerset and Lyme Regis, Dorset, UK; Lorraine, Belgium. Bennett et al. [172] argues that *I. communis* extends up to the Pliensbachian; however, the specimen they described, NHMUK R15907, which V.F. personally examined, differs from other specimens currently referred to as *I. communis* in a number of features of the braincase and hind fin; moreover, their interpretation of numerous bones is incorrect, mixing up scapulae for quadratojugals and clavicles for scapulae. Accordingly we do not consider this specimen as a valid post-Sinemurian occurrence of *I. communis* until more robust arguments are presented.

Data sources: [83,87,143,144,172–177].

Specimen personally examined: GLAHM V1180a; GLAHM V1190; LMR material and private collections in Lyme Regis; MNHN 9862; numerous specimens from NHMUK including NHMUK R1664, NHMUK R5595.

12. *Stenopterygius quadriscissus* (Quenstedt, 1856)

Stratigraphic range: Lower Toarcian; Lower Jurassic.

Geographic range: Holzmaden, Baden-Württemberg; Dobbertin, Germany; Dudelange, Luxembourg.

Data sources: [124,152,170,178–180].

Specimen personally examined: IRSNB 22669; NHMUK R4086.

13. *Stenopterygius/Chacaicosaurus cayi* Fernández, 1994

Stratigraphic range: *Emileia giebeli* Subzone, *E. multiformis* Zone of the Los Molles Formation, lower Bajocian, Middle Jurassic.

Geographic range: Chacaico Sur, Neuquén Basin, Argentina.

Data sources: [181,182].

Specimen personally examined: MOZ 5803.

14. *Stenopterygius aalensis* Maxwell et al., 2012

Stratigraphic range: *Torulosum* Subzone, *opalinum* Zone of the Opalinuston Formation, Lower Aalenian, Middle Jurassic.

Geographic range: Near Zell am Aichelberg, Baden-Württemberg, Germany.

Data sources: [128].

Specimen personally examined: SMNS 90699 (photographs provided by P. Vincent, pers. com. 2012).

15. *Ophthalmosaurus icenicus* Seeley, 1874

Stratigraphic range: Oxford Clay Formation (middle Callovian); Kimmeridge Clay Formation (*cymodoce* to *pectinatus* zones, lower Kimmeridgian–lower Tithonian), Upper Jurassic. Possible congeneric specimens have been reported from the lower Berriasian.

Geographic range: Southeastern England, UK; possibly northern France.

Data sources: [3,25,183–191].

Specimen personally examined: Multiple specimens in CAMSM; GLAHM V1874, GLAHM V1870; MJML material (yet unnumbered); Multiple NHMUK specimens including NHMUK R2133, NHMUK R3702.

16. *Ophthalmosaurus natans* (Marsh, 1878)

Stratigraphic range: "*Sauranodon* beds" = Red Water shale Member, Sundance Formation, upper Callovian–middle Oxfordian, Middle–Upper Jurassic (Massare & Young 2005; Massare et al. 2006; Wahl 2009).

Geographic range: Numerous localities in Wyoming, USA (Massare et al. 2006}).

Data sources: [26,54,187,192–199].

Specimen personally examined: Multiple CM specimens including CM 603.

17. *Mollesaurus perialus* Fernández, 1999

Stratigraphic range: *Emileia giebeli* ammonite Zone of the Los Molles Formation, lower Bajocian, Middle Jurassic.

Geographic range: Chacaico Sur, Neuquén Basin, Argentina.

Data sources: [28,138,181,200].

Specimen personally examined: MOZ 2282 V (photographs provided by and examined with M. Fernández pers. com. September 2014).

18. *Acamptonectes densus* Fischer et al., 2012

Stratigraphic range: D2D horizon of the Speeton Clay Formation, basal Hauterivian; C7F–C7D horizons of the Speeton Clay Formation, lower–middle Hauterivian; *Simbirskites concinnus*/*staffi* Zone, upper Hauterivian, Lower Cretaceous.

Geographic range: Speeton and Filey, North Yorkshire, UK; Cremlingen, Lower Saxony, Germany.
Data sources: [3,36].
Specimen personally examined: GLAHM 132855; NHMUK R11185; SNHM1284-R.

19. *Leninia stellans*
Stratigraphic range: *Deshayesites volgensis* Zone, Lower Aptian, Lower Cretaceous.
Geographic range: Kriushi, Sengiley district, Ulyanovsk Region, Russia.
Data sources: [47].
Specimen personally examined: YKM 65931.

20. *Brachypterygius extremus* (Boulenger, 1904)
Stratigraphic range: *Aulcostephanoides mutabilis* and *Pectinates wheatleyensis* zones of the Kimmeridge Clay Formation, middle Kimmeridgian and lower Tithonian, respectively (McGowan & Motani 2003).
Geographic range: Weymouth, Dorset; Stowbridge, Norfolk, UK.
Data sources: [25,27,98,183,201–205].
Specimen personally examined: BRSMG Cc 16696; CAMSM J68516; NHMUK R3177. V.F. have also examined a cast of the type specimen of *Ichthyosaurus cuvieri* Valenciennes, 1861 (eudoxus Zone Kimmeridgian) [203] held at the MNHN; this taxon is regarded as a possible specimen of *Grendelius* (=*Brachypterygius*) par Bardet et al. [183]. We agree with this assignation, however, only the specimens referable to the species *B. extremus* were used to code this taxon in the dataset.

21. *Arthropterygius chrisorum* (Russell, 1993)
Stratigraphic range: Ringnes Formation, Oxfordian to Kimmeridgian, Upper Jurassic (one specimen). Congeneric specimens have been found in Tithonian strata.
Geographic range: Cape Grassy, Melville Island, Northwest Territories, Canada. Congeneric specimens have been found in Argentina and Russia.
Data sources: [100,206–208] (with a strong focus on the remains referable to *A. chrisorum*).
Specimen personally examined: None.

22. *Caypullisaurus bonapartei* Fernández, 1997

Stratigraphic range: Numerous horizons within the Vaca Muerta Formation, lower Tithonian, Upper Jurassic to lower Berriasian, Lower Cretaceous.
Geographic range: Numerous localities in Neuquén Basin (Neuquén and Mendoza Provinces), Argentina.
Data sources: [49,133,138,209].
Specimen personally examined: MOZ 6139 and photographs of MOZ 6067 provided by M. Fernández (pers. com. September 2014).

23. ***Aegirosaurus leptospondylus*** (Wagner, 1853)
Stratigraphic range: Solnhofen Formation, Malm ζ2b, lowermost Tithonian.
Geographic range: Solnhofen; Eichstätt, Bavaria, Germany. A congeneric specimen has been reported from the Upper Valanginian.
Data sources: [19,29,77,210–213]
Specimen personally examined: NHMUK 42833 and RGHP LA 1; although the coding is primarily based on the specimens referred to *Aegirosaurus leptospondylus*.

24. ***Athabascasaurus bitumineus*** Druckenmiller & Maxwell, 2010
Stratigraphic range: Wabiskaw Member of the Clearwater Formation, lowermost Albian, Lower Cretaceous.
Geographic range: Syncrude Canada Ltd. base mine, near Mildred Lake, Alberta, Canada.
Data sources: [54,199].
Specimen personally examined: None, photographs of holotype (TMP 2000 2901) provided by A. Wolniewicz (pers. com. April 2015).

25. ***Sveltonectes insolitus*** Fischer et al., 2011
Stratigraphic range: Unknown formation, upper Barremian, Lower Cretaceous.
Geographic range: Ulyanovsk area, Ulyanovsk region, Russia.
Data sources: [51].
Specimen personally examined: IRSNB R269.

26. ***Simbirskiasaurus birjukovi*** Ochev & Efimov, 1985
Stratigraphic range: Probably *Praeoxyteuthis pugio* Zone, Lower Barremian, Lower Cretaceous.

Geographic range: Right bank of the Volga River, 25 km above the town of Ulyanovsk, between the Zakhar'yevskoye mine and the Detskiy sanatorium. Russia.
Data sources: [50,84].
Specimen personally examined: YKM 65119.

27. ***Platypterygius australis*** (McCoy, 1867)
Stratigraphic range: Bulldog Shale, Aptian; Wallumbilla Formation, lower Aptian–upper Albian; Darwin Formation, late Aptian–Albian; Allaru Mudstone, middle–upper Albian; Toolebuc Formation, upper Albian; Alinga Formation, upper Albian–Cenomanian; Molecap Greensand, Cenomanian–Turonian, Lower–Upper Cretaceous [214 and references therein]. Kear [43], however, considers *P. australis* to be restricted to the middle–upper Albian.
Geographic range: Numerous localities across Australia, see Kear [43] for a review.
Data sources: [31,32,56,214–216].
Specimen personally examined: NHMUK unnumbered, two juvenile specimens.

28. ***Pervushovisaurus bannovkensis*** Arkhangelsky, 1998
Stratigraphic range: Probably Melovatskaya Formation, Lower–middle Cenomanian, Upper Cretaceous.
Geographic range: Nizhnaya Bannovka, Krasnoarmeisk District, Saratov Region, Russia.
Data sources: [60,84,114].
Specimen personally examined: SSU 104a/24.

29. ***Platypterygius hercynicus*** (Kuhn, 1946)
Stratigraphic range: Neocomer Erzhorizont, upper Aptian; lower *Callihoplites auritus* ammonite Subzone (*Mortoniceras inflatum* ammonite Zone), upper Albian, Lower Cretaceous.
Geographic range: Salzgitter, Lower Saxony, Germany; Saint-Jouin-Bruneval, Seine-Maritime, France.
Data sources: [33,126,217].
Specimen personally examined: Cast of SMSS 'SGS'; MHNH 2010.4.

30. ***Platypterygius americanus*** (Nace, 1939)

Stratigraphic range: Mowry Shale Member of the Graneros Formation, upper Albian; Ashville Formation, Albian-Cenomanian; Belle Fourche Shale; Lower Cenomanian, Lower–Upper Cretaceous.

Geographic range: Crook County, Wyoming; Southern Saskatchewan, Canada.

Data sources: [34,57,118,119].

Specimen personally examined: U.W 2421 (photographs provided by E. Maxwell, pers. com. February 2015).

31. ***Platypterygius platydactylus*** Broili, 1907

Stratigraphic range: *Deshayesites deshayesi* Zone, Lower Aptian, Lower Cretaceous.

Geographic range: Castendamm, near Hannover, Lower Saxony, Germany.

Data sources: [53], reinterpreted here; see above.

Specimen personally examined: None.

32. ***Platypterygius sachicarum*** Páramo, 1997

Stratigraphic range: Arcillolitas Abigarradas Member of the Paja Formation, early Aptian.

Geographic range: Loma Pedro Luis, near Villa de Leiva, Boyacá, Columbia.

Data sources: [21,35,218].

Specimen personally examined: DON-19671 (photographs provided by E. Maxwell, pers. com. February 2015).

33. ***Palvennia hoybergeti*** Druckenmiller et al., 2012

Stratigraphic range: Dorsoplanites ilovaiskyi to Dorsoplanites maximus zones, Slottsmøya Member, Agardhfjellet Formation, Tithonian, Upper Jurassic (one specimen).

Geographic range: Janusfjellet, Spitsbergen, Norway.

Data sources: [5,127].

Specimen personally examined: none.

34. ***Cryopterygius kristiansenae*** Druckenmiller et al., 2012

Stratigraphic range: Dorsoplanites ilovaiskyi to Dorsoplanites maximus zones, Slottsmøya Member, Agardhfjellet Formation, Tithonian, Upper Jurassic (one specimen).

Geographic range: Janusfjellet, Spitsbergen, Norway.

Data sources: [5,127].

Specimen personally examined: none.

35. *Janusaurus lundi* Roberts et al., 2014

Stratigraphic range: Slottsmøya Member, Agardhfjellet Formation, Tithonian, Upper Jurassic.

Geographic range: Janusfjellet, Spitsbergen, Norway.

Data sources: [5].

Specimen personally examined: none.

36. *Sisteronia seeleyi* Fischer et al., 2014

Stratigraphic range: Middle Albian–earliest Cenomanian.

Geographic range: Sisteron and Bevons, Vocontian Basin, France; Cambridgeshire, UK. Possible congeneric specimens are found in Russia (this work).

Data sources: [36].

Specimen personally examined: Several tens of specimens at CAMSM, NHMUK, GLAHM, and RGHP; see Fischer et al. [36] for a complete list.

Character list. Characters are polarized with respect to *Mikadocephalus gracilirostris* as outgroup. As a general rule, we tried to avoid continuous characters, characters clearly related to ecology such as crown shape, or characters based on ratios with ambiguous state boundaries. We illustrate some character states. Characters are polarized with respect to *Mikadocephalus gracilirostris* as outgroup. As a general rule, we tried to avoid continuous characters, characters clearly related to ecology such as crown shape, or characters based on ratios with subjective state boundaries. We illustrate the states of selected characters.

Dentition

1. **Crown striations**: presence of deep axial ridges (0), crown enamel subtly ridged or smooth (1) [54]: character 25.

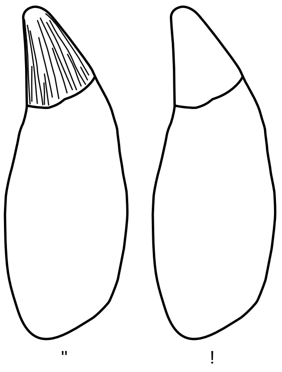

Teeth in lateral view illustrating character 1.

2. **Base of enamel layer on crown**: weakly defined, invisible (0), well defined, precise (1). This appears variable along the rostrum/jaw in *T. platyodon* (IRSNB R 122): the crown enamel is well defined in the anterior-most teeth and then becomes poorly defined in the rest of the jaw. Therefore, only take the teeth from the middle part of the rostrum/jaw. It seems however rather constant for all other ichthyosaurs we have examined. [51]: character 2.

3. **Root cross-section in mid-jaw teeth of adults**: rounded (0), quadrangular (1). [51]: character 3

4. **Deep apicobasal grooves in root**: present (0), absent (1).

Skull

5. **Overbite**: absent or slight (0), clearly present (1) [45]: character 33.

6. **Processus supranarialis of the premaxilla**: present (0), absent (1) [83]: character 10.

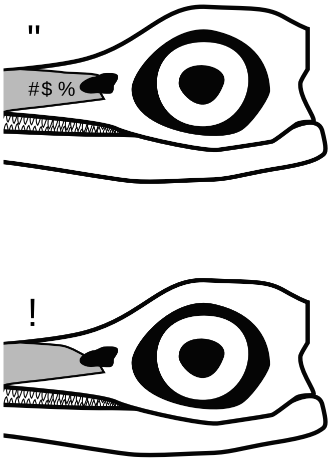

Skull in lateral view illustrating character 6.

7. **Subnarialis process of the premaxilla**: ends anteriorly to posterior end of naris (0), reaches posterior end of naris (1).

8. **Processus postpalatinis of the pterygoid**: absent (0), present (1) [83]: character 38.

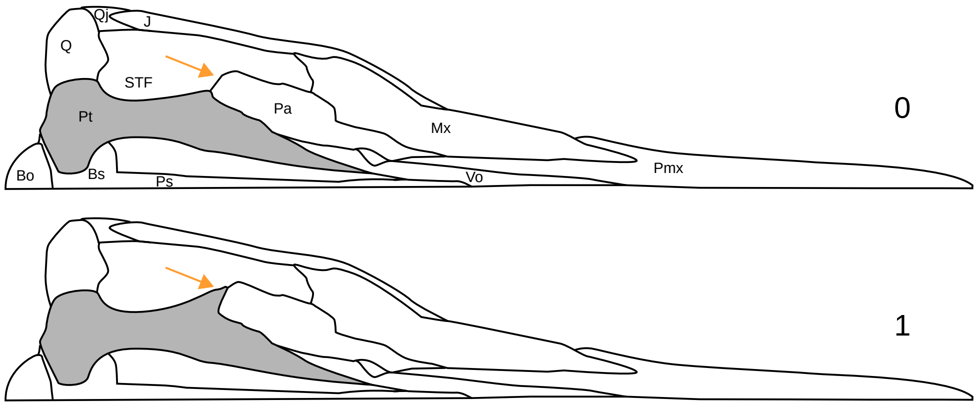

Skull in ventral view illustrating character 8.

9. **External part of the anterior process of the maxilla, in lateral view**: extends anteriorly to the anterior border of the naris (including reduced anterior narial opening, if present) (0), don't (1).[51]:character 7, modified.
10. **External exposure of the maxilla:** large, well visible (0), extremely reduced, nearly absent in external view by processes of the premaxilla and the lacrimal (1).
11. **Processus narialis of the maxilla in external view**: present (0), absent (1). [51]: character 9, modified by[4]

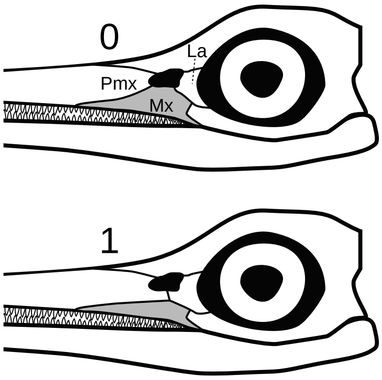

Skull in lateral view illustrating character 11.

12. **Naris size**: large, ≥ ½ orbit diameter (0), small, << ½ orbit diameter (1).
13. **Naso-maxillary pillar dividing the naris in two (regardless of the reduction of the anterior portion)**: absent (0), present (1).
14. **Narialis process of the nasal**: absent (0), present (1).

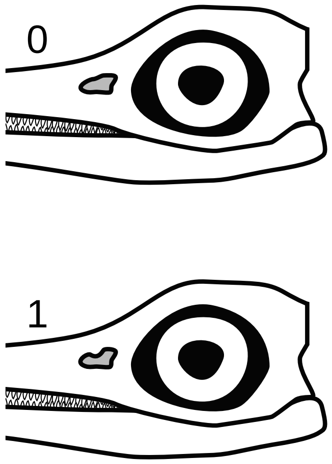

Skull in lateral view illustrating character 14.

15. **Processus narialis of prefrontal:** absent (0), present (1). [51]: character 11.

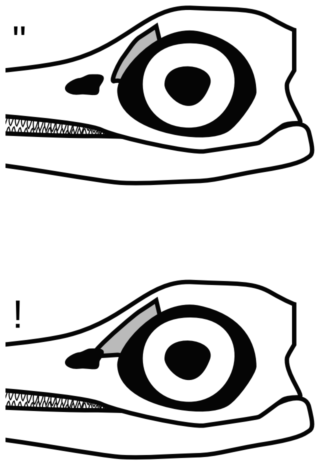

Skull in lateral view illustrating character 15.

16. **Lacrimal-prefrontal suture in external view**: straight (0), strongly crenulated (1).
17. **Anterior margin of the jugal**: tapering, running between lacrimal and maxilla (0), broad and fan-like, covering large area of maxilla ventrolaterally (1) [54]: character 6.

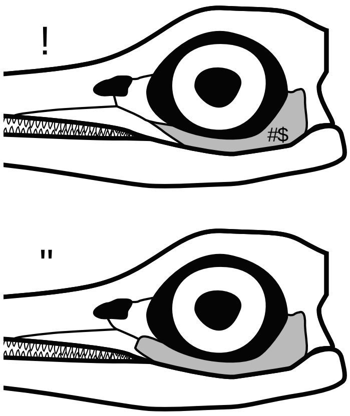

Skull in lateral view illustrating character 17.

18. **Anterior margin of the jugal II**: terminates prior to anterior end of lacrimal (0), reaches or surpasses anterior end of lacrimal (1). [5] : character 11
19. **External prefrontal–parietal contact**: absent (0), present (1).
20. **Processus temporalis of the frontal**: absent (0), present (1). [51]: character 14

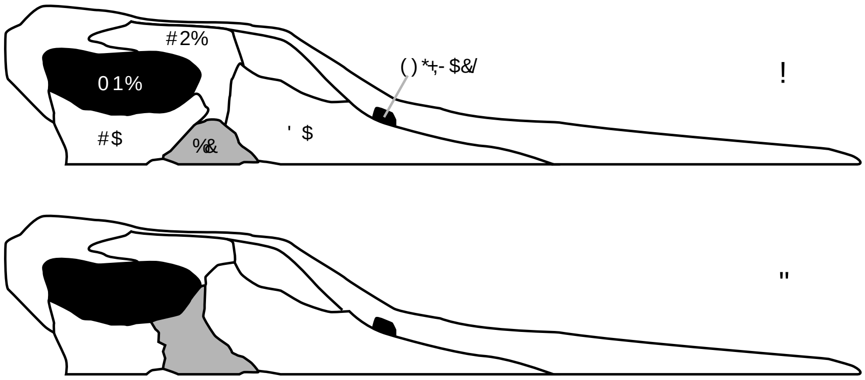

Skull in dorsal view illustrating character 20.

21. **Anterior part of the postfrontal**: simple, unpaired (0), bifurcated in a medial and anterolateral processes (1).
22. **Supratemporal-postorbital contact**: absent (0), present (1) ([219]: character 27, inverted coding).
23. **Broad postfrontal-postorbital contact**: absent (0), present (1). [5]: character 16.
24. **Anterolateral parietal process that connects to parietal**: absent (0), present (1).
25. **Sagittal eminence of the parietal**: present (0), absent (1) ([209]: character 5, inverted coding).
26. **Supratemporal–stapes contact:** absent, the posteroventral process of the supratemporal does not extend up to the shaft of the stapes (0), present (1).

27. **Supratemporal fenestra reduction**: absent, the supratemporal fenestra is large, elongated and its anterior margin is set at the level of the parietal foramen or more anteriorly (0), reduced, the supratemporal fenestra is small, rounded, and its anterior margin is set posterior to the parietal foramen (1). [128]: characters 14 & 15, modified.
28. **Squamosal shape**: square (0), triangular (1), squamosal absent (2). [51]: character 16, inverted coding.
29. **Quadratojugal exposure**: extensive (0), small, largely covered by squamosal and postorbital (1) [83]: character 30, modified.
30. **Lower temporal embayment between jugal and quadratojugal (=jugal–quadratojugal notch or incisura postjugalis)**: present (0), lost (1) [219]: character 25, modified.
31. **Occipital lamella of the quadrate:** present, giving the lateral surface of the quadrate a U-shape in posterior view (0), reduced, the dorsal part of the quadrate is a simple transversely-compressed lamella (1).

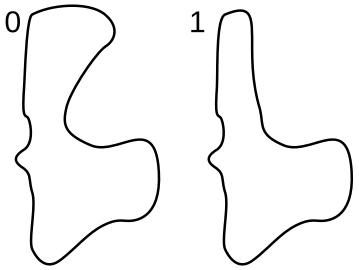

Right quadrate in posterior view illustrating character 31.

32. **Basipterygoid processes**: short, giving basisphenoid a square outline in dorsal view (0), markedly expanded laterally, being wing-like, giving basisphenoid a marked pentagonal shape in dorsal view (1). [51]: character 18.

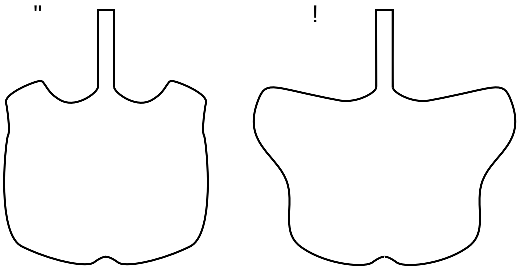

Basisphenoid in ventral view illustrating character 32.

33. **Extracondylar area of basioccipital**: wide (0), reduced but still present ventrally and laterally (1); extremely reduced, being non-existent at least ventrally (2) [209]: character 10, modified.
34. **Basioccipital condyle peripheral groove:** absent (0), present laterally (1); present laterally and ventrally (2).
35. **Basioccipital peg**: present (0), absent (1) [45]: character 29, modified by [51].
36. **Ventral notch in the extracondylar area of the basioccipital:** present (0), absent (1). [3].
37. **Raised opisthotic facet of the basioccipital**: absent (0), present (1).
38. **Shape of the paroccipital process of the opisthotic:** short and robust (0), elongated and slender (1). [3]:character 20.
39. **Stapedial shaft in posterior view in adults**: thick (0), slender and gracile (1). [5], definition modified.
40. **Stapes proximal head**: slender, much smaller than opisthotic proximal head (0), massive, as large or larger than opisthotic (1) [219]: character 34, modified by [51]

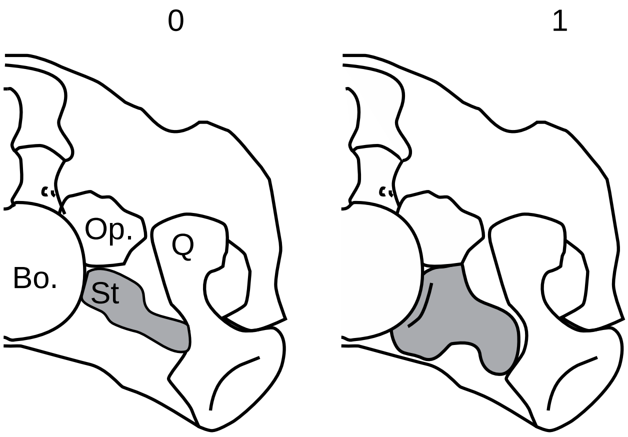

Skull in posterior view illustrating character 40.

41. Supraoccipital shape: semioval with reduced ventral notch (0), squared and markedly U-shaped with a deep ventral notch (1).

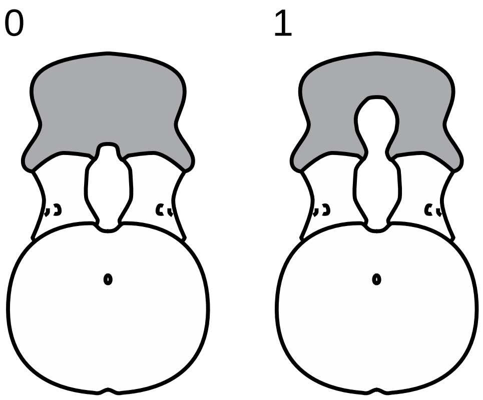

Partial basicranium in posterior view illustrating character 41.

Mandible

42. **Angular lateral exposure**: much smaller than surangular exposure (0), extensive (1) [45]: character 32, inverted coding.

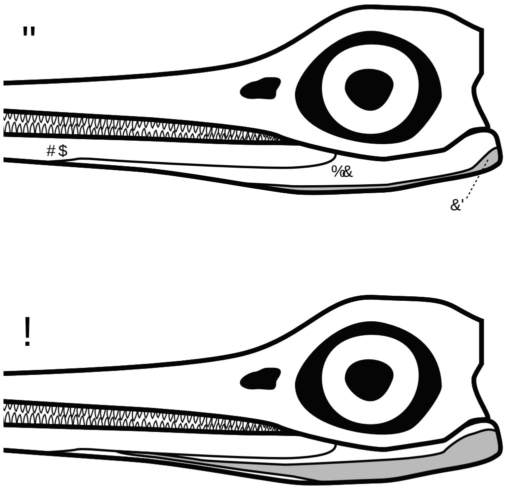

Skull in lateral view illustrating character 42.

Axial skeleton

43. **Posterior dorsal/anterior caudal centra**: 3.5 times or less as high as long (0), four times or more as high as long (1) [100]: character 15, inverted coding.
44. **Tail fin centra**: strongly laterally compressed (0), as wide as high (1) [100]: character 16, inverted coding.
45. **Neural spines of atlas-axis**: completely overlapping, may be fused (0), functionally separate, never fused (1) [54]: character 26.
46. **Chevrons in apical region**: present (0), lost (1) [219]: character 72.

47. **Rib articulation in thoracic region**: predominantly unicapitate (0), exclusively bicapitate (1) [83]: character 53.
48. **Rib cross-section at mid-shaft**: rounded and robust (0), '8'-shaped (1) [219]: character 73, modified.
49. **Ossified haemapophyses**: present (0), absent (1) [83]: character 63.
50. **Tail size**: as long or longer than the rest of the body (0) distinctly shorter (1) [83]: character 65.
51. **Lunate tailfin**: no (0) well-developed (1) [83]: character 66.

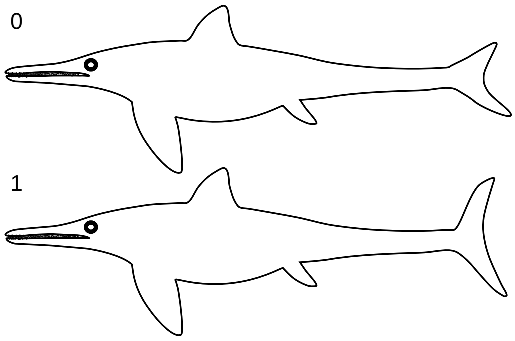

Illustration of character 51.

Scapular girdle and forefin

52. **Coracoid shape in adults**: rounded (length to width ratio less than 1.3 and often close to 1) (0), anteroposteriorly elongated (length to width ratio greater or equal to 1.5) (1). [6]: character 53, definition modified
53. **Anteromedial process of the coracoid**: absent (0), present (1).

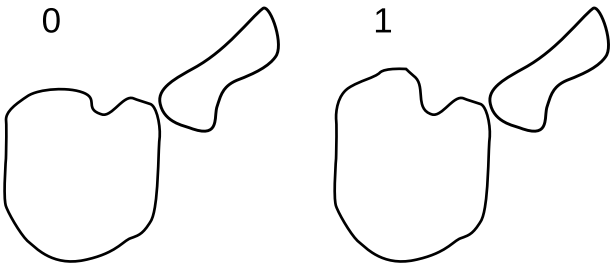

Right coracoid in ventral view illustrating character 53.

54. **Anterior notch of the coracoid**: present (0); absent (1) [51]: character 29, modified.
55. **Glenoid contribution of the scapula**: extensive, being at least as large as the coracoid facet (0), reduced, being markedly smaller than the coracoid facet (1). [3]: character 27.

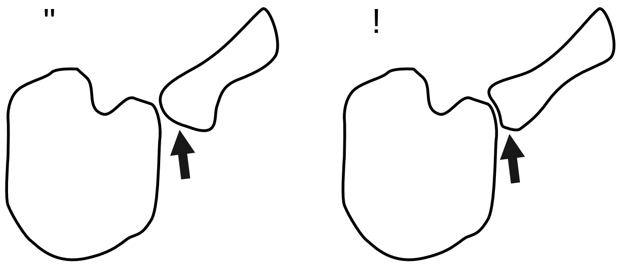

Partial scapular girdle in ventral view illustrating character 54.

56. **Prominent acromion process of scapula:** absent (0), present (1). [51]: character 28.

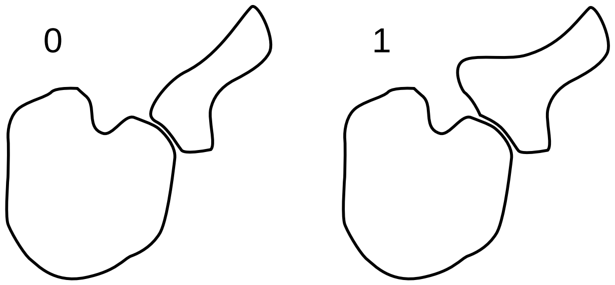

Partial scapular girdle in ventral view illustrating character 55.

57. **Plate-like dorsal ridge on humerus**: absent (0), present (1) [45]: character 56.

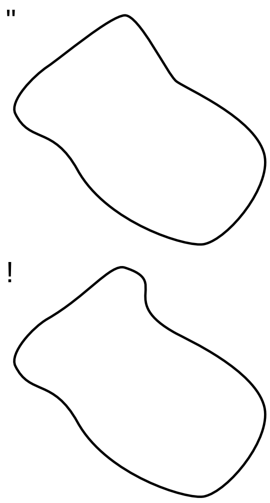

Humerus in proximal view illustrating character 57.

58. **Protruding triangular deltopectoral crest on humerus**: absent (0), present (1); present and very large, matching in height the trochanter dorsalis, and bordered by concave areas (2). [51]: character 31, modified by [3].

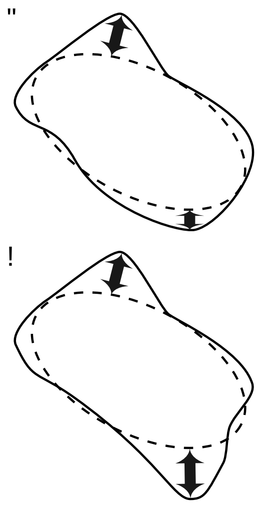

Humerus in proximal view illustrating character 58.

59. **Humerus distal and proximal ends in dorsal view (thus regardless of the size of the dorsal and ventral processes)**: distal end wider than proximal end (0), nearly equal or proximal end slightly wider than distal end (1) [45]: character 55, modified by [51].
60. **Anterior accessory epipodial element anterior to radius**: absent (0), present (1); present with associated facet on humerus (2) [220]: character 10, modified by [51].

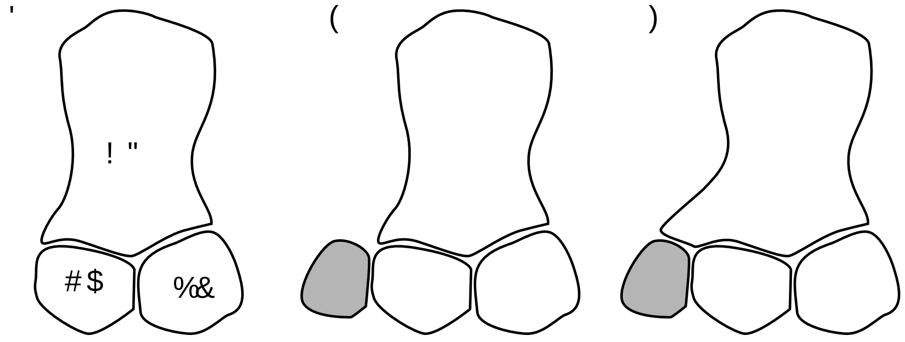

Partial forefin in dorsal view illustrating character 60.

61. **Humerus with posterodistally deflected ulnar facet and distally facing radial facet**: absent (0), present (1). [51]: character 34, modified by [3].

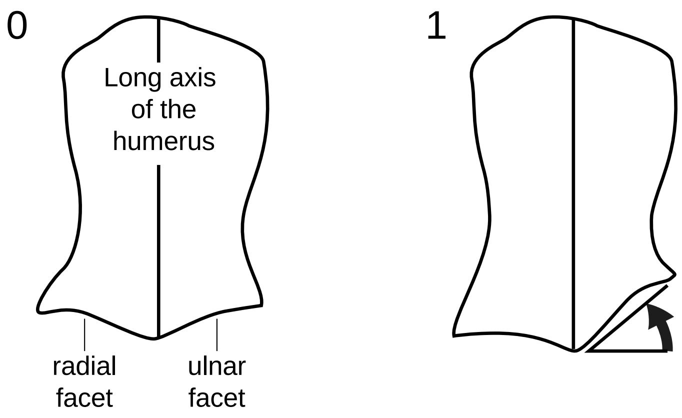

Humerus in dorsal view illustrating character 61.

62. **Humerus/intermedium contact**: absent (0), present (1) [209]: character 15.

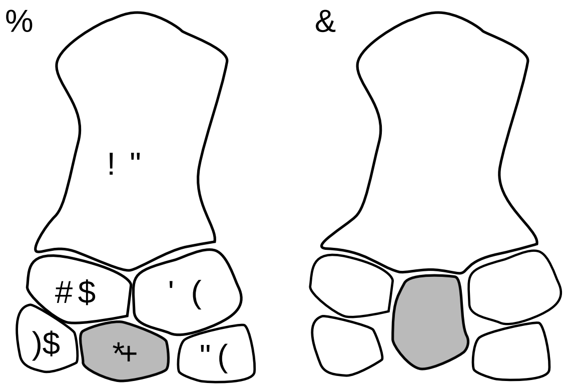

Partial forefin in dorsal view illustrating character 62.

63. **Anterodistal extremity of the humerus**: prominent leading edge tuberosity (0), acute angle (1). [4]: character 44.

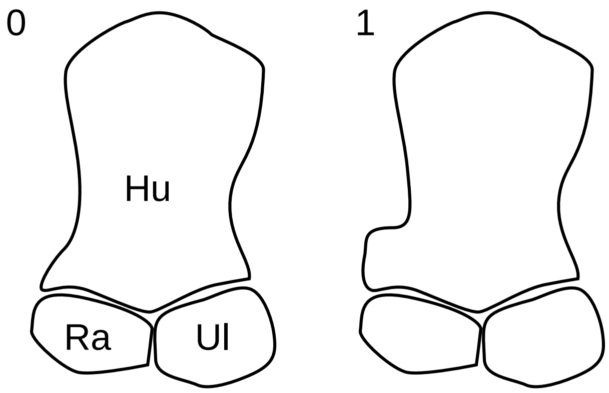

Partial forefin in dorsal view illustrating character 63.

64. **Posterior accessory epipodial element posterior to ulna**: absent (0), present (1); present with associated facet on humerus (2).

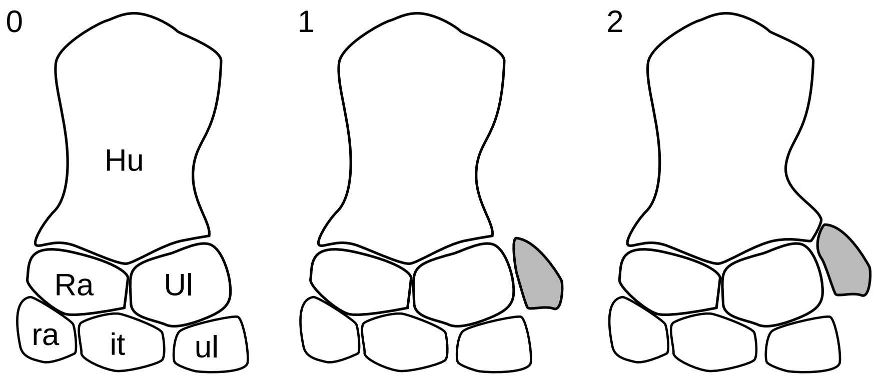

Partial forefin in dorsal view illustrating character 64.

65. **Shape of the posterior surface of the ulna**: rounded or straight and nearly as thick as the rest of the element (0), concave with a thin, blade-like margin (1). [3] :character 36.

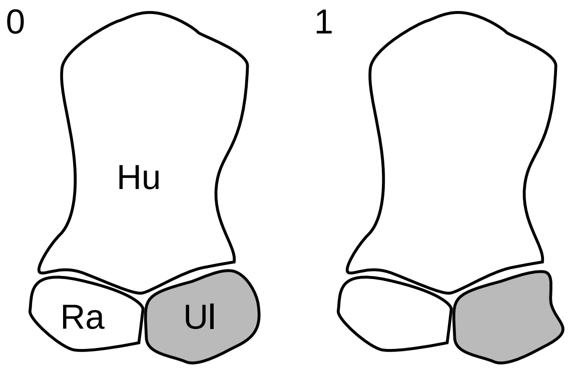

Partial forefin in dorsal view illustrating character 65.

66. **Spatium interosseum between radius and ulna**: present as a space or foramen (0), absent (1) [83]: character 84, modified by [3].
67. **Manual pisiform**: absent (0), present (1) [45]: character 67, inverted coding.
68. **Notching of anterior facet of leading edge elements of forefin in adults**: present (0), absent (1) [45]: characters 59 and 65, modified by [51].
69. **Preaxial accessory digits on forefin**: absent (0), one (1); two or more (2) [83]: character 91, modified.
70. **Posterior enlargement of forefin**: number of postaxial accessory 'complete' digits: none (0), one (1), two or more (2) [83]: character 89, modified by [51].
71. **Longipinnate or latipinnate forefin architecture**: one (0), two (1) digit (s) directly supported by the intermedium. [51]: character 40.

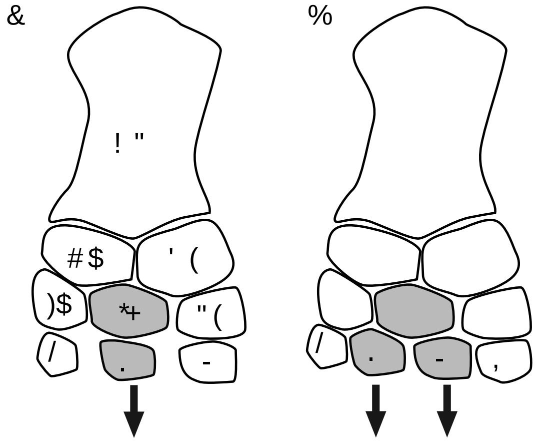

Partial forefin in dorsal view illustrating character 71.

72. **Zeugo- to autopodial elements**: flattened and plate-like (0), strongly thickened (1). [83]: character 94.
73. **Compact and tightly packed epi- and mesopodial rows:** absent, elements are loosely connected (0), present (1).
74. **Tightly packed rectangular phalanges**: absent, phalanges are mostly rounded (0), present (1) [83]: character 102, modified.
75. **Digital bifurcation**: absent (0), frequently occurs in digit IV (1). [51]: character 43.
76. **Manual digit V**: lost or reduced to small floating elements (0), present (1) [45]: character 73, modified.
77. **Forelimb–hind limb ratio**: nearly equal (0), forelimb longer twice as much as hind limb [220]: character 5, modified by [4].

Pelvic girdle and hind fin

78. **Ischium-pubis fusion in adults**: absent or minute (0), present with an obturator foramen (1); present with no obturator foramen (2) [221]: character 13, modified by [51].

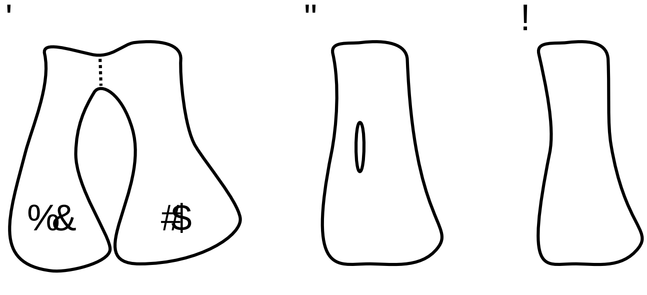

Ischium and pubis in lateral view illustrating character 78.

79. **Ischium or ischiopubis shape**: plate-like, flattened (0), rod-like (1) [45]: character 87, modified by [51].

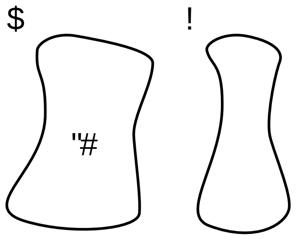

Ischium (or ischiopubis) in lateral view illustrating character 79.

80. **Iliac anteromedial prominence**: absent (0), present (1) [45]: character 84.

81. **Ilium proximal region:** expanded (0), narrow proximally and distally, rib-like (1) [219]: character 106, modified by [128].

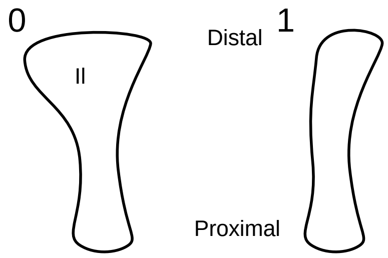

Ilium in lateral view illustrating character 81.

82. **Prominent, ridge-like dorsal and ventral processes demarcated from the head of the femur and extending up to mid-shaft:** absent (0), present (1). [51] : character 46.

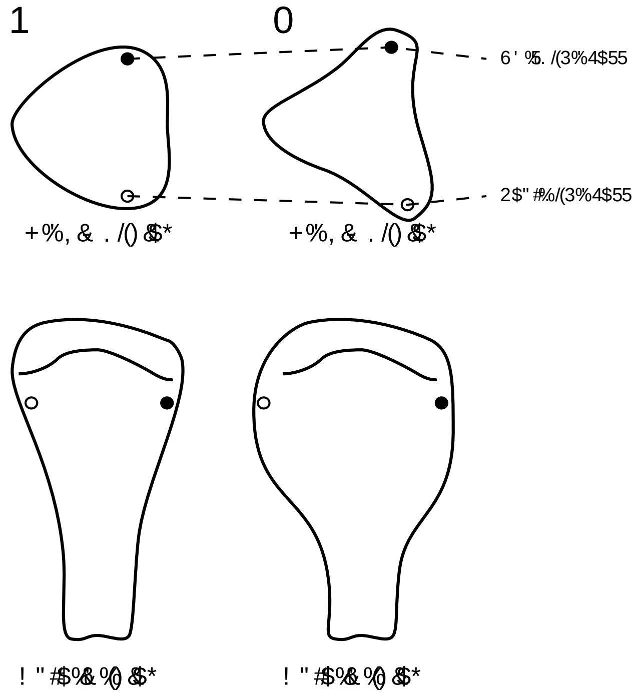

Femur in proximal (above) and anterior (below) views illustrating character 82.

83. **Wide distal femoral blade**: present (0), absent, the distal extremity of the femur being smaller than the proximal one in dorsal view (1).

84. **Astragalus/femoral contact**: absent (0), present (1) [100]: character 33.

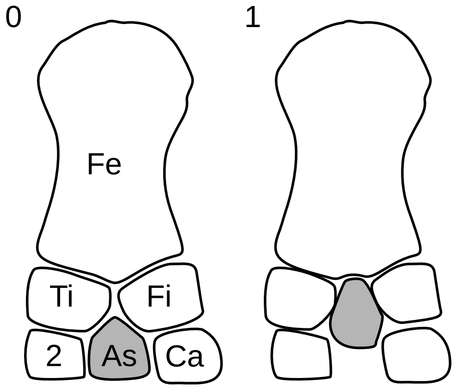

Partial hind fin in dorsal view illustrating character 84.

85. **Femur anterodistal facet for accessory zeugopodial element anterior to tibia**: absent (0), present (1). [51] : character 48.

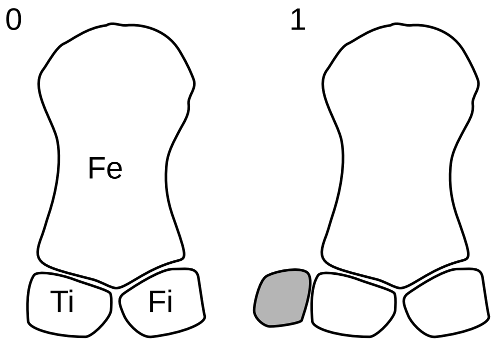

Partial hind fin in dorsal view illustrating character 85.

86. **Spatium interosseum between tibia and fibula**: present (0), absent (1). [83]: character 114, modified.
87. **Hind fin leading edge element in adults**: notched (0), straight (1). [45] : character 92, modified by [4].
88. **Postaxial accessory digit**: absent (0), present (1). [51]: character 50.

Maximum parsimony analytical details. Maximum parsimony analyses were carrier both in TNT v1.1[222] and PAUP* v4.0a142[223]. We used the exact parsimony searches of TNT 1.1 to analyse the character matrix (20,000 trees in memory, max ram=1000, heuristic search, tree bisection reconnection (TBR) as swapping algorithm with 10 trees saved per replication) and calculate the Bremer support (‘suboptimal’=5), Jacknife (removal probability = 36, with 1000 replications), and bootstrap (standard, 1000 replications) values. We timescaled and plotted our consensus tree using various branch length reconstruction methods (‘basic’, ‘equal’, ‘minimum’; see details below) and calculated stratigraphic congruence using a RCI and GER indexes using the packages `ape` v3.2[23] and `strap` v1.4[7] in R v.3.1.3[224].

As analyses of ophthalmosaurid relationship are characterised by moderately high homoplasy[3,4], we also ran a maximum parsimony analysis using implied weighting in TNT (K=3).

Analytical details of the Bayesian analyses. We used MrBayes v3.2.4[225]. Characters 33, 34 and 78 were ordered, as in the maximum parsimony analysis. Coding was considered as informative (reflecting the exclusion of autapomorphies) and we set used the following parameters: gamma rates and uncorrelated relaxed clock (igr). Our root calibration assumes Parvipelvia originated after the Permian but before the end of the Early Triassic (uniform distribution between 252.17 and 247.2 Ma) and we calibrated each tip using a uniform

distribution of first appearance datum ages to account for uncertainty in dating (except for a few taxa dated as a the ammonite zone or subzone, whose ages were obtained in Scott[226] and set as fixed). We set four chains, three replicate runs and 40,000,000 generations, sampling every 1000; a burn-in of 25% was applied.

BIODIVERSITY DATA

Time bins

We divided the largest stages (Aptian and Albian) into their widely accepted substages (lower and upper Aptian; lower, middle, and upper Albian), based on ammonite stratigraphy[12,227–232]. The lower Aptian encompasses the ammonite zones from the *oglanlensis* Zone to the *furcata* Zone; the upper Aptian from *subdonosocostatum* Zone to the *Jacobi* Zone; the lower Albian from the *schrammeni*/*tardefurcata* Zone to the *mammlilatum*/*auritiformis* Zone; the middle Albian to the *dentatus* Zone to the *lautus* Zone; the upper Albian from the *cristatum* Zone to the *dispar*/*briacensis* Zone. Using numerical ages from Kuhnt & Moullade[233], Scott[231] and the 2014 updated data of Cohen et al.[234], time bins for the stages from the Hettangian to the Turonian have a mean duration 5.06 My, and a moderate standard deviation (± 2.25 My).

Disparity

We use the R packages strap `ape` v3.2[23] to run the principal coordinate analyses on the phylogeny-reconstructed dataset (using Mesquite[235]), applying the Cailliez correction for negative eigenvalues.

PCOA

See nexus file ("phy_rec.nex") for the phylogenetically-reconstructed dataset and the files "pcoa.txt" and "pcoa.csv" for the PCoA results.

ECOLOGICAL DIVERSITY

Note on tooth wear quantification

We used articulated rostra to count the relative occurrence of three stages of wear that we defined qualitatively as follows: (i) no wear, the crown apex is pointed and still possesses its

enamel microtexture; (ii) slight wear, the crown apex is rounded and the microtexture of the enamel is lost; (iii) intense wear, the crown apex is broken and/or spalled and this section is polished and smoothed by further food processing, so that we are confident this feature is not diagenetic or due to preparation damage. We gave a weight to each category (1, 2, 3 respectively) and quantified wear as the relative proportion of each wear stage multiplied by its weight.

Ecological metrics employed

1. Absolute tooth size
    a. Mid-rostrum tooth, total apico-basal size
    b. In mm; e.g. 55
2. Crown shape ratio
    a. Crown apicobasal height divided by crown basal diameter (at the start of enamel covering)
    b. E.g. 1.65
3. Crown relative size
    a. Crown apicobasal height divided by basioccipital diameter (which is a good proxy for intraquadrate length/gullet size
    b. E.g. 0.304
4. Relative symphysial length
    a. Symphysis length divided by mandible length
    b. In %, e.g. 41
5. Relative snout depth (from McGowan[27])
    a. Snout depth at midpoint divided by jaw length
    b. E.g. 0.484
6. Absolute sclerotic aperture
    a. Diameter of the aperture (=inner opening) of the sclerotic ring
    b. In mm; e.g. 31.5
7. Tooth wear
    a. Assign a weight to each wear stage of each functional (=fully erupted) crown:
        i. 1=pristine: with details of texture intact and/or apex pointed
        ii. 2=polished: crown texture lost and/or apex slightly rounded
        iii. 3=heavy wear: crown apex (or more) broken off and the break is polished so that we are sure this is a diagenetic/preparation artefact

b. Value is the sum of % of each stage; e.g. 0.5*1+025*2+0.25*3=1.75

Confidence assessment. Because we restricted our data to ecologically relevant measurements and with a strong emphasis on Cretaceous forms, the resulting dataset is small and contain a non-negligible proportion of missing values (33%), which renders usual bootstrapping methods inadequate. To cope with this issue, we assessed the statistical support of our cluster using the "Approximately Unbiased P-value" method of the pvclust v1.3-2 package[236] in R. This method employs multiscaled bootstrapping: instead of simply bootstrapping the dataset, it creates multiple datasets that are smaller, equal and larger than the original dataset. We ran it from 0.5 times to 5 times the size of the original dataset, with 0.1 increments and 10,000 bootstrap per increment.

CORRELATIONS

We used the `nlme` v3.1[237] and `AICcmodavg` v2.0[238] packages in R to compute the Akaike Information Criterion for finite sample sizes (AICc[239]). Results from the pairwise correlation tests and from the generalised least square tests, for both the Early Cretaceous and Full (Cretaceous) dataset can be found in the "Supplementary Data 8 Pairwise_results.xlsx" and "Supplementary Data 9 GLS_results.xlsx" files.

SUPPLEMENTARY REFERENCES

1. Huelsenbeck, J. P. Comparing the Stratigraphic Record to Estimates of Phylogeny. *Paleobiology* **20,** 470–483 (1994).

2. Wills, M. A. Congruence Between Phylogeny and Stratigraphy: Randomization Tests and the Gap Excess Ratio. *Syst. Biol.* **48,** 559–580 (1999).

3. Fischer, V. *et al.* New ophthalmosaurid ichthyosaurs from the European Lower Cretaceous demonstrate extensive ichthyosaur survival across the Jurassic-Cretaceous boundary. *PLoS One* **7,** e29234 (2012).

4. Fischer, V. *et al.* A basal thunnosaurian from Iraq reveals disparate phylogenetic origins for Cretaceous ichthyosaurs. *Biol. Lett.* **9,** 1–6 (2013).

5. Roberts, A. J., Druckenmiller, P. S., Sætre, G.-P. & Hurum, J. H. A New Upper Jurassic Ophthalmosaurid Ichthyosaur from the Slottsmøya Member, Agardhfjellet Formation of Central Spitsbergen. *PLoS One* **9,** e103152 (2014).

6. Arkhangelsky, M. S. & Zverkov, N. G. On a new ichthyosaur of the genus *Undorosaurus*. *Proc. Zool. Inst. RAS* **318,** 187–196 (2014).

7. Bell, M. a. & Lloyd, G. T. strap : an R package for plotting phylogenies against stratigraphy and assessing their stratigraphic congruence. *Palaeontology* **58,** 379–389 (2015).

8. Gabdullin, R. R. Rhythms of Upper Cretaceous Deposits in the Russian Plate, Northwestern Caucasus and Southwestern Crimea (Structure, Classification, Models of Formation). *Geology* (Moscow University, 2002).

9. Košťák, M. & Weise, F. Remarks to geographic distribution and phylogeny of the Upper Cretaceous belemnite genus Praectinocamax Naidin. *Acta Univ. Carolinae Geol.* **49,** 135–139 (2006).

10. Wilmsen, M., Niebuhr, B., Wood, C. J. & Zawischa, D. Fauna and palaeoecology of the Middle Cenomanian Praeactinocamax primus Event at the type locality, Wunstorf quarry, northern Germany. *Cretac. Res.* **28,** 428–460 (2007).

11. Rozhdestvenskiy, A. K. The study of Cretaceous Reptiles in Russia. *Paleontol. J.* **2,** 206–214 (1973).

12. Juignet, P. La transgression crétacée sur la bordure orientale du Massif armoricain.

Aptien, Albien, Cénomanien de Normandie et du Maine. Le stratotype du Cénomanien. **Thèse de d,** (Université de Caen, 1974).

13. Cookson, I. C. & Hughes, N. F. Microplankton from the Cambridge Greensand (mid-Cretaceous). *Palaeontology* **7,** 37–59 (1964).

14. Hopson, P. M. A stratigraphical framework for the Upper Cretaceous Chalk of England and Scotland with statements on the Chalk of Northern Ireland and the UK Offshore Sector. *Br. Geol. Surv. Res. Reports* **RR/05/01,** 1–102 (2005).

15. Hopson, P. M., Wilkinson, I. P. & Wood, M. A. A stratigraphical framework for the Lower Cretaceous of England. *Br. Geol. Surv. Res. Reports* **RR/08/03,** 1–87 (2008).

16. Bardet, N., Fischer, V. & Machalski, M. Large predatory marine reptiles from the Albian–Cenomanian of Annopol, Poland. *Geol. Mag.* 1–16 (2015). doi:10.1017/S0016756815000254

17. Hüsing, S. K., Deenen, M. H. L., Koopmans, J. G. & Krijgsman, W. Magnetostratigraphic dating of the proposed Rhaetian GSSP at Steinbergkogel (Upper Triassic, Austria): Implications for the Late Triassic time scale. *Earth Planet. Sci. Lett.* **302,** 203–216 (2011).

18. Wotzlaw, J.-F. *et al.* Towards accurate numerical calibration of the Late Triassic: High-precision U-Pb geochronology constraints on the duration of the Rhaetian. *Geology* **42,** 571–574 (2014).

19. Fischer, V., Clément, A., Guiomar, M. & Godefroit, P. The first definite record of a Valanginian ichthyosaur and its implication for the evolution of post-Liassic Ichthyosauria. *Cretac. Res.* **32,** 155–163 (2011).

20. Ogg, J. G., Ogg, G. & Gradstein, F. M. *A concise geologic timescale*. *Cambridge University Press* (2008).

21. Hampe, O. Considerations on a *Brachauchenius* skeleton (Pliosauroidea) from the lower Paja Formation (late Barremian) of Villa de Leyva area (Colombia). *Foss. Rec. — Mitteilungen aus dem Museum dür Naturkd. Berlin, Geowissenschaften* **8,** 37–51 (2005).

22. Druckenmiller, P. S. & Maxwell, E. E. A Middle Jurassic (Bajocian) ophthalmosaurid (Reptilia, Ichthyosauria) from the Tuxedni Formation, Alaska and the early

diversification of the clade. *Geol. Mag.* **151,** 41–48 (2014).

23. Paradis, E., Claude, J. & Strimmer, K. APE: Analyses of phylogenetics and evolution in R language. *Bioinformatics* **20,** 289–290 (2004).

24. Bapst, D. W. paleotree: an R package for paleontological and phylogenetic analyses of evolution. *Methods Ecol. Evol.* **3,** 803–807 (2012).

25. Kirton, A. M. A review of British Upper Jurassic ichthyosaurs. **Ph.D. diss,** (University of Newcastle upon Tyne, 1983).

26. Gilmore, C. W. Osteology of *Baptanodon* (Marsh). *Mem. Carnegie Museum* **II,** 77–129 (1905).

27. McGowan, C. The description and phenetic relationships of a new ichthyosaur genus from the Upper Jurassic of England. *Can. J. Earth Sci.* **13,** 668–683 (1976).

28. Fernández, M. & Talevi, M. Ophthalmosaurian (Ichthyosauria) records from the Aalenian–Bajocian of Patagonia (Argentina): an overview. *Geol. Mag.* **151,** 49–59 (2014).

29. Bardet, N. & Fernández, M. A new ichthyosaur from the Upper Jurassic lithographic limestones of Bavaria. *J. Paleontol.* **74,** 503–511 (2000).

30. Kear, B. P., Boles, W. E. & Smith, E. T. Unusual gut contents in a Cretaceous ichthyosaur. *Proc. R. Soc. London B Biol. Sci.* **270,** S206–S208 (2003).

31. Kear, B. P. Cranial morphology of *Platypterygius longmani* Wade, 1990 (Reptilia: Ichthyosauria) from the Lower Cretaceous of Australia. *Zool. J. Linn. Soc.* **145,** 583–622 (2005).

32. Wade, M. A review of the Australian Cretaceous longipinnate ichthyosaur *Platypterygius* (Ichthyosauria, Ichthyopterygia). *Mem. Queensl. Museum* **28,** 115–137 (1990).

33. Kuhn, O. Ein skelett von *Ichthyosaurus hercynicus* n. sp. aus dem Aptien von Gitter. *Berichte der Naturforschenden Gesellschaft Bamb.* **29,** 69–82 (1946).

34. Romer, A. S. An ichthyosaur skull from the Cretaceous of Wyoming. *Contrib. to Geol. Wyoming Univ.* **7,** 27–41 (1968).

35. Paramo, M. E. *Platypterygius sachicarum* (Reptilia, Ichthyosauria) nueva especie del

Cretácio de Colombia. *Rev. Ingeominas* **6,** 1–12 (1997).

36. Fischer, V., Bardet, N., Guiomar, M. & Godefroit, P. High Diversity in Cretaceous Ichthyosaurs from Europe Prior to Their Extinction. *PLoS One* **9,** e84709 (2014).

37. Haq, B. U. Cretaceous eustasy revisited. *Glob. Planet. Change* **113,** 44–58 (2014).

38. Prokoph, A., Shields, G. A. & Veizer, J. Compilation and time-series analysis of a marine carbonate δ18O, δ13C, 87Sr/86Sr and δ34S database through Earth history. *Earth-Science Rev.* **87,** 113–133 (2008).

39. Martin, J. E., Amiot, R., Lécuyer, C. & Benton, M. J. Sea surface temperature contributes to marine crocodylomorph evolution. *Nat Commun* **5,** 1–7 (2014).

40. Bardet, N. Un crâne d'Ichthyopterygia dans le Cénomanien du Boulonnais. *Mémoires la Société académique du Boulonnais* **6,** 1–32 (1989).

41. Adams, T. L. & Fiorillo, A. *Platypterygius* Huene, 1922 (Ichthyosauria, Ophthalmosauridae) from the Late Cretaceous of Texas, USA. *Palaeontol. Electron.* **14,** 19A (2011).

42. Choo, B. Cretaceous ichthyosaurs from Western Australia. *Rec. West. Aust. Museum, Suppl.* **57,** 207–218 (1999).

43. Kear, B. P. Cretaceous marine reptiles of Australia: a review of taxonomy and distribution. *Cretac. Res.* **24,** 277–303 (2003).

44. Blainville de, H. M. D. Description de quelques espèces de reptiles de la Californie, précédée de l'analyse d'un système général d'érpetologie et d'amphibiologie. *Nouv. Ann. du Muséum d'Histoire Nat. Paris* **4,** 233–296 (1835).

45. Motani, R. Phylogeny of the Ichthyopterygia. *J. Vertebr. Paleontol.* **19,** 473–496 (1999).

46. Baur, G. On the morphology and origin of the Ichthyopterygia. *Am. Nat.* **21,** 837–840 (1887).

47. Fischer, V., Arkhangelsky, M. S., Uspensky, G. N., Stenshin, I. M. & Godefroit, P. A new Lower Cretaceous ichthyosaur from Russia reveals skull shape conservatism within Ophthalmosaurinae. *Geol. Mag.* **151,** 60–70 (2014).

48. Arkhangelsky, M. S. The historical sequence of Jurassic and Cretaceous ichthyosaurs.

Paleontol. J. **35,** 521–524 (2001).

49. Fernández, M. A new ichthyosaur from the Tithonian (Late Jurassic) of the Neuquén Basin (Argentina). *J. Paleontol.* **71,** 479–484 (1997).

50. Ochev, V. G. & Efimov, V. M. A new genus of Ichthyosaur from the Ul'Yanovsk area of the Povolzh'ye Region. *Paleontol. J.* **4,** 87–91 (1985).

51. Fischer, V., Masure, E., Arkhangelsky, M. S. & Godefroit, P. A new Barremian (Early Cretaceous) ichthyosaur from western Russia. *J. Vertebr. Paleontol.* **31,** 1010–1025 (2011).

52. Huene, F. von. Beitrag zur Kenntnis mariner mesozoicher Wirbeltiere in Argentinien. *Cent. für Mineral. Geol. und Paläntologie, B* **1927,** 22–29 (1927).

53. Broili, F. Ein neuer Ichthyosaurus aus der norddeutschen Kreide. *Palaeontographica* **54,** 139–162 (1907).

54. Druckenmiller, P. S. & Maxwell, E. E. A new Lower Cretaceous (lower Albian) ichthyosaur genus from the Clearwater Formation, Alberta, Canada. *Can. J. Earth Sci.* **47,** 1037–1053 (2010).

55. Maxwell, E. E. & Caldwell, M. W. A new genus of ichthyosaur from the Lower Cretaceous of Western Canada. *Palaeontology* **49,** 1043–1052 (2006).

56. M'Coy, F. On the occurrence of Ichthyosaurus and Plesiosaurus in Australia. *Ann. Mag. Nat. Hist. third Ser.* **19,** 355–356 (1867).

57. Nace, R. L. A new ichthyosaur from the Upper Cretaceous Mowry Formation of Wyoming. *Am. J. Sci.* **237,** 673–686 (1939).

58. Seeley, H. G. *Index of the fossil remains of Aves, Ornithosauria and Reptilia, from the Secondary System of Strata Aranged in the Woodward Museum of the Univeristy of Cambridge*. (1869).

59. Carter, J. Notice of the jaws of an Ichthyosaurus from the chalk in the neighbourhood of Cambridge. *Reports Br. Assoc. Adv. Sci.* **1845,** 60 (1846).

60. Arkhangelsky, M. S. On the ichthyosaurian genus *Platypterygius*. *Paleontol. J.* **32,** 611–615 (1998).

61. Broili, F. Ichthyosaurierreste aus der Kriede. *Neues Jahrb. für Mineral. Geol. und*

Paläontologie. Beilage **25,** 422–442 (1908).

62. Eichwald, K. E. Einige paläontologische Bemerkungen über den Eisensand von Kursk. *Bull. la Société Impériale des Nat. Moscou* **2,** 209–231 (1853).
63. Eichwald, K. E. *Lethaea Rossica ou Paléontologie de la Russie. Second Volume. Période Moyenne*. (1865).
64. Merriam, J. C. The types of limb-structure in the Triassic Ichthyosauria. *Am. J. Sci. Fourth Ser.* **19,** 23–30 (1905).
65. Merriam, J. C. Triassic Ichthyopterygia from California and Nevada. *Univ. Calif. Publ. Bull. Dep. Geol.* **3,** 63–108 (1902).
66. Kuhn, O. *Ichthyosauria*. *Fossilium Catalogus I: Animalia* **63,** (W. Junk, 1934).
67. Kuhn, O. *Sauropterygia*. *Fossilium Catalogus I: Animalia* **69,** (W. Junk, 1934).
68. Kiprijanoff, W. Studien über die fossilen Reptilien Russlands. Theil 1, Gattung Ichthyosaurus König aus dem severischen Sandstein oder Osteolith der Kreide-Gruppe. *Mémoires l'Académie impériale des Sci. St.-Pétersbourg, VIIe série* **28,** 1–103 (1881).
69. Kiprijanoff, W. Studien uber die fossilen Reptilien Russlands. 2. Theil. Gattung Plesiosaurus Conybeare aus dem Sewerschen Sandstein oder Osteolith der Keridegruppe. *Mémoires l'Académie impériale des Sci. St.-Pétersbourg, VIIe série* **30,** 1–55 (1882).
70. Kiprijanoff, W. Studien uber die fossilen Reptilien Russlands. 3. Theil. Gruppe Thaumatosauria n. Aus der Kreide-Formation und dem Moskauer Jura. *Mémoires l'Académie impériale des Sci. St.-Pétersbourg, VIIe série* **31,** 1–57 (1883).
71. Kiprijanoff, W. Studien uber die fossilen Reptilien Russlands. 4. Theil. Ordung Crocodilia Oppel. Indeterminirte fossile Reptilien . *Mémoires l'Académie impériale des Sci. St.-Pétersbourg, VIIe série* **31,** 1–29 (1883).
72. McGowan, C. The systematics of Cretaceous ichthyosaurs with particuliar reference to the material from North America. *Contrib. to Geol.* **11,** 9–29 (1972).
73. Koken, E. Die Reptilien der norddeutschen unteren Kreide. *Zeitschrift der Dtsch. Geol. Gesellschaft* **35,** 735–827 (1883).
74. Meyer von, H. Ichthyosaurus strombecki aus dem Eisenstein der unteren Kreide bei

Gross-Döhren. *Palaeontographica* **10,** (1862).

75. Storrs, G. W., Arkhangelsky, M. S. & Efimov, V. M. in *The Age of Dinosaurs in Russia and Mongolia* (eds. Benton, M. J., Shiskin, M. A., Unwin, D. M. & Kurochkin, E. N.) 187–210 (Cambridge University Press, 2000).

76. Bogolubov, N. N. Sur quelques restes de deux reptiles (*Cryptoclidus simbirskensis* n. sp. et *Ichthyosaurus steleodon* n. sp.) trouvés par Mr. le Profess. P. Pavlow sur les bords de la Volga dans les couches mesozoïques de Simbirsk. *Annu. géologique minéralogique Russ.* **11,** 42–64 (1909).

77. Scheyer, T. M. & Moser, M. Survival of the thinnest: rediscovery of Bauer's (1898) ichthyosaur tooth sections from Upper Jurassic lithographic limestone quarries, south Germany. *Swiss J. Geosci.* **104,** S147–S157 (2011).

78. Cornuel, M. J. Note sur deux portions de mâchoire fossile rapportées à un Gavial et recueillies dans le terrain crétacé inférieur du département de la Haute-Marne. *Bull. La société géologique Fr. série 2* **8,** 170–174 (1851).

79. Cornuel, M. J. Description de débris de poissons fossiles provenant principalement du calcaire néocomien du département de la Haute-Marne. *Bull. la Société géologique Fr. série 3* **5,** 604–626 (1877).

80. Cornuel, M. J. Note sur les ossements fossiles découverts dans le calcaire de néocomien de Wassy (Haute-Marne). *Bull. la Société géologique Fr. deuxième série* **7,** 702–704 (1850).

81. Lapparent, A. F. de & Stchepinsky, V. Les Iguanodons de la région de Saint-Dizier (Haute-Marne). *Comptes Rendus l'Académie des Sci. Paris, série D* **266,** 1370–1372 (1968).

82. Efimov, V. M. A new genus of ichthyosaurs from the Late Cretaceous of the Ulyanovsk Volga region. *Paleontol. J.* **31,** 422–426 (1997).

83. Maisch, M. W. & Matzke, A. T. The Ichthyosauria. *Stuttgarter Beiträge zur Naturkd. Ser. B (Geologie und Paläontologie)* **298,** 1–159 (2000).

84. Fischer, V. *et al. Simbirskiasaurus* and *Pervushovisaurus* reassessed: implications for the taxonomy and cranial osteology of Cretaceous platypterygiine ichthyosaurs. *Zool. J. Linn. Soc.* **171,** 822–841 (2014).

85. Martin, K. Ein Ichthyosaurus von Ceram. *Jaarb. van het Mijnwezen, Ned. Oost-Indië* **17,** 3–19 (1888).

86. Godefroit, P. Les grands ichthyosaures sinémuriens d'Arlon. *Bull. l'Institut R. des Sci. Nat. Belgique Sci. la Terre* **63,** 25–71 (1993).

87. Maisch, M. W., Reisdorf, A., Schlatter, R. & Wetzel, A. A large skull of *Ichthyosaurus* (Reptilia: Ichthyosauria) from the Lower Sinemurian (Lower Jurassic) of Frick (NW Switzerland). *Swiss J. Geosci.* **101,** 617–627 (2008).

88. Vincent, P. *et al.* Mary Anning's legacy to French vertebrate palaeontology. *Geol. Mag.* **151,** 7–20 (2014).

89. Maxwell, E. E., Caldwell, M. W. & Lamoureux, D. O. Tooth histology in the Cretaceous ichthyosaur *Platypterygius australis* , and its significance for the conservation and divergence of mineralized tooth tissues in amniotes. *J. Morphol.* **272,** 129–135 (2011).

90. Maxwell, E. E., Caldwell, M. W. & Lamoureux, D. O. Tooth histology, attachment, and replacement in the Ichthyopterygia reviewed in an evolutionary context. *Paläontologische Zeitschrift* **86,** 1–14 (2012).

91. Arkhangelsky, M. S., Averianov, A. O., Pervushov, E. M., Ratnikov, V. Y. & Zozyrev, N. Y. On ichthyosaur remains from the Cretaceous of the Voronezh region. *Paleontol. J.* **42,** 287–291 (2008).

92. Kear, B. P. & Zammit, M. In utero foetal remains of the Cretaceous ichthyosaurian *Platypterygius*: ontogenetic implications for character state efficacy. *Geol. Mag.* **151,** 71–86 (2014).

93. Carter, J. On the occurence of a new species of Ichthyosaurus in the Chalk. *London Geol. J.* **1,** (1846).

94. Sauvage, H. E. Recherches sur les reptiles trouvées dans le Gault de l'Est du bassin de Paris. *Mémoires la Société géologique Fr. 3e série* **2,** 21–24 (1882).

95. Buffetaut, E. Remarques préliminaires sur l'ichthyosaure de Saint-Jouin (76). *Bull. la Société Géologique Normandie Amis du Muséum du Havre* **64,** 17–19 (1977).

96. Buffetaut, E. *et al.* Les vertébrés de la partie moyenne du Crétacé en Europe. *Cretac. Res.* **2,** 275–281 (1981).

97. Buffetaut, E., Tomasson, R. & Tong, H. Restes fossiles de grands reptiles jurassiques et crétacés dans l'Aube (France). *Bull. d'information des géologues du bassin Paris* **40,** 33–43 (2003).

98. McGowan, C. & Motani, R. *Part 8. Ichthyopterygia*. *Handbook of Paleoherpetology* **8,** (Verlag Dr. Friedrich Pfeil, 2003).

99. Arkhangelsky, M. S. & Averianov, A. O. On the find of a primitive hadrosaurid dinosaur (Ornitishia, Hadrosauroidea) in the Cretaceous of the Belgorod Region. *Paleontol. J.* **37,** 58–61 (2003).

100. Maxwell, E. E. Generic reassignment of an ichthyosaur from the Queen Elizabeth Islands, Northwest Territories, Canada. *J. Vertebr. Paleontol.* **30,** 403–415 (2010).

101. Owen, R. *A monograph on the fossil Reptilia of the Cretaceous formations*. (The Palæntological Society, 1851).

102. Milner, A. C. in *Fossils of the Chalk* (eds. Owen, E. & Smith, A. B.) **2,** 266–280 (The Palaeontological Association field guides to fossils, 1987).

103. Morière, J. Découverte d'une tête incomplète de saurien dans un bloc de craie tombée de la partie supérieure de la falaise située entre Auberville et Villers-sur-mer. *Bull. la Société Linéenne Normandie, troisième série* **1,** 129–130 (1877).

104. Blain, H.-A., Pennetier, G. & Pennetier, E. Présence du genre *Platypterygius* (Ichthyosauria, Reptilia) dans le Cénomanien inférieur de Villers-sur-Mer (Normandie, France. *Echos des falaises* **7,** 35–50 (2003).

105. Zawischa, D. Saurierzähne aus Wunstorf. *Arbeitskr. Paläontologie Hann.* **10,** 16–17 (1982).

106. Wittler, F. A. & Roth, R. Ein Ichthyosaurier aus dem Cenoman des Münsterlandes. *Arbeitskr. Paläontologie Hann.* **29,** 76–81 (2001).

107. Wittler, F. A. Besonderheiten aus der Oberkreide von Dortmund I: Ein Ichthyosaurierzahn aus dem südwestfälischen Cenoman. *Dortmunder Beitrage zue Landeskunde—Naturwissenschaftliche Mitteilungen* **42,** 59–61 (2010).

108. Diedrich, C. G. New ichthyosaur remains of Platypterygius cf. campylodon (Carter 1846) (Ichthyopterygia, Reptilia) from the Cenomanian of NW Germany. *Münstersche Forschungen zur Geol. und Paläontologie* **93,** 97–108 (2002).

109. Bardet, N., Wellnhofer, P. & Herm, D. Discovery of ichthyosaur remains (Reptilia) in the upper Cenomanian of Bavaria. *Mitteilungen der Bayer. Staatssammlung für Paläontologie und Hist. Geol.* **34,** 213–220 (1994).

110. Bardet, N. Stratigraphic evidence for the extinction of the ichthyosaurs. *Terra Nov.* **4,** 649–656 (1992).

111. Capellini, G. Ichthyosaurus campylodon e tronchi di cicadee nelle argille scagliose dell emilia. *Mem. della R. Accad. delle Sci. di Bol. Ser. IV* **10,** 431–450 (1890).

112. Sirotti, A. & Papazzoni, C. On the Cretaceous ichthyosaur remains from the Northern Apennines (Italy). *Boll. della Soc. Paleontol. Ital.* **41,** 237–248 (2002).

113. Machalski, M., Komorowski, A. & Harasimiuk, M. Nowe perspektywy poszukiwan morskich kregowców kredowych w nieczynnej kopalni fosforytów w Annopolu nad Wisla. *Prz. Geol.* **57,** 1–4 (2009).

114. Pervushov, E. M., Arkhangelsky, M. S. & Ivanov, A. V. *Catalog of the locations of the remainders of sea reptiles in the Jurassic and Cretaceous of the Lower Volga Region*. (Saratov University, 1999).

115. Merriam, J. C. The occurrence of ichthyosaur-like remains in the Upper Cretaceous of Wyoming. *Science (80-.).* **22,** 640–641 (1905).

116. Gilmore, C. W. A second occurence of ichthyosaurian remains in the Benton Cretaceous. *Science (80-.).* **39,** 210 (1914).

117. Slaughter, B. H. & Hoover, B. R. Occurences of Ichthyosaurian Remains in the Cretaceous of Texas. *Texas J. Sci.* **15,** 339–343 (1963).

118. Nace, R. L. A new ichthyosaur from the Late Cretaceous of northeastern Wyoming. *Am. J. Sci.* **239,** 908–914 (1941).

119. Maxwell, E. E. & Kear, B. P. Postcranial anatomy of *Platypterygius americanus* (Reptilia: Ichthyosauria) from the Cretaceous of Wyoming. *J. Vertebr. Paleontol.* **30,** 1059–1068 (2010).

120. Lydekker, R. Indian pre-Tertiary Vertebrata. Fossil Reptilia and Batrachia. *Mem. Geol. Surv. India* **1,** 1–36 (1879).

121. Lydekker, R. Note on the classification of the Ichthyopterygia with a notice of two new species. *Geol. Mag. third Ser.* **5,** 309–314 (1888).

122. Underwood, C. J., Goswami, A., Prasad, G. V. R., Verma, O. & Flynn, J. J. Marine vertebrates from the 'Middle' Cretaceous (Early Cenomanian) of South India. *J. Vertebr. Paleontol.* **31,** 539–552 (2011).

123. Verma, O. Cretaceous vertebrate fauna of the Cauvery Basin, southern India: Palaeodiversity and palaeobiogeographic implications. *Palaeogeogr. Palaeoclimatol. Palaeoecol.* (2015). doi:10.1016/j.palaeo.2015.04.021

124. Maxwell, E. E. New metrics to differentiate species of *Stenopterygius* (Reptilia: Ichthyosauria) from the Lower Jurassic of southwestern Germany. *J. Paleontol.* **86,** 105–115 (2012).

125. McGowan, C. A new and typically Jurassic ichthyosaur from the Upper Triassic of Birtish Columbia. *Can. J. Earth Sci.* **33,** (1996).

126. Fischer, V. New data on the ichthyosaur *Platypterygius hercynicus* and its implications for the validity of the genus. *Acta Palaeontol. Pol.* **57,** 123–134 (2012).

127. Druckenmiller, P. S., Hurum, J., Knutsen, E. M. & Nakrem, H. A. Two new ophthalmosaurids (Reptilia: Ichthyosauria) from the Agardhfjellet Formation (Upper Jurassic: Volgian/Tithonian), Svalbard, Norway. *Nor. J. Geol.* **92,** 311–339 (2012).

128. Maxwell, E. E., Fernández, M. S. & Schoch, R. R. First diagnostic marine reptile remains from the Aalenian (Middle Jurassic): a new ichthyosaur from southwestern Germany. *PLoS One* **7,** e41692 (2012).

129. Maxwell, E. E., Zammit, M. & Druckenmiller, P. S. Morphology and orientation of the ichthyosaurian femur. *J. Vertebr. Paleontol.* **32,** 1207–1211 (2012).

130. Motani, R. On the evolution and homologies of ichthyosaurian forefins. *J. Vertebr. Paleontol.* **19,** 28–41 (1999).

131. Maisch, M. W. & Matzke, A. T. The cranial osteology of the ichthyosaur Leptonectes </i>cf.<i> tenuirostris from the Lower Jurassic of England. *J. Vertebr. Paleontol.* **23,** 116–127 (2003).

132. Motani, R. True skull roof configuration of *Ichthyosaurus* and *Stenopterygius* and its implications. *J. Vertebr. Paleontol.* **25,** 338–342 (2005).

133. Fernández, M. Dorsal or ventral? Homologies of the forefin of *Caypullisaurus* (Ichthyosauria: Ophthalmosauria). *J. Vertebr. Paleontol.* **21,** 515–520 (2001).

134. Motani, R. *et al.* First evidence of centralia in Ichthyopterygia reiterating bias from paedomorphic characters on marine reptile phylogenetic reconstruction. *J. Vertebr. Paleontol.* 1–6 (2015). doi:10.1080/02724634.2014.948547

135. Benson, R. B. J., Butler, R. J., Lindgren, J. & Smith, A. S. Mesozoic marine tetrapod diversity: mass extinctions and temporal heterogeneity in geological megabiases affecting the vertebrates. *Proc. R. Soc. B Biol. Sci.* **277,** 829–834 (2010).

136. Benson, R. B. J. & Butler, R. J. in *Comparing the geological and fossil records: implications for biodiversity studies* (eds. McGowan, A. J. & Smith, A. B.) **358,** 191–208 (Geological Society, Special Publications, 2011).

137. Gasparini, Z. & Fernández, M. in *The Neuquén Basin, Argentina: A case study in sequence stratigraphy and basin dynamics* (eds. Veiga, G. D., Spalletti, L. A., Howell, J. A. & Schwarz, E.) **252,** 279–294 (Geological Society, special Publications, 2005).

138. Fernández, M. in *Patagonian Mesozoic Reptiles* (eds. Gasparini, Z., Salgado, L. & Coria, R. A.) 271–291 (Indiana University Press, 2007).

139. Maisch, M. W. & Matzke, A. T. *Mikadocephalus gracilirostris* n. g. n. sp., a new ichthyosaur from the Grenzbitumenzone (Anisian-Ladinian) of Monte San Giorgio (Switzerland). *Paläontologische Zeitschrift* **71,** 267–289 (1997).

140. Maisch, M. W. Observations on Triassic ichthyosaurs; Part V, The skulls of Mikadocephalus and Wimanius reconstructed. *Neues Jahrbuch für Geologie und Paläontologie. Monatshefte* **1999,** 345–356 (1999).

141. McGowan, C. A remarkable small ichthyosaur from the Upper Triassic of British Columbia, representing a new genus and species. *Can. J. Earth Sci.* **32,** 292–303 (1995).

142. McGowan, C. An ichthyosaur forefin from the Triassic of British Columbia exemplifying Jurassic features. *Can. J. Earth Sci.* **28,** 1553–1560 (1991).

143. von Hillebrandt, A. & Krystyn, L. On the oldest Jurassic ammonites of Europe (Northern Calcareous Alps, Austria) and their global significance. *Neues Jahrb. für Geol. und Paläontologie, Abhandlungen* **253,** 163–195 (2009).

144. Benson, R. B. J., Evans, M. & Druckenmiller, P. S. High diversity, low disparity and small body size in plesiosaurs (Reptilia, Sauropterygia) from the Triassic–Jurassic

boundary. *PLoS One* **7,** e31838 (2012).

145. Maisch, M. W. & Reisdorf, A. G. Evidence for the longest stratigraphic range of a post-Triassic Ichthyosaur: a *Leptonectes tenuirostris* from the Pliensbachian (Lower Jurassic) of Switzerland. *Geobios* **39,** 491–505 (2006).

146. Godefroit, P. Présence de *Leptopterygius tenuirostris* (Reptilia, Ichthyosauria) dans le Lias moyen de Lorraine belge. *Bull. l'Institut R. des Sci. Nat. Belgique Sci. la Terre* **62,** 163–170 (1992).

147. Conybeare, W. D. Additional notes on the fossil genera *Ichthyosaurus* and *Plesiosaurus*. *Trans. Geol. Soc. London* **2,** 103–123 (1822).

148. McGowan, C. Computed tomography reveals further details of *Excalibosaurus*, a putative ancestor for the swordfish-like ichthyosaur *Eurhinosaurus*. *J. Vertebr. Paleontol.* **9,** 269–281 (1989).

149. McGowan, C. A putative ancestor for the swordfish-like ichthyosaur *Eurhinosaurus*. *Nature* **322,** 454–456 (1986).

150. McGowan, C. A new specimen of *Excalibosaurus* from the English Lower Jurassic. *J. Vertebr. Paleontol.* **23,** 950–956 (2003).

151. Reisdorf, A., Maisch, M. W. & Wetzel, A. First record of the leptonectid ichthyosaur *Eurhinosaurus longirostris* from the Early Jurassic of Switzerland and its stratigraphic framework. *Swiss J. Geosci.* **104,** 211–224 (2011).

152. Godefroit, P. Les reptiles marins du Toarcien (Jurassique inférieur) belgo-luxembourgeois. *Mémoires pour Serv. à l'Explication des Cart. Géologiques Minières la Belgique* **39,** 98 (1994).

153. Lamaud, P. Les Ichthyosaures et la mer toarcienne du Pic Saint-Loup. *Minéraux Foss. Le Guid. du Collect.* **58,** 42–49 (1979).

154. Pharisat, A., Contini, D. & Frikert, J.-C. Early Jurassic (Lower Toarcian) 'ichthyosaurs' from France-Comté, France. *Rev. Paléobiologie, Vol. spécial* **7,** 189–198 (1993).

155. Pharisat, A. L'ichthyosaure de la base des schistes-cartons du Toarcien inférieur de Noirefontaine (Doubs). *Société d'Histoire Nat. du Pays Montbéliard* **1993,** 193–198 (1993).

156. Fischer, V., Guiomar, M. & Godefroit, P. New data on the palaeobiogeography of Early Jurassic marine reptiles: the Toarcian ichthyosaur fauna of the Vocontian Basin (SE France). *Neues Jahrb. für Geol. und Paläontologie* **261,** 111–127 (2011).

157. Huene, F. von. *Die Ichthyosaurier des Lias und ihre Zusammenhänge*. *Monographien zur Geologie und Paläontologie* **1,** (Verlag von Gebrüder Borntraeger, 1922).

158. Huene, F. von. Ein neuer Fund von *Eurhinosaurus longirostris*. *Neues Jahrb. für Geol. und Paläontologie, Abhandlungen* **93,** 277–283 (1951).

159. McGowan, C. A revision of the Lower Jurassic ichthyosaurs of Germany with descriptions of two new species. *Palaeontogr. Abteilung A. Paläozoologie, Stratigr.* **166,** 93–135 (1979).

160. Jäger, G. über eine neue species von Ichthyosauren (*Ichthyosaurus longirostris* Owen & Jäger). Nebst Bemerkungen über die übrigen in der Liasformation Würtembergs aufgefundenen Reptiliien. *Novum actorum Acad. caesarae Leopoldino-Carolinae naturae curiosorum* **25,** 937–967 (1856).

161. Maisch, M. W. A new ichthyosaur genus from the Posidonia Shale (Lower Toarcian, Jurassic) of Holzmaden, SW-Germany with comments on the phylogeny of post-Triassic ichthyosaurs. *Neues Jahrb. für Geol. und Paläontologie, Abhandlungen* **209,** 47–78 (1998).

162. Maisch, M. W. Neue Exemplare der seltenen Ichthyosauriergattung *Suevoleviathan* Maisch 1998 aus dem Unteren Jura von Südwestdeutschland. *Geol. Palaeontol.* **35,** 145–160 (2001).

163. Martin, J. E., Fischer, V., Vincent, P. & Suan, G. A longirostrine *Temnodontosaurus* (Ichthyosauria) with comments on Early Jurassic ichthyosaur niche partitioning and disparity. *Palaeontology* **55,** 995–1005 (2012).

164. McGowan, C. A revision of the longipinnate ichthyosaurs of the Lower Jurassic of England, with description of the new species (Reptilia, Ichthyosauria). *Life Sci. Contrib. R. Ontario Museum* **97,** 1–37 (1974).

165. McGowan, C. *Temnodontosaurus risor* is a juvenile of *T. platyodon* (Reptilia: Ichthyosauria). *J. Vertebr. Paleontol.* **14,** 472–479 (1994).

166. Maisch, M. W. & Hungerbühler, A. New evidence for a discrete supratemporal bone in

the Jurassic Ichthyosaur *Temnodontosaurus*. *Hist. Biol.* **15,** 335–345 (2001).

167. Maisch, M. W. A braincase of *Temnodontosaurus nuertingensis* cf. *trigonodon* (von Theodori, 1843) (Ichthyosauria) from the Lower Jurassic of Germany. *Geol. Palaeontol.* **36,** 115–122 (2002).

168. Gaudry, A. L'*Ichthyosaurus burgundiæ*. *Bull. la Société d'Histoire Nat. d'Autun* **5,** 1–9 (1892).

169. Lydekker, R. *Catalogue of the fossil Reptilia and Amphibia in British Museum (Natural History). Part II. containing the orders Ichthyopterygia and Sauropterygia*. (Printed by Orders of the Trustees of the British Museum, London, 1889).

170. Maisch, M. W. Revision der Gattung *Stenopterygius* Jaekel, 1904 emend. von Huene, 1922 (Reptilia: Ichthyosauria) aus dem unteren Jura Westeuropas. *Palaeodiversity* **1,** 227–271 (2008).

171. Caine, H. & Benton, M. J. Ichthyosauria from the upper Lias of Strawberry Bank, England. *Palaeontology* **54,** 1069–1093 (2011).

172. Bennett, S. P. *et al.* A new specimen of *Ichthyosaurus communis* from Dorset, UK, and its bearing on the stratigraphical range of the species. *Proc. Geol. Assoc.* **123,** 146–154 (2012).

173. Godefroit, P. Un crâne d'*Ichthyosaurus communis* (Reptilia, Ichthyosauria) du Sinémurien supérieur de Lorraine belge. *Bull. la Société belge Géologie* **104,** 77–89 (1996).

174. Sollas, W. J. The skull of Ichthyosaurus, studied in serial sections. *Philos. Trans. R. Soc. London, Ser. B* **208,** 63–126 (1916).

175. Delair, J. B. Unusual preservation of fibrous elements in an ichthyosaur skull. *Nature* **212,** 575–576 (1966).

176. McGowan, C. The cranial morphology of the Lower Liassic latipinnate ichthyosaurs of England. *Bull. Br. Museum (Natural Hist. Geol.* **24,** 1–109 (1973).

177. de la Beche, H. T. & Conybeare, W. D. Notice of the discovery of a new fossil animal, forming a link between the *Ichthyosaurus* and Crocodile, together with general remarks on the Osteology of the Ichthyosaurus. *Trans. Geol. Soc. London* **5,** 559–594 (1821).

178. Dechaseaux, C. L'arrière-crâne d'un ichthyosaurien du Lias. *Ann. Paléontologie* **40,**

67–77 (1954).

179. Maisch, M. W. & Ansorge, J. The Liassic ichthyosaur *Stenopterygius* cf. *S. quadrissicus* from the lower Toarcian of Dobbertin (NE Germany) and some considerations on lower Toarcian marine reptile palaeobiogeography. *Paläontologische Zeitschrift* **78,** 161–171 (2004).

180. Quenstedt, F. A. *Der Jura*. (1856).

181. Spalletti, L., Gasparini, Z. & Fernández, M. Facies, ambientes y reptiles marinos de la transición entre las formaciones Los Molles y Lajas (Jurasico medio), cuenca nequina, Argentina. *Acta Geol. Leopoldensis* **39,** 329–344 (1994).

182. Fernández, M. A new long-snouted ichthyosaur from the early Bajocian of Neuquén basin (Argentina). *Ameghiniana* **31,** 291–297 (1994).

183. Bardet, N. *et al.* Découverte de l'ichthyosaure *Ophthalmosaurus* dans le Tithonien (Jurassique supérieur) du Boulonnais, Nord de la France. *Neues Jahrb. für Geol. und Paläontologie, Abhandlungen* **205,** 339–354 (1997).

184. Seeley, H. G. On the pectoral arch and fore limb of *Ophthalmosaurus*, a new ichthyosaurian genus from the Oxford Clay. *Q. J. Geol. Soc. London* **30,** 696–707 (1874).

185. Andrews, C. W. Note on the osteology of *Ophthalmosaurus icenicus* Seeley an ichthyosaurian Reptile from the Oxford Clay of Peterborough. *Geol. Mag.* **4,** 202–208 (1910).

186. Andrews, C. W. *A descriptive catalogue of the Marine Reptiles of the Oxford Clay, part II.* (British Museum of Natural History, 1913).

187. Appleby, R. M. The osteology and taxonomy of the fossil reptile *Ophthalmosaurus*. *Proc. Zool. Soc. London* **126,** 403–447 (1956).

188. Appleby, R. M. On the cranial morphology of ichthyosaurs. *Proc. Zool. Soc. London* **137,** 333–370 (1961).

189. Maisch, M. W. Variationen im Verlauf der Gerhinnerven bei *Ophthalmosaurus* (Ichthyosauria, Jura). *Neues Jahrb. für Geol. und Paläontologie, Monatshefte* **1997,** 425–433 (1997).

190. Maisch, M. W. The temporal region of the Middle Jurassic ichthyosaur

Ophthalmosaurus: further evidence for the non-diapsid cranial architecture of the Ichthyosauria. *Neues Jahrb. für Geol. und Paläontologie. Monatshefte* **1998,** 401–414 (1998).

191. Araújo, R., Smith, A. S. & Liston, J. The Alfred Leeds fossil vertebrate Collection of the National Museum of Ireland–Natural History. *Irish J. Earth Sci.* **26,** 17–32 (2008).

192. Massare, J. A. & Young, H. A. Gastric contents of an ichthyosaur from the Sundance formation (Jurassic) of central Wyoming. *Paludicola* **5,** 20–27 (2005).

193. Massare, J. A., Buchholtz, E. A., Kenney, J. & Chomat, A.-M. Vertebral morphology of *Ophthalmosaurus natans* (Reptilia: Ichthyosauria) from the Jurassic Sundance Formation of Wyoming. *Paludicola* **5,** 242–254 (2006).

194. Wahl, W. R. Taphonomy of a nose dive: bone and tooth displacement and mineral accretion in an ichthyosaur skull. *Paludicola* **7,** 107–116 (2009).

195. Marsh, O. C. A new Order of Extinct Reptiles (Sauronodonta), from the Jurassic Formation of the Rocky Mountains. *Am. J. Sci. Third Ser.* **17,** 85–86 (1878).

196. Gilmore, C. W. Discovery of teeth in *Baptanodon*, an ichthyosaurian from the Jurassic of Wyoming. *Science (80-.).* **16,** 913–914 (1902).

197. Gilmore, C. W. Notes on osteology of *Baptanodon*. *Mem. Carnegie Museum* **II,** 325–337 (1906).

198. Gilmore, C. W. New species of *Baptanodon*. *Am. J. Sci. Fourth Ser.* **23,** 193–198 (1907).

199. Maxwell, E. E. & Druckenmiller, P. S. A small ichthyosaur from the Clearwater Formation (Alberta, Canada) and a discussion of the taxonomic utility of the pectoral girdle. *Paläontologische Zeitschrift* **85,** 457–463 (2011).

200. Fernández, M. A new ichthyosaur from the Los Molles Formation (Early Bajocian), Neuquén basin, Argentina. *J. Paleontol.* **73,** 677–681 (1999).

201. Boulenger, G. A. On a new species of ichthyosaur from Bath. *Proc. Zool. Soc. London* **1904,** 424–426 (1904).

202. McGowan, C. The taxonomic status of the Late Jurassic ichthyosaur *Grendelius mordax*: a preliminary report. *J. Vertebr. Paleontol.* **17,** 428–430 (1997).

203. Sauvage, H. E. Catalogue des reptiles trouvées dans le terrain jurassique supérieur du Boulonnais. *Comptes rendus l'Association française pour l'avancement des Sci.* **28,** 416–419 (1899).

204. Lennier, G. Description des fossiles du Cap de la Hève. *Bull. la Société géologique Normandie* **12,** 17–98 (1887).

205. Valenciennes, M. A. D'une tête de grand Ichthyosaure, trouvée dans l'argile de Kimmeridge par M. Lennier, au cap de la Hève, près du Havre. *C. R. Hebd. Seances Acad. Sci.* **53,** 276–273 (1861).

206. Russell, D. A. in *The Geology of Melville Island, Artic Canada* (eds. Christie, R. L. & McMilan, N. J.) **450,** 195–201 (Bulletin of the Geological Survey of Canada, 1993).

207. Fernández, M. S. & Maxwell, E. E. The genus *Arthropterygius* Maxwell (Ichthyosauria: Ophthalmosauridae) in the Late Jurassic of the Neuquén Basin, Argentina. *Geobios* **45,** 535–540 (2012).

208. Zverkov, N. G., Arkhangelsky, M. S., Pardo-Pérez, J. & Beznosov, P. A. On the Upper Jurassic ichthyosaur remains from the Russian North. *Proc. Zool. Inst. RAS* **319,** 81–97 (2015).

209. Fernández, M. Redescription and phylogenetic position of *Caypullisaurus* (Ichthyosauria: Ophthalmosauridae). *J. Paleontol.* **81,** 368–375 (2007).

210. Wagner, A. Die Characteristic einer neuen Art von *Ichthyosaurus* aus den lithographischen Schiefern und eines Zahnes von *Polyptychodon* aus dem Gründsandsteine von Kelheim. *Bull. der königlische Akad. der Wissenschaft, Gelehrt. Anzeigen* **3,** 25–35 (1853).

211. Meyer von, H. Ichthyosaurus leptospondylus aus dem lithographischen Schiefer von Eichstätt. *Palaeontographica* **11,** 222–225 (1863).

212. Fraas, E. E. *Ichthyosaurier der Süddeutschen Trias und Jura-Ablagerungen*. (H. Laupp, 1891).

213. Gasparini, Z. *et al.* Reptiles from Lithographic Limestones of the Los Catutos Member (Middle-Upper Tithonian), Neuquén Province, Argentina: an essay on its taxonomic composition and preservation in an environmental and geographic context. *Ameghiniana* **52,** 1–28 (2015).

214. Zammit, M. A review of Australasian ichthyosaurs. *Alcheringa* **34,** 281–292 (2010).

215. Wade, M. Platypterygius australis, an Australian Cretaceous ichthyosaur. *Lethaia* **17,** 99–113 (1984).

216. Zammit, M., Norris, R. M. & Kear, B. P. The Australian Cretaceous ichthyosaur *Platypterygius australis*: a description and review of postcranial remains. *J. Vertebr. Paleontol.* **30,** 1726–1735 (2010).

217. Kolb, C. & Sander, P. M. Redescription of the ichthyosaur *Platypterygius hercynicus* (Kuhn 1946) from the Lower Cretaceous of Salzgitter (Lower Saxony, Germany). *Palaeontogr. Abteilung A (Paläozoologie, Stratigr.* **288,** 151–192 (2009).

218. Hoedemaeker, P. J. On the Barremian - lower Albian stratigraphy of Colombia. *Scr. Geol.* **128,** 3–15 (2004).

219. Sander, P. M. Ichthyosauria: their diversity, distribution, and phylogeny. *Paläontologische Zeitschrift* **74,** 1–35 (2000).

220. Godefroit, P. The skull of *Stenopterygius longifrons* (Owen, 1881). *Rev. Paléobiologie Genève Vol. spécial* **7,** 67–84 (1993).

221. Mazin, J.-M. Affinités et phylogénie des Ichthyopterygia. *Geobios, mémoire spécial* **6,** 85–98 (1982).

222. Goloboff, P., Farris, J. & Nixon, K. T.N.T. 1.1: Tree Analysis Using New Technology. Available at www.zmuc.dk/public/phylogeny/TNT/. (2010).

223. Swofford, D. L. PAUP*. Phylogenetic Analysis Using Parsimony (*and Other Methods). Version 4. (2002).

224. R Core Team. R: A language and environment for statistical computing. (2015).

225. Ronquist, F. & Huelsenbeck, J. P. MRBAYES 3: Bayesian phylogenetic inference under mixed models. *Bioinformatics* **19,** 1572–1574 (2003).

226. Scott, R. W. A Cretaceous chronostratigraphic database: construction and applications. *Notebooks Geol.* **14,** 15–37 (2014).

227. Travassac, F. Stratigraphie, sédimentologie et géochimie d'une série d'âge barrémien supérieur à albien pro parte du bassin vocontien (SE France) : implications paléoenvironnmentales. **DEA,** (Ecole doctorale Sciences de l'Environnement d'Aix-

Marseille, 2004).

228. Amédro, F. Support for a Vraconnian Stage between the Albien sensu stricto and the Cenomanien (Cretacous System). *Notebooks Geol.* **Memoir 200,** 83 (2008).

229. Amédro, F. & Robaszynski, F. Zonation by ammonites and foraminifers of the Vraconnian-Turonian interval: A comparison of the Boreal and Tethyan domains (NW Europe / Central Tunisia). *Notebooks Geol. Lett.* **2008/02,** 5 (2008).

230. Lehmann, J., Heldt, M., Bachmann, M. & Hedi Negra, M. E. Aptian (Lower Cretaceous) biostratigraphy and cephalopods from north central Tunisia. *Cretac. Res.* **30,** 895–910 (2009).

231. Scott, R. W. Uppermost Albian biostrigraphy and chronostratigraphy. *Notebooks Geol.* **2009/03,** 1–16 (2009).

232. Owen, H. G. The Gault Group (Early Cretaceous, Albian), in East Kent, S.E. England; its lithology and ammonite biozonation. *Proc. Geol. Assoc.* **123,** 742–765 (2012).

233. Kuhnt, W. & Moullade, M. The Gargasian (Middle Aptian) of La Marcouline section at Cassis-La Bédoule (SE France): Stable isotope record and orbital cyclicity. *Notebooks Geol.* **2007/02,** 1–9 (2007).

234. Cohen, K. M., Finney, S. C., Gibbard, P. L. & Fan, J. The ICS International Chronostratigraphic Chart. *Episodes* **36,** 199–204 (2013).

235. Maddison, W. P. & Maddison, D. R. Mesquite: A modular sytem for evolutionary analysis. (2011).

236. Suzuki, R. & Shimodaira, H. Hierarchical Clustering with P-Values via Multiscale Bootstrap Resampling. 1–13 (2014).

237. Pinheiro, J., Bates, D., DebRoy, S. & Sarkar, D. Package 'nlme'. (2015).

238. Mazerolle, M. J. Package 'AICcmodavg'. (2015).

239. Burnham, K. P. & Anderson, D. *Model Selection and Multi-Model Inference: A Practical Information- Theoretic Approach.* (Springer, 2001).
